# Reaction Time Variability and Brain White Matter Integrity

**DOI:** 10.1037/neu0000483

**Published:** 2019-07

**Authors:** Tom Booth, Dominika Dykiert, Janie Corley, Alan J. Gow, Zoe Morris, Susana Muñoz Maniega, Natalie A. Royle, Maria del C. Valdés Hernández, John M. Starr, Lars Penke, Mark E. Bastin, Joanna M. Wardlaw, Ian J. Deary

**Affiliations:** 1 Centre for Cognitive Ageing and Cognitive Epidemiology, and Department of Psychology, The University of Edinburgh; 2 Centre for Cognitive Ageing and Cognitive Epidemiology, and Department of Psychology, The University of Edinburgh; and Anna Freud National Centre for Children and Families, London, United Kingdom; 3 Centre for Cognitive Ageing and Cognitive Epidemiology and Department of Psychology, The University of Edinburgh; 4 Centre for Cognitive Ageing and Cognitive Epidemiology, The University of Edinburgh, and Department of Psychology, School of Life Sciences, Heriot-Watt University, Edinburgh; 5 Brain Research Imaging Centre, Division of Neuroimaging Sciences, The University of Edinburgh; 6 Centre for Cognitive Ageing and Cognitive Epidemiology, and Brain Research Imaging Centre, Division of Neuroimaging Sciences, The University of Edinburgh; 7 Centre for Cognitive Ageing and Cognitive Epidemiology, and Geriatric Medicine Unit, The University of Edinburgh; 8 Centre for Cognitive Ageing and Cognitive Epidemiology, The University of Edinburgh, and Biological Personality Psychology and Psychological Assessment, Georg Elias Muller Institute of Psychology, Georg August University Gottingen; 9 Centre for Cognitive Ageing and Cognitive Epidemiology, and Brain Research Imaging Centre, Division of Neuroimaging Sciences, The University of Edinburgh; 10 Brain Research Imaging Centre, Division of Neuroimaging Sciences, The University of Edinburgh; 11 Centre for Cognitive Ageing and Cognitive Epidemiology, and Department of Psychology, The University of Edinburgh

**Keywords:** white matter hyperintensities, diffusion tensor imaging, reaction time, intraindividual variability, cognitive aging

## Abstract

***Objective:*** Mean speed of responding is the most commonly used measure in the assessment of reaction time (RT). An alternative measure is intraindividual variability (IIV): the inconsistency of responding across multiple trials of a test. IIV has been suggested as an important indicator of central nervous system functioning, and as such, there has been increasing interest in the associations between IIV and brain imaging metrics. Results however, have been inconsistent. The present seeks to provide a comprehensive evaluation of the associations between a variety of measures of brain white matter integrity and individual differences in choice RT (CRT) IIV. ***Method:*** MRI brain scans of members of the Lothian Birth Cohort 1936 were assessed to obtain measures of the volume and severity of white matter hyperintensities, and the integrity of brain white matter tracts. CRT was assessed with a 4 CRT task on a separate occasion. Data were analyzed using multiple regression (*N* range = 358–670). ***Results:*** Greater volume of hyperintensities and more severe hyperintensities in frontal regions were associated with higher CRT IIV. White matter tract integrity, as assessed by both fractional anisotropy and mean diffusivity, showed the smallest effect sizes in associations with CRT IIV. Associations with hyperintensities were attenuated and no longer significant after controlling for *M* CRT. ***Conclusions:*** Taken together, the results of the present study suggested that IIV was not incrementally predictive of white matter integrity over mean speed. This is in contrast to previous reports, and highlights the need for further study.

## General Scientific Summary

Variability in speeded cognitive test performance has been argued to be a potential early marker of cognitive decline and progression into mild cognitive impairment in aging. Evidence as to the robustness of the relationship, and the potential neurological underpinnings is varied. Our results suggest that average speeded performance, not variability, may be more reliably related to various measures of the brain. These findings are in contrast to much of the extant literature, highlighting the need for further research.

Speed of information processing and reaction time (RT) have been studied as integral parts of human cognitive capacities since the 19th century (Cattell & Galton, 1890), with interest persisting over the subsequent years (Deary, 2000; Diehl, Hooker, & Sliwinsky, 2015). It has been proposed that speed of basic information processing represents a fundamental and tractable element of human general cognitive abilities (Jensen, 2006), which has led to a huge degree of interest in studying its neurological basis (Eckert, 2011; Penke et al., 2010, 2012). Processing speed has also been suggested to be a key capacity in the study of cognitive aging (Madden, 2001; Salthouse, 1996). Classically, RT studies have focused on some measure of central tendency, or average speed over trials. More recently however, focus has switched to how speed of responding may vary across a set of trials (Hultsch, MacDonald, & Dixon, 2002). Within-individual variability in RT is correlated with average RT, but debate remains as to which is the most fundamental, and whether they share neuroanatomical correlates.

Intraindividual variability (IIV) in cognitive assessment indexes the consistency of a person’s responses across a short period of time. In the context of an RT task, IIV is the amount of trial-to-trial variability. It provides a complement to the more widely used mean (or other index of central tendency) RT across a number of trials. IIV is a trait-like characteristic of an individual, in that people more variable on one cognitive task are also more variable on different tasks, and those more variable within a testing occasion are also more variable across occasions (Hultsch, MacDonald, Hunter, Levy-Bencheton, & Strauss, 2000). IIV is significantly correlated with higher level cognitive functioning; for example, with general mental ability (Deary, Der, & Ford, 2001; Rabbitt, Osman, Moore, & Stollery, 2001), with less variable people tending to have higher cognitive ability. There is a growing interest in IIV due, in part, to its predictive value. IIV predicts change in cognitive abilities over time (Lövdén, Li, Shing, & Lindenberger, 2007; MacDonald, Hultsch, & Dixon, 2003; Nilsson, Thomas, O’Brien, & Gallagher, 2014) and mortality (Deary & Der, 2005a). Furthermore, IIV differentiates between groups of different neurological health statuses, for example, mild cognitive impairment (MCI) versus no-MCI (Dixon et al., 2007), and dementia versus no dementia (Hultsch et al., 2000). People with MCI or dementia are, on average, slower and more variable, and there is some evidence that IIV has predictive value over and above that of RT mean (Dixon et al., 2007; Hultsch et al., 2000).

IIV increases with age from young adulthood (see Dykiert, Der, Starr, & Deary, 2012; Hultsch, Strauss, Hunter, & MacDonald, 2008, for reviews). The mechanisms underpinning this age effect are not well understood. The simplest explanation for the increased IIV is that it is driven by general slowing that occurs with age. In other words, as mean RT increases, so does the IIV. However, a number of researchers have argued that the increasing of IIV is the primary phenomenon which, in turn, leads to an increased mean RT. Several theories have been proposed as possible mechanisms of IIV; for example, higher frequency of attentional blocks (Bunce, Warr, & Cochrane, 1993) or lapses of intention (West, Murphy, Armilio, Craik, & Stuss, 2002), which are related to poorer executive functioning. The lapses or blocks lead to very long RTs on trials on which they occur, thus increasing the overall IIV. Naturally, these long RTs also lead to an increase in mean RT.

A primary focus of research examining the biological basis of RT and IIV has been on the brain. Life-course changes in IIV (decrease in childhood, relative stability in adulthood and an increase in older age) closely map onto the maturation and degeneration of the brain across the life span (MacDonald, Nyberg, & Bäckman, 2006). Specifically, accumulating evidence from recent imaging studies has highlighted brain white matter and its integrity as potentially important for RT.

Table 1 summarizes the results from a number of recent studies which have considered the associations between IIV in a RT task and metrics derived from brain imaging. A few generalities may be taken from the content of Table 1. First, across modalities, there is some evidence that the effects of IIV may be independent of mean RT (e.g., Jackson, Balota, Duchek, & Head, 2012; Walhovd & Fjell, 2007). Second, in studies focused on brain volume measures, there has been some consistency in effects located in the frontal white matter (Bunce et al., 2007; Haynes et al., 2017; Lövdén et al., 2013). Studies focused on white matter connectivity have also found support for the importance of frontal associations (Fjell, Westlye, Amlien, & Walhovd, 2011; Moy et al., 2011).[Table-anchor tbl1]

Although these results, on face value, suggest a consistency of effects observed across studies, the issue of whether RT variability is related to white matter (WM) micro- and macrostructure is far from resolved. There is much heterogeneity in the studies described here, in particular with reference to the RT tasks on which intraindividual variability is calculated; the measures of WM integrity adopted; and the size, age, and make-up of the samples.

Variability in cognitive performance is not a unitary concept. Even the same measure, for example individual standard deviation, may represent qualitatively different aspects of human performance, depending on the task or the timescale from which it was derived. For example, there are different components to different tasks, such as perceptual, motor, reasoning, decision making, or inhibition of irrelevant response. Variability in some of these components might have different neural underpinnings. Variability in CRT, which is a relatively simple task requiring only a minimal amount of cognitive processing (a selection and execution of an appropriate response) may not be readily comparable to variability in a task, which might still use RT as its “score” but requires more complex cognitive operations (e.g., inhibition of an irrelevant response or performing operations in WM). Consistent with this notion, there are reports of different IIV-WM integrity associations from tasks of different difficulty (e.g., Bunce et al., 2007; Deary et al., 2006; Fjell et al., 2011; Haynes et al., 2017; Mella et al., 2013). Of note is that both higher and lower associations have been reported for more complex tasks. Further, Fjell et al. (2011) demonstrated that associations of IIV and measures of WM integrity differed, not only in magnitude but also in spatial distribution in the brain, depending on whether congruent (less demanding) or incongruent (more demanding) trials were selected for the calculation of IIV. Given these findings we propose that the question of whether there is a relationship between WM micro/macrostructure needs to be addressed by a series of studies focused on specific RT tasks.

A second important consideration is the size and structure of the samples used in the extant research. The problem of low power in studies with small samples is generally accepted, insofar as underpowered studies are less likely to detect a true effect (i.e., they are more likely to produce false negatives). However, two issues associated with small samples that are underappreciated are that (a) even the effects that are found to be significant, are less likely to reflect a true effect and (b) the effect sizes of significant results are more likely to be overestimated (Button et al., 2013). Sample sizes of studies reviewed in Table 1 vary from 25 to 526. For reference, a sample size required to achieve 80% power to detect a small (*r* = .1) or medium (*r* = .3) correlation at α = .05 would be 782 and 84, respectively. This is clearly an oversimplification of the process of appropriate power calculations, however consider in light of these estimates, none of the studies reviewed were sufficiently powered to detect a small effect and more than half had insufficient power to detect a moderate effect. Finally, and as noted above, age plays an important role in the relationship between RT IIV and brain integrity. Fjell et al. (2011) found that the association between white matter microstructure and IIV increases with age, with stronger associations found among older participants (age ≥52 years) than younger participants (age <52 years). Moy et al. (2011) found that effects for radial diffusivity (RD) and mean diffusivity (MD) were no longer significant after controlling for age (range = 20–66+ years in their sample). Given the potential complexity of the relationship with age, birth cohort studies may be particularly useful as they provide built in control for age effects.

In the current study, we seek to provide a comprehensive assessment of the associations between a variety of volumetric and connective brain imaging metrics and variability in a single task (CRT task) in a large birth cohort, thus minimizing the influence of test comparison, age effects and small sample estimate inconsistency.

## Method

### Participants

Participants in the current study were from the Lothian Birth Cohort 1936 (LBC1936), a longitudinal study of cognitive aging. The study sample consists of surviving members of the Scottish Mental Survey 1947, most of whom were tested on an IQ-type test at school at approximately age 11 years. The LBC1936 participants were mostly resident in Edinburgh and its surrounding area (i.e., the Lothians, Scotland) at recruitment to Wave 1 of the study at about age 70 years. Study protocols for initial recruitment and subsequent waves of data collection, including brain MRI, are reported in detail elsewhere (Deary, Gow, Pattie, & Starr, 2012; Deary et al., 2007; Wardlaw et al., 2011).

The LBC1936 sample consisted of 1,091 participants in Wave 1 (age *M* = 69.5 years, *SD* = 0.8), of whom 866 participants returned in Wave 2 (age *M* = 72.5 years, *SD* = 0.7). Of these, 855 were invited to undertake MRI, of which 728 initiated MRI protocol. For the current study, we retained only those participants for whom quantitative estimates of WMH volume were available. Reasons for absence of WMH data included the use of shortened sequencing protocol with some participants who were uncertain or anxious, termination of scan prematurely due to issues such as claustrophobia, poor quality data due to movement artifacts, and a number of other health and safety reasons (for discussion of some of these issues see Sandeman et al., 2013). The resultant total study sample comprised 671 participants (see Table 2 for sample demographics). All data used in the current cross-sectional study come from Wave 2 of testing.[Table-anchor tbl2]

### CRT Variability and Mean

CRT mean and variability were measured using a portable device designed for the U.K. Health and Lifestyle Survey (Cox, Huppert, & Whichelow, 1993). The box includes a high-contrast LCD display and five response keys labeled *1*, *2*, *0*, *3*, *4* arranged in a shallow arc. In the four CRT task, participants placed their second and middle fingers of each hand on the buttons labeled *1*, *2*, *3*, and *4*. Participants were presented with a number (1, 2, 3, or 4) on the LCD screen and had to press the corresponding button as quickly as possible. The test consisted of a total of 48 trials: eight practice trials and 40 test trials. Within the test trials, each of the numbers (1, 2, 3, and 4) appeared 10 times in a random order. The time between trials varied randomly from 1 s to 3 s across all trials.

The box provides the mean and standard deviation of both the correct and incorrect responses; however, only data from the correct responses were available for the current study. Deary and Der (2005b) reported the test–retest reliability of *M* CRT and *SD* CRT based on a sample of 49 adults (age *M* = 37.1 years, *SD* = 11.4). *M* CRT had a test–retest stability of 0.92, whereas for *SD* CRT the test–retest reliability was 0.73.

In the current analyses, our primary variable of interest is *SD* CRT. However, for comparison purposes with previous studies, we also report results for models in which the dependent variable is the coefficient of variability (*CV*; *CV* = *SD* CRT/*M* CRT). As is expected, these three variables show moderate to high positive correlations (*M* CRT and *SD* CRT: *r* = .62; *M* CRT and *CV* CRT: *r* = 0.16; *SD* CRT and *CV* CRT: *r* = 0.86).

### Image Acquisition

Wardlaw et al. (2011) provide full details of the brain imaging protocol. In brief, participants underwent whole brain structural and high angular resolution 2-mm isotropic voxel diffusion MRI (7 T2- and 64 diffusion-weighted (*b* = 1,000 s/mm2) axial single-shot spin-echo echo-planar imaging volumes) on a GE Signa Horizon HDxt 1.5T clinical scanner (General Electric, Milwaukee, WI), using a self-shielding gradient set (maximum gradient strength 33 mT/m) and an eight-channel phased-array head coil. The structural MRI included axial T2- (1-mm × 1-mm × 2-mm voxels), T2*- (1-mm × 1-mm × 2-mm voxels) and FLAIR-weighted (1-mm × 1-mm × 4-mm voxels) scans, and a high resolution T1-weighted volume scan (1-mm × 1-mm × 1.3-mm voxels) acquired in the coronal plane. All sequences were collected with contiguous slice locations, whereas the acquisition parameters for the T2-, T2*-, FLAIR and diffusion MRI protocols, that is, field-of-view (256 mm × 256 mm in all cases), imaging matrix, slice thickness and location, were chosen to allow easier coregistration between scans.

### Quantitative White Matter Hyperintensity (WMH) Volumes

Prior to image segmentation, all structural scans were coregistered using FLIRT, a linear automatic registration tool from the FMRIB Software Library (http://www.fmrib.ox.ac.uk/fsl). We used a validated multispectral image processing tool, MCMxxxVI (Valdés Hernández, Ferguson, Chappell, & Wardlaw, 2010; Wardlaw et al., 2011; http://sourceforge.net/projects/bric1936) for segmentation of brain tissue volumes from the four structural scans, that is, T2-, T1-, T2*- and FLAIR-weighted MRI, to measure: intracranial volume (all soft tissue structures inside the cranial cavity including brain, dural, cerebrospinal fluid and venous sinuses); total brain volume (the actual brain tissue volume without the superficial or ventricular cerebrospinal fluid); cerebrospinal fluid (all cerebrospinal fluid inside the cranial cavity including the ventricles and superficial subarachnoid space); and WMH volumes. MCMxxxVI does not distinguish hyperintense and hypointense areas of cerebromalacea due to old cortical/subcortical infarcts or lacunes from WMH and cerebrospinal fluid, respectively. Therefore, these areas were masked out from the respective binary masks by thresholding in FLAIR sequences using a region-growing algorithm from Analyze 10.0 (http://www.analyzedirect.com/Analyze). Where stroke lesions were confluent with WMH, the boundary between the two was determined by evaluation of the WMH in the contralateral hemisphere and neuroradiological knowledge. Brain tissue volumes were measured blind to participant information. For the current study, we use the total WMH volume (cm^3^) after first residualizing for overall brain size (intracranial volume, cm^3^).

### Qualitative White Matter Lesion Location

Qualitative visual ratings of the intensity and location of WMH were scored using the Wahlund scale based on the FLAIR and T2-weighted scans (Wahlund et al., 2001). Hyperintensities were rated both bilaterally and as an overall score in the frontal, parieto-occipital and temporal lobes, as well as the basal ganglia and infratentorial regions. Hyperintensities were rated on a four-point scale: for basal ganglia: 0 = *no hyperintensities*; 1 = *one focal hyperintensity*; 2 = *more than one focal hyperintensity*; 3 = *confluent hyperintensities*; for other regions: 0 = *no hyperintensities*; 1 = *focal hyperintensity*; 2 = *beginning confluence hyperintensity*; 3 = *diffuse involvement of the entire region*) by Zoe Morris, a trained neuroradiologist. This process results in an ordered categorical variable. On inspection of the score distribution (see Table 2), it became clear there were very low frequency cells which would be problematic for the estimation of the association beta coefficients. As such, we created binary variables for each area by combining 0 and 1 and 1 and 2 scores from the original scale.

For the current analysis, we used the overall Wahlund ratings rather than considering the left and right hemispheres individually. Correlations between left and right hemispheres were moderate to high for all lobes and regions (frontal lobe = .86; parieto-occipital lobe = .89; temporal lobe = .64; basal ganglia = .72; infratentorial region = .88).

### Tract Segmentation

The diffusion MRI data were preprocessed using FSL tools (FMRIB, Oxford, UK; http://www.fmrib.ox.ac.uk) to extract the brain, remove bulk patient motion and eddy current induced artifacts, and generate parametric maps of fractional anisotropy (FA). Underlying connectivity data were generated using BedpostX/ProbTrackX with the default settings of a two-fiber model per voxel, and 5,000 probabilistic streamlines with a fixed separation of 0.5 mm between successive points (Behrens, Johansen Berg, Jbabdi, Rushworth, & Woolrich, 2007).

Twelve tracts of interest were identified using probabilistic neighborhood tractography, a novel approach for automatic and reproducible tract segmentation (Clayden, Storkey, & Bastin, 2007), as implemented in the TractoR package for fiber tracking analysis (Clayden et al., 2011; http://www.tractor-mri.org.uk). Briefly, this method works by segmenting the same fasciculus-of-interest across a group of subjects from single seed point tractography output by modeling how individual tracts compare to a predefined reference tract in terms of their length and shape (Clayden et al., 2007). In practice, multiple native space seed points are placed in a cubic neighborhood of voxels (typically 7 × 7 × 7) surrounding a seed point transferred from the center of the reference tract, which is defined in standard space, with the tract that best matches the reference chosen from this group of ‘candidate tracts’. Tracts assessed were the genu and splenium of corpus callosum, and bilateral anterior thalamic radiations, rostral cingulum bundles, arcuate, uncinate and inferior longitudinal fasciculi. Tract masks generated by probabilistic neighborhood tractography were overlaid on the FA parametric maps and tract-averaged values of these biomarkers, weighted by the connection probability, determined for each tract in every participant.

To ensure that the segmented tracts were anatomically plausible representations of the fasciculi of interest, a researcher (i.e., Susana Muñoz Maniega) visually inspected all masks blind to the other study variables and excluded tracts with aberrant or truncated pathways. In general, probabilistic neighborhood tractography was able to segment the 12 tracts of interest reliably (see Clayden, Storkey, Muñoz Maniega, & Bastin, 2009) in the majority of participants, with tracts that did not meet quality criteria, such as truncation or failing to follow the expected path, ranging from 0.3% for the splenium of corpus callosum to 16% for the left anterior thalamic radiation, with a mean of 5%. (Failures in tract segmentation are typically caused by underlying tractography errors in BedpostX/ProbTrackX resulting from finite image resolution, small registration mismatches in the component diffusion MRI volumes and measurement noise.)

From the point of view of substantive investigations, the 12 tracts represent a good balance between projection (anterior thalamic radiation), commissural (genu and splenium of the corpus callosum) and association (arcuate fasciculus, rostral cingulum, uncinated fasciculus & inferior longitudinal thalamic radiation) fibers which connect a wide variety of brain regions. In the current study, we used FA and MD data for each of the 12 tracts to compute a metric of overall white matter integrity following Penke et al. (2012).

Confirmatory factor analytic models were fit using full information maximum likelihood estimation to account for the small proportions of missing data in Mplus 7.4 (Muthén & Muthén, 1998–2012). A single general integrity factor was modeled, with all 12 tracts loading on it. Separate models were fit for FA (gFA) and MD (gMD). Values for the left and right hemispheres of each tract were allowed to correlate in order to account for the local dependence. Regression based factor scores were estimated from these models and used in subsequent analyses.

Both models showed good fit to the data (gFA: χ^2^ = 101.48, *df* = 49, *p*<.001; CFI = .98; TLI = .97; RMSEA = .040; SRMR = .033; gMD: χ^2^ = 193.43, *df* = 49, *p*<.001; CFI = .94; TLI = .93; RMSEA = .066; SRMR = .041) suggesting the suitability of the models. The general factors accounted for 9.5% to 47.7% (gFA) and 3.3% to 51.8% (gMD) of the variance in the individual tracts. Factor score determinacies, which provide a metric of score reliability, were high for both models. Determinacies are reported for each missing data pattern. For gFA, the complete case determinacy was 0.915, with the lowest determinacy 0.812. For gMD, the complete case determinacy was 0.925, with the lowest determinacy of 0.791.

In addition, and for information, we also ran models using the hemispheric tract average for each of the tracts listed above.

### Health Covariates

Participants were asked a series of questions on their medical history by an interviewer, which were responded to with simple Yes/No answers. The questions asked whether participants had a history of cardiovascular disease, hypertension (being treated for), high cholesterol, diabetes, blood circulation problems (being treated for), or stroke. In all statistical analyses (see next section), these variables were included as individual binary covariates. This was done to provide some statistical control for any shared variance between presence of disease, imaging metrics and speeded performance.

### Statistical Analyses

We built two regression models for each of our predictor variables of interest, WMH, general white matter integrity FA and MD, and Wahlund ratings of white matter lesion severity and location. In addition to the primary imaging variables of interest, we also include results from analyses using the hemispheric averages (or single values for the genu and splenium of the corpus callosum) for each white matter tract as predictors of speed variability in place of the general FA and MD variables.

In the first model (Model 1), we included age, sex, health covariates and the brain integrity measure of interest. We chose to include age as, despite the use of a birth cohort and thus a narrow age range, both RT and white matter are sensitive to effects of age. Correlations between age and the focal RT and white matter variables are provided in the online supplemental material. In the second model (Model 2), we additionally included *M* CRT. Here we were interested in the extent of attenuation of any main effects after controlling for average RT.

The primary focus of this study was in prediction of *SD* CRT as a measure of variability. However, for completeness and following reviewer comments, given the exploratory nature of the study, we also estimate models using both *M* CRT and the *CV* CRT as dependent variables. In the analyses of *M* CRT, the second stage models include *SD* CRT in the second step. Results for assumption checks for all models are included in the online supplemental material.

To assess the robustness of our findings, a number of sensitivity analyses were conducted. First, to assess the influence of outlying values, we reran our final models using robust regression using the Huber method as implemented in the *rlm* function in the MASS package in R (Venables & Ripley, 2002). Second, we reran our final models on the basis of subsets of the full data set—those individuals with a history of cardiovascular disease (CVD) or stroke (*n* = 214) and those with a history of neither (*n* = 457).

## Results

Descriptive statistics are shown in Table 2. The frequency of the Wahlund ratings indicates that hyperintensities are primarily located, and have greater severity, in the frontal and parieto-occipital regions. As often observed in aging samples, the most commonly reported medical problems were hypertension and high cholesterol.

Results of regression diagnostic tests suggested no evidence of multicollinearity, heteroscedasticity, nonindependence of errors, nonnormality of model residuals, or influential observations (see the online supplemental material). Reaction time variables often display high levels of skew and kurtosis; however, this problem is usually less severe in CRT compared with simple RT. As can be seen in Table 2, in our study *SD* CRT was relatively normally distributed. As such, we did not deem it necessary to rerun models using the log transform of RT, as is often reported in the literature.

Table 3 –8, display the standardized regression coefficients for models including WMH volume, gFA, gMD, Wahlund ratings, individual tract average FA and individual tract average MD, respectively. In order to facilitate comparisons across models, each table contains results from all models with *SD* CRT, *M* CRT, and *CV* CRT as the outcome variables of interest. Given the large number of models and tests in this exploratory study, *p* values are reported for completeness, but we refrain from interpreting individual effects based on these (for the full model results tables, see Tables S2 through S18 in the online supplemental material).[Table-anchor tbl3][Table-anchor tbl4][Table-anchor tbl5][Table-anchor tbl6][Table-anchor tbl7][Table-anchor tbl8]

Across all models, the variance accounted for by age, sex, health covariates and the focal brain imaging variables was small, ranging from 2.6% to 4.8% variance explain for *SD* CRT, with ranges of 3.3% to 5.4% and 0.9% to 3.9% variance explained for *M* CRT and *CV* CRT respectively. As expected given the strong positive correlation between *M* CRT and *SD* CRT, the addition of *M* CRT to models predicting *SD* CRT increased variance explained to between 36.0% and 41.1%. Similarly, and again as expected, when *SD* CRT was added to models predicting *M* CRT, variance explained increased to between 36.6% to 41.7%. (See Tables S2 through S18 in the online supplemental material for full results of model *F*-test variances explained.)

### Covariate Effects

For age and sex, the direction of the effects indicated that female participants and those who were older had higher *SD* CRT, and thus were more variable in performance, whereas men and older participants had higher *M* CRT, indicating they were slower on average across trials. Of the health covariates, all effects sizes were small (absolute β range = .00 to .11), with effects generally indicating that those who have a history of a medical condition were both more variable and have a higher *M* CRT.

### Main Effects

Across models, WMH volume demonstrated consistently larger effects (β range = .05 to .14), with the direction of the effects indicating the increased WMH volume was associated with greater variability and higher average CRT. The largest effects across models were seen with the white matter tract variables. Of the general measures, the largest effect was for gFA predicting *M* CRT (β = 0.15). Considering the specific tract averages, the strongest effects were seen for both FA and MD in the genu of the corpus callosum, particular with *M* CRT.

However, these effects were in the opposite direction to that which might be expected. Positive associations between FA and *SD* CRT (β = 0.14) and *M* CRT (β = 0.17) suggest that higher values of FA are associated with higher average reaction time and greater variability, with opposite effects and interpretation for MD (β = −0.08 and −.022, respectively). Finally, of the models including the Wahlund ratings, the largest effects were seen for ratings in frontal regions (β range = .06 to .15), indicating those with greater severity of lesion in this region were both more variable and had higher *M* CRT.

### Comparison Across *SD* CRT, *M* CRT, and *CV* CRT

Across all models, the largest effects were seen for the coefficients predicting *M* CRT, indicating that the imaging variables were more strongly associated with average performance than variability. However, it is important to note that in absolute terms these effects were still small, and thus the difference in the coefficients predicting *SD* CRT, *M* CRT, and *CV* CRT were also small.

The difference in coefficients from Model 1 to Model 2 for both *SD* CRT and *M* CRT provide information on the degree to which the effects of the imaging variables on *SD* CRT and *M* CRT are attenuated by the inclusion of other CRT summary variable. Across models, the inclusion of either *SD* CRT or *M* CRT resulted in attenuations to the effects of the focal imaging variables. The magnitudes of these attenuations varied. Fractionally larger attenuations were evident in models predicting *SD* CRT when *M* CRT was added as a predictor than the reverse specification. However, these differences were marginal. Many coefficients that were near zero in the original models (Model 1 across tables) showed varied attenuations in magnitude and direction of effect, fluctuating around zero.

### Sensitivity Checks

To assess the sensitivity of our results, we reran all final models using robust regression (see Tables S19 through S21 in the online supplemental material.) The pattern of results was identical to the main models with respect to the direction, magnitude, and associated inferential statistics.

Next, we considered whether those individuals with a history of CVD or stroke drove the observed effects in the total sample. Parameter estimates were compared for the final models in which the sample was split into a sample without history of either CVD or stroke (*n* = 456) and those with a history of either condition (*n* = 214; see Tables S22 through S24 in the online supplemental material).

Again, the pattern of results was largely similar to the main analyses. For each model, for each outcome, effect sizes were generally smaller due. However, the direction of effects and the relative size of effects were broadly consistent. Taken collectively, the results of the sensitivity checks indicated the patterns of results were generally robust of individual influential cases and were not driven by those within the sample with a history of CVD.

## Discussion

In the full sample, WMH volume was positively associated with *SD* CRT; people with larger WMH volume were more variable in CRT responses. Ratings of hyperintensity severity in frontal lobes, but not in other brain areas, were positively associated with *SD* CRT. Neither FA nor MD estimates of general white matter tract integrity were significantly associated with *SD* CRT. Across all models, when *M* CRT was included in the models, an attenuation of effects for white matter variables was observed. Therefore, in the current sample, there were no incremental effects of various metrics of white matter on CRT variability over and above the average effects of CRT speed. Across all models, the variance explained by white matter measures was small (Range = 0.9% to 4.8%).

To investigate the relationships of the imaging measures with different dependent measures derived from the CRT test, we reran our models using both *CV* CRT and *M* CRT as the dependent variable. With *CV* CRT as the dependent variable, the association of the frontal hyperintensity ratings remained, as did ratings of hyperintensities in the parieto-occipital region. However, the direction of these effects differed, specifically in the case of the parieto-occipital association, lower ratings of hyperintensities were associated with greater CRT variability. The counter intuitive direction of this effect suggests this may be a chance finding. It should be noted here that, although frequently used, *CV* CRT is a crude measure of IIV adjusted for mean and a number of issues associated with its use and interpretation have been raised (see, e.g., Hultsch et al., 2008, for discussion).

When models were reestimated using *M* CRT as the dependent variable, associations were found with WMH volume, gFA and gMD. These relationships were found to be independent of *SD* CRT. Given the large number of statistical tests reported in the current study, it is perhaps more informative to consider the magnitude of the standardized effects. The coefficients from the various imaging metrics are larger in magnitude when predicting *M* CRT when compared with *SD* CRT (see Table 3 through Table 8). Only in the case of Wahlund ratings (Table 6) are the standardized effects approximately equal.

Taken together, the results of the present study suggest that various metrics of WM integrity show limited associations with either *SD* CRT and *M* CRT when both variables are included in the models. Put differently, imaging metrics are not incrementally predictive of either *SD* or *M* CRT when the other RT measure is controlled for. This is in contrast to some previous reports suggesting that IIV’s association with WM integrity is largely independent of mean RT speed (Bunce et al., 2007; Fjell et al., 2011; Moy et al., 2011).

The results support findings from earlier work linking white matter integrity and information processing speed in the current sample. For example, Valdés Hernández et al. (2013) showed that WMH load is associated with both general cognitive ability and with information processing speed in old age. Using diffusion tensor MRI indicators of white matter tract integrity rather than hyperintensities, Penke et al. (2012) showed that the association between the integrity of white matter tracts and general cognitive ability was fully mediated by the speed of basic information processing. However, neither of the previous studies in the current sample have considered CRT variability.

As outlined in the introductory paragraphs, there remain some differences in the methodologies applied across studies—for example, the specific WMH measures, composition of the samples, and different RT tests—which make drawing meaningful direct comparisons, difficult. More importantly, many of the previous studies were underpowered. Comparing our results to those studies with comparable sample sizes, for example Haynes et al. (2017; see Table 1), similar patterns of results are seen. Specifically, the effects of interest are largely small, and when models are estimated, including both metrics of average RT and variability in RT, effects are attenuated and drop below nominal alpha thresholds.

Our study is one of the largest-sampled studies to consider *SD* CRT, *M* CRT, and *CV* CRT alongside multiple different metrics brain white matter. Though exploratory in nature, such an approach can be advantageous in an area where current findings contain inconsistencies. For example, our study is one of few large studies to address the issue of whether the association between frontal WMH and CRT IIV might be explained by the shared association of these two variables with mean RT. The current study does not resolve the question of whether variability or average RT performance is more strongly related to fundamental measures of the brain. However, we hope that others will follow in adding further evidence from adequately powered studies to help resolve the existing inconsistencies in the results. Such studies would benefit from both more extensive and detailed proxy behavioral measures of speed, and more biologically derived metrics of brain activation. Some investigations of this type have been carried out and they provide interesting insights. For example, studies of blood oxygen-level-dependent signal variability in the brain suggested that, at least within the brain itself, IIV and mean signal are distinct quantities (Garrett, Kovacevic, McIntosh, & Grady, 2011).

Moreover, contrary to the general consensus regarding RT IIV, it appears that as far as brain signals are concerned, greater - not smaller - variability may be advantageous, representing greater adaptability from moment to moment (Garrett et al., 2011, 2013; McIntosh, Kovacevic, & Itier, 2008). This underlines the complexity of the topic and warrants further research into more nuanced aspects of variability.

The effects noted in the current study are generally small. However, the current sample is sufficiently large and the measures sufficiently reliable that we judge that these effect sizes are unlikely to have been much biased on the basis of features of the study, and we judge that the estimates presented here are robust. Of course, the practical question of the importance of such small effects for an individual’s daily functioning remains; however, small effects can have importance at the population level.

The analyses also suggested a number of covariates to be significant predictors of the various measures of CRT (*SD* CRT, *M* CRT, and *CV* CRT). Specifically, in a majority of models, sex was a significant covariate with the direction of the effect suggesting that women were more variable in performance than men. Interestingly, given the narrow age range of the current sample (birth cohort), age had a significant effect on CRT measures. In the case of predicting *SD* CRT this was true only in the models without *M* CRT. In predicting *M* CRT, age remained a significant covariate after the inclusion of *SD* CRT. Collectively, this pattern of results suggests that in the current sample, at least, age is primarily associated with overall *M* CRT and not variability. Although it may seem surprising given the narrow range of age, and thus low variance, to see significant effects, speeded performance has been shown to be sensitive to the effects of aging. On a practical note, the significant effects also validate the inclusion of age as a covariate in our models.

This study provided a systematic exploratory investigation of the association between white matter integrity and RT IIV in a large sample of older adults. Sample sizes are often small in neuropsychological studies (but see Anstey et al., 2007; Bunce et al., 2007; Haynes et al., 2017, for notable exceptions), which can exacerbate the problem of unreliable, unreproducible results. Greater number of large, adequately powered studies are needed to clarify the effects. Another strength of the present study is the use of multiple measures of white matter integrity, providing a comprehensive look at the associations with RT IIV and speed. It should be noted that we considered only the volume and location of WMHs, and white matter tract integrity and that it is known that as the brain ages and in the presence of increasing degrees of WMHs, the integrity of normal appearing white matter becomes increasingly nonnormal. Future studies should include measures of the tissue integrity of the whole brain, which may provide some additional insight into the associations between integrity and the average and variability in speeded test performance.

The RT measures used in the present study were calculated from 40 trials, which is a modest number by comparison with some other studies investigating RT variability in relation to white matter integrity. Even though 20 trials have previously been shown to be an adequate number for this type of investigation (Bunce et al., 2013), we know that the reliability of both IIV and mean RT increases, and perhaps more importantly, the discrepancy in reliabilities between the two measures decreases, with the larger number of trials (Schmiedek, Lövdén, & Lindenberger, 2009). Therefore, having more trials from which mean RT and IIV are calculated is highly encouraged in future studies in this field.

In conclusion, we found little evidence that white matter integrity explains variance in *SD* CRT over that explained by the *M* CRT. The results suggest that the association between WMH load and CRT variability might be secondary to the association of WMH with average CRT. At least in the case of CRT and in the cross-sectional analysis of the current sample, neither WMH load nor white matter tract integrity appear to be strong candidates to explain age differences in CRT IIV. Further investigations into putative causes of increased CRT in older adults are required.

## Supplementary Material

10.1037/neu0000483.supp

## Figures and Tables

**Table 1 tbl1:** Literature Summary

Author	Sample characteristics	Reaction time (RT) task	Reaction time and white matter measures and covariates	Summary of results
Brain tissue volume/area				
Anstey et al. (2007)	*N* = 489 (Healthy adults [HA]: *n* = 432, 209 female; Adults with mild cognitive disorder [MCD]: *n* = 57, 19 female) Age (HA: *M* = 62.6, range = 60–64; MCD: *M* = 62.5)	Simple RT (to light) and two-way CRT (right vs. left to light location). Four blocks of 20 trials for simple RT and two blocks of 20 trials for CRT	Mean independent variable for simple RT (MIVS) and mean independent variable for CRT (MIVC) Corpus callosum (CC) area Age, sex, education, alcohol intake, forced expiratory volume/height, smoking, vision, grip strength	HA: *r*_(MIVS, CC area)_ = −.11; *r*_(MIVC, CC area)_, *ns* (with covariates controlled and mean RT controlled, *ns*) MCD: *r*_(MIVS, CC area)_ =−.32; *r*_(MIVC, CC area)_ *ns*. MIVS but not MIVC explained variance in CC area that was over that explained by the covariates and mean RT
Lövdén et al. (2013)	*N* = 43 (*n* = 25 younger adults [13 female]; *n* = 18 older adults [nine female]) Age (younger: *M* = 25.0, range = 20–31; older: *M* = 70.1, range = 65–80)	Two-way CRT (odd vs. even number discrimination) with individually determined stimulus presentation time. Two blocks of 40 trials for up to 101 days; 20 trials with longest presentation time selected from each block	Day-to-day, block-to-block, and trial-to-trial variability partitioned using multilevel linear model with three levels Regional brain volumes (regions of interest [ROIs]: dorsolateral prefrontal cortex, orbital frontal cortex, the adjacent prefrontal white matter, primary visual cortex, the hippocampus, the caudate nucleus, and the cerebellar hemispheres) Age	Day-to-day or trial-to-trial variability not associated with any regional volume; higher block-to-block variability associated with lower frontal white matter volumes, with and without controlling for mean RT
Jackson, Balota, Duchek, and Head (2012)	*N* = 166 (HA: *n* = 133 [87 female]; early stage Alzheimer’s disease: *n* = 33 [17 female]) Age (HA: *M* = 68.0, range = 46–96; early stage Alzheimer’s disease: *M* =76.6, range = 61–88)	Stroop (104 trials), Simon (120 trials), and switching tasks (consonant vs. vowel or odd vs. even number; 60 trials). Stroop and Simon tasks included congruent, incongruent, and neutral trials	Coefficient of variability (*CV*) and ex-Gaussian parameters calculated as composites on the basis of the performance on the three tasks Regional brain volumes (ROIs: total cerebral white matter, superior frontal gyrus, ventra/dorsal-lateral prefrontal cortex, anterior cingulate, posterior cingulate, precuneus, inferior parietal lobule; primary visual cortex as a control Education, sex, interval between the scan and cognitive assessment, scanner type, cardiovascular health, depression, age, dementia status	Smaller *CV* associated with larger volumes of: total cerebral white matter, posterior cingulate, precuneus, ventra/dorsal-lateral prefrontal cortex, and superior frontal gyrus. Ex-Gaussian parameters: no main effects for sigma; smaller tau associated with larger volumes of total cerebral white matter, posterior cingulate, precuneus, ventra/dorsal-lateral prefrontal cortex, superior frontal gyrus
Walhovd and Fjell (2007)	*N* = 71 (40 female) Age (*M* = 52.1, range = 20–88)	Three-stimuli oddball task; 210 trials, including 21 targets, 21 distractors, and 168 standard stimuli; response required to the targets only	Intraindividual variability (IID) and IID with mean RT regressed out Total white matter volume Age, sex	IID: *r* = −.30 WM volume; *r* = −.29 after controlling for mean RT
White matter hyperintensities (WMH)				
Bunce et al. (2007)	*N* = 469 (48% female): Same sample as Anstey et al. (2007) Age (*M* = 62.6, range = 60–64)	Simple RT (response to a light stimulus) and two-way CRT (right vs. left in response to a light location). Four blocks of 20 trials for simple RT and two blocks of 20 trials for CRT	Mean absolute residual for simple RT (MARS) and mean absolute residual for CRT (MARC; adjusted for practice effects but not mean) and MIVS and MIVC (adjusted for practice and mean simple RT or CRT) WMH in ROIs: frontal, parietal, temporal, and occipital white matter, anterior cap, posterior cap, and periventricular body Sex, depression, education	Frontal region: *r*_(WMH, MARC*)*_ = .16; *r*_(WMH, MIVC)_ = .14; no significant WMH-MARS or WMH-MIVS in any ROI. Betas from regression analysis, including covariates and a linear and quadratic term for RT: MARC = .15; MIVC = .13; no significant associations between WMH and MARS or MIVS
Haynes et al. (2017)	*N* = 526 (54% female) Age (*M* = 78.38, range = 70–90)	Simple RT (response to a square stimulus) and CRT (same-different color judgement). Two assessments were performed for each test, with a total of 36 trials for simple RT and 40 trials for CRT.	IID (calculated from residuals after partialing out the effects of age, trial number and time-on-task and their interactions) WMH: Total volume; periventricular and deep white matter (the latter also subdivided into frontal, parietal, temporal, and occipital lobes) Age, education, sex	No significant associations for simple RT in any region considered CRT IID and mean CRT were both related to frontal WMH volume, but not when entered in the model simultaneously
Diffusion tensor imaging measures				
Fjell, Westlye, Amlien, and Walhovd (2011)	*N* = 270 (57% female) Age (*M* = 48.6, range = 20–83)	Adapted flanker task (a target arrow surrounded by irrelevant distracter arrows, either congruent or incongruent to the target). On-task training—a message to respond faster if 10% over own mean RT; 416 trials, with a break half way through	RT IID FA, MD, RD, and AD Age, sex, and median RT	Congruent trials: RT IID negatively associated with FA and positively associated with MD, RD, and AD. Associations were widespread across the brain (25% to 50% of the skeleton voxels). The relationship between RT IID and diffusion tensor imaging measures stronger in the older than in the younger part of the sample Incongruent trials: RT IID negatively associated with FA and positively associated with the other diffusion parameters; effect less spatially widespread (<25% of voxels)
Moy et al. (2011)	*N* = 61 (40% to 71% female, depending on age group) Four age groups (1. *M* = 27.8, range = 20–34; 2. *M* = 41.9, range = 35–50; 3. *M* = 57.8, range = 51–65; 4. *M* = 74.3, range = >66)	Simple RT (response to a cross appearing of the screen); 120 trials	RT IID (calculated from residuals after controlling for trial, age and their interaction) FA, MD, RD, and AD Education, gender, age	FA related to IIV in left and right part of the splenium, and in posterior, middle and anterior parts of the left inferior fronto-occipital fasciculus. Without controlling for age, significant effects found for RD and MD as well as FA
Deary et al. (2006)	*N* = 40 (19 female) Age (*M* = 83)	Simple RT and four-way CRT to numbers; 20 trials for simple RT, 40 trials for CRT	RT IID FA, MD, and magnetization transfer ratio	Centrum semiovale *r*_(simple RT IID, FA)_ = −.52; frontal white matter *r*_(CRT IID, FA)_ = −.33 (deemed not significant given the number of comparisons: 14 for FA in frontal region alone). No associations of RT IID with MD or magnetization transfer ratio
Mella, de Ribaupierre, Eagleson, and de Ribaupierre (2013)	*N* = 25 12 younger (10 female) 13 older (10 female) Age (younger: *M* = 21.69, range = 18–30; older: *M* = 69.9, range = 61–82)	Two-way CRT from a cross-square task; left vs. right response to indicate the stimulus (cross changing into square) location. The task was administered in the scanner during an fMRI scan (eight blocks of 12 trials) and outside the scanner (five blocks of 24 trials)	RT IID calculated after regressing out the effects of age group, item number, block number and their interactions FA, MD, RD, and AD Age group regressed out, together with practice effects prior to calculating RT IID	Outside the scanner: In forceps minor *r*_(RT IID, FA)_= −.50 (the only region out of 20 with a significant association); *r*(RT IID, MD/RD) ranging between .41 and .55 Inside the scanner: None of the above relationships replicated
*Note*. CRT = choice reaction time; FA = fractional anisotropy; MD = mean diffusivity; RD = radial diffusivity; AD = axial diffusivity.				

**Table 2 tbl2:** Sample Demographics and Descriptive Statistics for Variables

Variable	*N*	*M*	*SD*	Skew	Kurtosis
Reaction time measures					
*M* CRT (m/s)	670	645.15	86.30	.76	1.11
*SD* CRT (m/s)	670	138.99	36.88	.87	1.09
Coefficient of variability CRT	670	.21	.04	.72	.97
Quantitative imaging					
White matter hyperintensity volume (cm^3^)	671	12.08	12.84	2.26	7.88
White matter hyperintensity residual	671	.00	1.00	2.29	8.07
WMT gFA	649	−.01	.91	.36	.27
WMT gMD	649	−.01	.92	.23	−.14
Tractography					
Genu corpus callosum (FA)	628	.41	.05	−.08	−.15
Splenium corpus callosum (FA)	645	.49	.07	−.36	.63
Arcuate fasciculus (FA)	547	.44	.04	−.33	.61
Anterior thalamic radiation (FA)	531	.32	.03	−.15	.21
Rostral cingulum (FA)	612	.41	.04	−.39	.49
Uncinate fasciculus (FA)	535	.33	.03	−.15	.03
Inferior longitudinal thalamic radiation (FA)	643	.39	.04	−.23	−.06
Genu corpus callosum (MD)	628	769.45	66.08	.41	1.02
Splenium corpus callosum (MD)	645	977.31	174.66	.77	1.21
Arcuate fasciculus (MD)	547	652.53	45.80	1.24	4.48
Anterior thalamic radiation (MD)	531	755.19	55.96	.39	−.03
Rostral cingulum (MD)	612	649.51	40.18	.37	.66
Uncinate fasciculus (MD)	535	762.01	46.65	.19	.05
Inferior longitudinal thalamic radiation (MD)	643	771.78	84.24	1.51	3.56
Age (years)	671	72.49	.71	.00	−.86
		0	1	2	3
Wahlund rating					
Frontal	671	10	482	145	34
Parieto-occipital	671	36	446	150	39
Temporal	671	577	86	8	0
Infrattentorial	671	576	81	13	1
Basal ganglia	671	559	88	23	1
		*n*	*%*		
Sex	671				
Male		356	53.1%		
Female		315	46.9%		
		Yes (*n*)	%	No (*n*)	%
Health covariate					
Blood pressure	671	330	49.2%	341	50.8%
Diabetes	671	69	10.3%	602	89.7%
Cholesterol	671	283	42.2%	388	57.8%
Cardiovascular disease	671	182	27.1%	489	72.9%
Blood circulation	671	114	17.0%	557	83.0%
Stroke	671	46	6.9%	625	93.1%
*Note*. Both white matter tract integrity general fractional anisotropy factor (WMT gFA) and white matter tract integrity general mean diffusivity factor (WMT gMD) are regression-based factor scores, standardized to mean of 0 and standard deviation 1. CRT = choice reaction time; FA = fractional anisotropy; MD = mean diffusivity.					

**Table 3 tbl3:** Standardized Beta Coefficients for Models 1 and 2 Predicting SD CRT, M CRT and CV CRT from WM Hyperintensity Volume (n = 670)

	*SD* CRT				*M* CRT				*CV* CRT	
	Model 1		Model 2		Model 1		Model 2		Model 1	
Predictors	β	*p*	β	*p*	β	*p*	β	*p*	β	*p*
Sex	−.117	.003	−.143	<.001	.043	.266	.115	<.001	−.176	<.001
Age (years)	.076	.048	−.001	.969	.125	.001	.077	.011	.010	.798
High blood pressure	−.011	.790	.000	.994	−.018	.660	−.011	.726	−.005	.901
Diabetes	.031	.422	−.015	.627	.075	.056	.055	.072	−.010	.797
High cholesterol	.035	.392	−.001	.979	.058	.158	.036	.261	.001	.973
Cardiovascular disease	.079	.047	.062	.048	.028	.480	−.021	.502	.086	.031
Blood circulation	.077	.044	.052	.084	.040	.290	−.007	.809	.071	.063
History of stroke	.055	.160	.046	.131	.014	.723	−.020	.512	.058	.134
WM hyperintensity volume	.106	.006	.019	.536	.140	<.001	.074	.015	.047	.224
*M* CRT			.620	<.001						
*SD* CRT							.619	<.001		
*Note*. CRT = choice reaction time; *CV* = coefficient of variability; WM = white matter.										

**Table 4 tbl4:** Standardized Beta Coefficients for Models 1 and 2 Predicting SD CRT, M CRT, and CV CRT from General Fractional Anisotropy (gFA; n = 647)

	*SD* CRT				*M* CRT				*CV* CRT	
	Model 1		Model 2		Model 1		Model 2		Model 1	
Predictors	β	*p*	β	*p*	β	*p*	β	*p*	β	*p*
Sex	−.114	.004	−.142	<.001	.044	.264	.115	<.001	−.173	<.001
Age (years)	.087	.026	.004	.902	.133	.001	.079	.010	.019	.626
High blood pressure	−.012	.775	.003	.935	−.023	.574	−.016	.624	−.004	.930
Diabetes	.033	.415	−.016	.617	.077	.053	.057	.068	−.011	.793
High cholesterol	.043	.313	−.002	.948	.072	.089	.045	.170	.004	.922
Cardiovascular disease	.071	.079	.068	.034	.006	.883	−.038	.229	.090	.027
Blood circulation	.076	.051	.057	.060	.030	.441	−.017	.571	.078	.046
History of stroke	.061	.127	.051	.101	.015	.698	−.022	.474	.065	.104
gFA	.056	.150	−.039	.212	.152	<.001	.117	<.001	−.017	.658
*M* CRT			.627	<.001						
*SD* CRT							.618	<.001		
*Note*. CRT = choice reaction time; *CV* = coefficient of variability.										

**Table 5 tbl5:** Standardized Beta Coefficients for Models 1 and 2 Predicting SD CRT, M CRT, and CV CRT from General Mean Diffusivity (gMD; n = 647)

	*SD* CRT				*M* CRT				*CV* CRT	
	Model 1		Model 2		Model 1		Model 2		Model 1	
Predictors	β	*p*	β	*p*	β	*p*	β	*p*	β	*p*
Sex	−.113	.005	−.143	<.001	.047	.238	.118	<.001	−.173	<.001
Age (years)	.089	.024	.005	.874	.135	.001	.080	.010	.019	.624
High blood pressure	−.006	.890	−.001	.971	−.007	.860	−.004	.908	−.005	.896
Diabetes	.032	.434	−.014	.656	.073	.070	.053	.090	−.010	.805
High cholesterol	.040	.344	.000	.990	.065	.125	.040	.228	.005	.909
Cardiovascular disease	.073	.071	.066	.038	.012	.775	−.034	.284	.089	.028
Blood circulation	.076	.051	.058	.060	.030	.446	−.018	.562	.078	.046
History of stroke	.063	.112	.050	.110	.022	.587	−.018	.568	.064	.107
gMD	.022	.577	−.031	.317	.084	.031	.071	.021	−.013	.736
*M* CRT			.623	<.001						
*SD* CRT							.623	<.001		
*Note*. CRT = choice reaction time; *CV* = coefficient of variability.										

**Table 6 tbl6:** Standardized Beta Coefficients for Models 1 and 2 Predicting SD CRT, M CRT, and CV CRT From Wahlund Ratings (n = 670)

	*SD* CRT				*M* CRT				*CV* CRT	
	Model 1		Model 2		Model 1		Model 2		Model 1	
Predictors	β	*p*	β	*p*	β	*p*	β	*p*	β	*p*
Sex	−.117	.003	−.144	<.001	.042	.274	.115	<.001	−.176	<.001
Age (years)	.085	.026	.002	.960	.135	<.001	.082	.006	.015	.689
High blood pressure	−.003	.940	.006	.846	−.015	.712	−.013	.682	.003	.932
Diabetes	.025	.525	−.019	.533	.071	.068	.056	.069	−.017	.669
High cholesterol	.031	.448	.000	.992	.051	.215	.032	.327	.001	.977
Cardiovascular disease	.087	.029	.066	.035	.033	.403	−.020	.517	.092	.021
Blood circulation	.079	.038	.054	.074	.041	.283	−.008	.793	.075	.053
History of stroke	.059	.135	.046	.137	.020	.601	−.016	.611	.060	.129
Wahlund frontal	.149	.002	.058	.126	.147	.002	.055	.145	.099	.040
Wahlund parieto-occipital	−.063	.183	−.073	.049	.016	.728	.055	.137	−.098	.039
Wahlund temporal	.024	.543	−.008	.794	.053	.188	.038	.231	.002	.964
Wahlund infrattentorial	.062	.117	.024	.435	.061	.123	.023	.467	.041	.309
Wahlund basal ganglia	−.060	.152	−.015	.653	−.073	.081	−.036	.275	−.031	.463
*M* CRT			.620	<.001						
*SD* CRT							.616	<.001		
*Note*. CRT = choice reaction time; *CV* = coefficient of variability.										

**Table 7 tbl7:** Standardized Beta Coefficients for Models 1 and 2 Predicting SD CRT, M CRT, and CV CRT From Tract Average FA (n = 358)

	*SD* CRT				*M* CRT				*CV* CRT	
	Model 1		Model 2		Model 1		Model 2		Model 1	
Predictors	β	*p*	β	*p*	β	*p*	β	*p*	β	*p*
Sex	−.076	.188	−.097	.039	.035	.546	.080	.089	−.116	.046
Age (years)	.069	.209	.004	.933	.111	.044	.070	.116	.012	.821
High blood pressure	−.013	.824	.016	.733	−.049	.398	−.041	.377	.017	.767
Diabetes	.000	1.00	−.015	.746	.025	.657	.025	.583	−.01	.862
High cholesterol	.036	.531	−.001	.983	.062	.276	.041	.373	.000	.998
Cardiovascular disease	.047	.404	.030	.511	.029	.607	.001	.978	.034	.553
Blood circulation	.109	.047	.092	.037	.028	.612	−.036	.411	.111	.044
History of stroke	.063	.244	.039	.377	.041	.445	.004	.926	.052	.340
Genu corpus callosum	.144	.027	.043	.422	.172	.008	.087	.099	.061	.349
Splenium corpus callosum	.026	.637	.064	.155	−.064	.248	−.080	.077	.072	.198
Arcuate fasciculus	.017	.799	.028	.608	−.019	.785	−.029	.601	.049	.476
Anterior thalamic radiation	−.113	.097	−.031	.576	−.139	.041	−.072	.190	−.052	.447
Rostral cingulum	−.098	.148	−.054	.320	−.073	.278	−.016	.775	−.084	.217
Uncinate fasciculus	.015	.834	.035	.544	−.034	.633	−.043	.457	.040	.573
Inferior longitudinal thalamic radiation	.015	.817	.026	.631	−.018	.791	−.027	.619	.016	.812
*M* CRT			.591	<.001						
*SD* CRT							.589	<.001		
*Note*. CRT = choice reaction time; *CV* = coefficient of variability; FA = fractional anisotropy.										

**Table 8 tbl8:** Standardized Beta Coefficients for Models 1 and 2 Predicting SD CRT, M CRT, and CV CRT From Tract Average MD (n = 358)

	*SD* CRT				*M* CRT				*CV* CRT	
	Model 1		Model 2		Model 1		Model 2		Model 1	
Predictors	β	*p*	β	*p*	β	*p*	β	*p*	β	*p*
Sex	−.103	.083	−.125	.010	.037	.530	.097	.043	−.152	.012
Age (years)	.081	.148	.008	.861	.123	.027	.076	.092	.021	.708
High blood pressure	−.014	.803	.011	.820	−.042	.460	−.034	.465	.012	.842
Diabetes	.008	.890	−.013	.781	.034	.536	.030	.507	−.006	.918
High cholesterol	.032	.573	−.001	.976	.056	.317	.038	.409	−.001	.981
Cardiovascular disease	.050	.381	.024	.599	.043	.443	.014	.756	.031	.588
Blood circulation	.102	.062	.089	.044	.021	.692	−.038	.388	.106	.054
History of stroke	.072	.180	.038	.389	.058	.274	.016	.706	.053	.330
Genu corpus callosum	−.082	.236	.047	.404	−.218	.002	−.170	.002	.031	.655
Splenium corpus callosum	−.025	.653	−.037	.404	.021	.698	.035	.422	−.040	.472
Arcuate fasciculus	.038	.608	.005	.935	.056	.447	.034	.571	.014	.849
Anterior thalamic radiation	.172	.018	.031	.604	.238	.001	.138	.019	.073	.316
Rostral cingulum	−.077	.296	−.049	.408	−.046	.524	−.002	.977	−.064	.385
Uncinate fasciculus	.008	.914	−.028	.638	.061	.405	.056	.342	−.021	.777
Inferior longitudinal thalamic radiation	−.052	.413	−.037	.466	−.024	.697	.006	.910	−.049	.441
*M* CRT			.594	<.001						
*SD* CRT							.581	<.001		
*Note*. CRT = choice reaction time; *CV* = coefficient of variability; MD = mean diffusivity.										
