# Supplementary material for: Reaction Time Variability and Brain White Matter Integrity
Source: Neuropsychology. 2019 Jul;33(5):642–57. doi: 10.1037/neu0000483 (PMC6683973; doi:10.1037/neu0000483)
Supplement: Supplementary file 1 [file R+R4_SpeedVar_Supplement_neu0000483.doc]

**Online Supplementary Material**

**Reaction Time Variability and Brain White Matter Integrity**

**Booth et al.**

Table S1: Bivariate correlations of age with RT and white matter variables.

|  | ***r* (2.dp)** |
| --- | --- |
| ***Reaction Time Measures*** |  |
| CRT Mean | 0.15 |
| CRT Standard Deviation | 0.11 |
|  |  |
| ***Quantitative Imaging*** |  |
| Intracranial Volume (cm^3^) | -0.06 |
| WM Hyperintensity Vol resid. (cm^3^) | 0.15 |
| WMT gFA | 0.09 |
| WMT gMD | 0.13 |
|  |  |
| ***Wahlund Rating*** |  |
| Frontal | 0.05 |
| Parieto-Occipital | 0.05 |
| Temporal | -0.01 |
| Infrattentorial | -0.01 |
| Basal Ganglia | -0.02 |
|  |  |
| ***Tract Averages FA*** |  |
| Genu Corpus Callosum | -0.02 |
| Splenium Corpus Callosum | 0.04 |
| Arcuate Fasciculus | -0.05 |
| Anterior Thalamic Radiation | -0.08 |
| Rostral Cingulum | -0.10 |
| Uncinate Fasciculus | -0.07 |
| Inferior Longitudinal Thalamic Radiation | -0.12 |
|  |  |
| ***Tract Averages MD*** |  |
| Genu Corpus Callosum | 0.11 |
| Splenium Corpus Callosum | -0.07 |
| Arcuate Fasciculus | 0.07 |
| Anterior Thalamic Radiation | 0.14 |
| Rostral Cingulum | 0.13 |
| Uncinate Fasciculus | 0.05 |
| Inferior Longitudinal Thalamic Radiation | 0.11 |

**Individual linear model results**

**Note: There is a degree of redundancy in the assumption tests. Included for completeness*

**CRT SD**

Table S2: Regression Model Results for Four-Choice Reaction Time SD and WMH Volume (n=670)

|  |  | **Model 1** |  |  | **Model 2** |  |
| --- | --- | --- | --- | --- | --- | --- |
|  | **b** | ***se*** | ***p*-value** | **b** | ***se*** | ***p*-value** |
| Age | 3.952 | 1.998 | 0.048 | -0.061 | 1.582 | 0.969 |
| Sex | -8.607 | 2.861 | 0.003 | -10.580 | 2.250 | <.001 |
|  |  |  |  |  |  |  |
| Hypertension | -0.792 | 2.975 | 0.790 | 0.019 | 2.338 | 0.994 |
| Diabetes | 3.813 | 4.742 | 0.422 | -1.817 | 3.736 | 0.627 |
| Cholesterol | 2.634 | 3.078 | 0.392 | -0.062 | 2.422 | 0.979 |
| CVD | 6.539 | 3.279 | 0.047 | 5.104 | 2.577 | 0.048 |
| Blood Circulation | 7.534 | 3.739 | 0.044 | 5.083 | 2.940 | 0.084 |
| Stroke | 7.944 | 5.650 | 0.160 | 6.705 | 4.440 | 0.131 |
|  |  |  |  |  |  |  |
| WMH Volume | 3.895 | 1.426 | 0.006 | 0.700 | 1.131 | 0.536 |
|  |  |  |  |  |  |  |
| CRT Mean | - | - | - | 0.265 | 0.013 | <.001 |
| F | 4.48 | (9,660) | <.001 | 47.56 | (10,659) | <.001 |
| R-square | 0.058 |  |  | 0.419 |  |  |
| Adjusted R-square | 0.045 |  |  | 0.410 |  |  |

*Notes:* WMH = white matter hyperintensity; CVD = cardiovascular disease; CRT = choice reaction time.

***Assumptions***

Max VIF = 1.2.


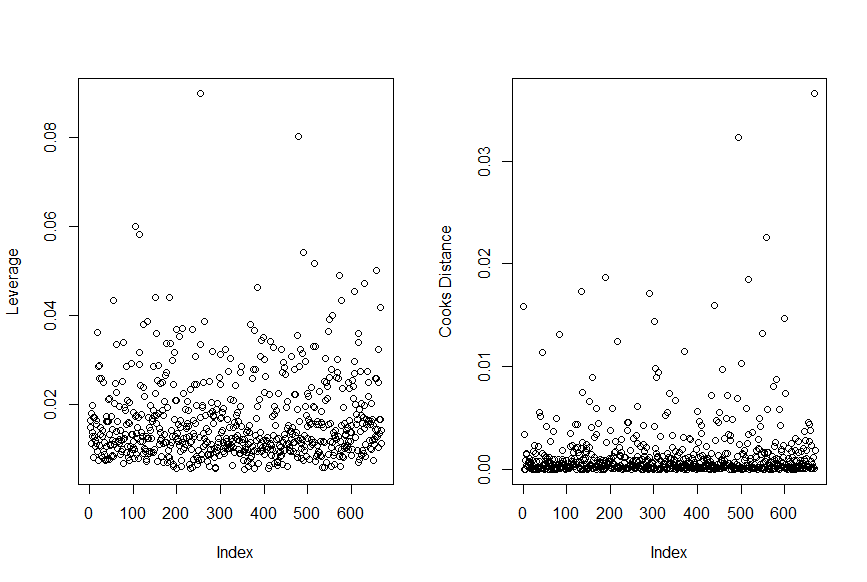


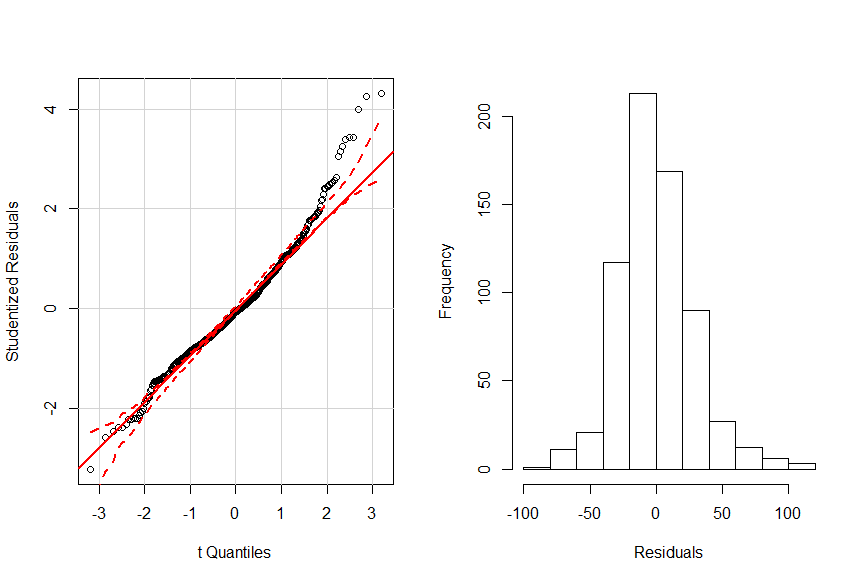


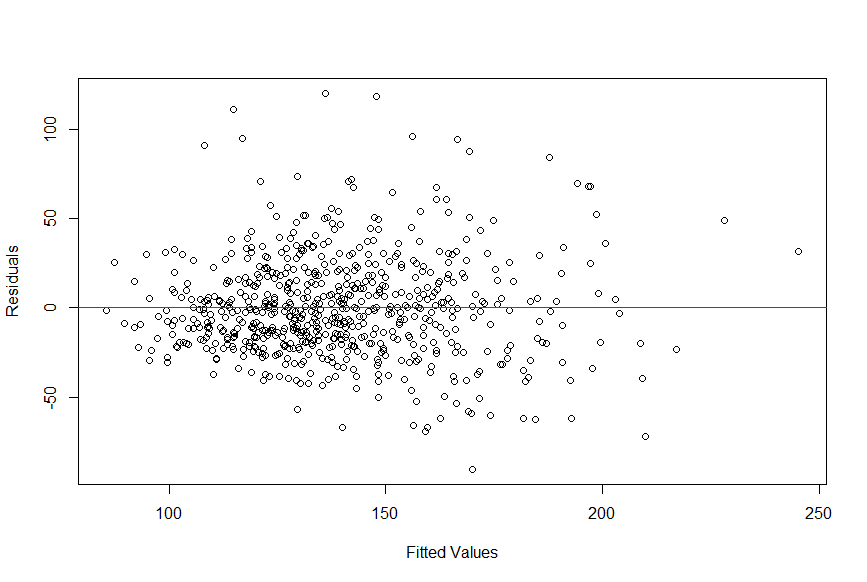


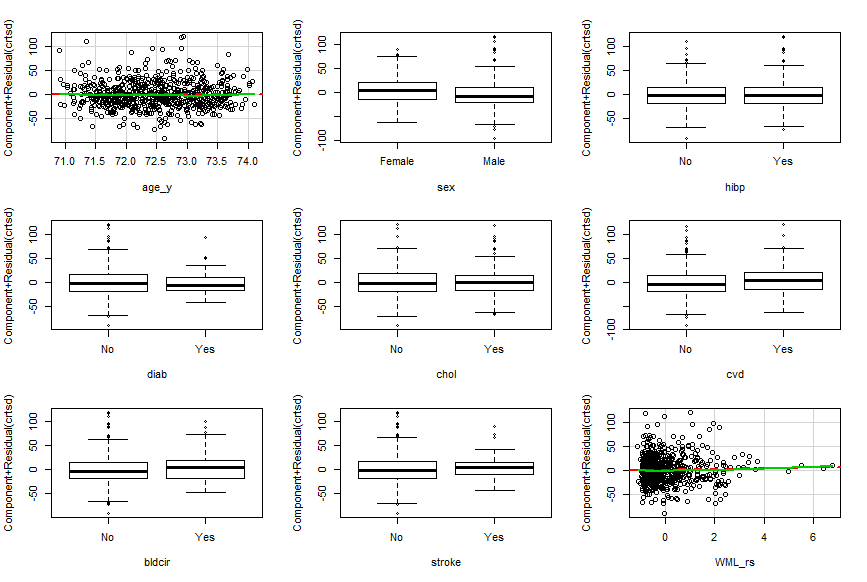


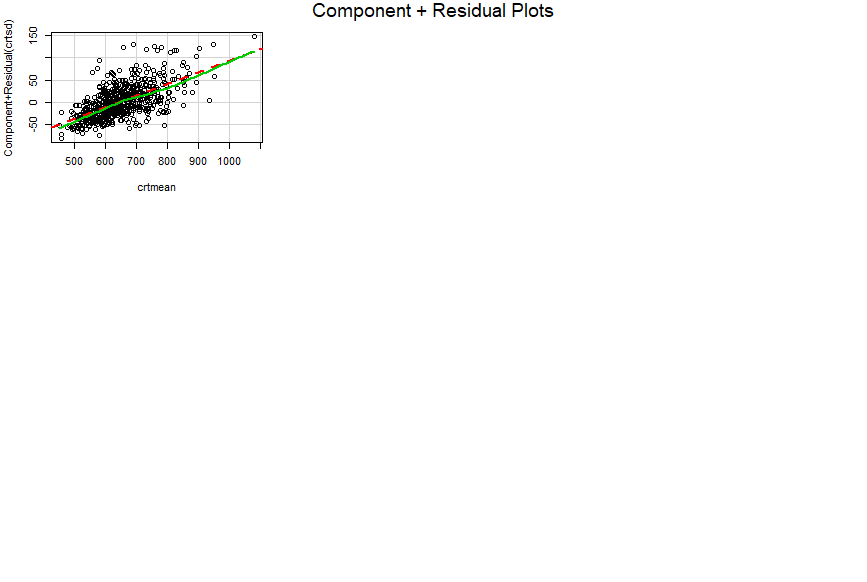


Table S3: Regression Model Results for Four-Choice Reaction Time SD and WMH Severity in different brain regions (n=670)

|  |  | **Model 1** |  |  | **Model 2** |  |
| --- | --- | --- | --- | --- | --- | --- |
|  | **b** | ***se*** | ***p*-value** | **b** | ***se*** | ***p*-value** |
| Age | 4.425 | 1.982 | 0.026 | 0.079 | 1.574 | 0.960 |
| Sex | -8.661 | 2.862 | 0.003 | -10.599 | 2.254 | <.001 |
|  |  |  |  |  |  |  |
| Hypertension | -0.226 | 2.980 | 0.940 | 0.455 | 2.345 | 0.846 |
| Diabetes | 3.027 | 4.757 | 0.525 | -2.343 | 3.753 | 0.533 |
| Cholesterol | 2.340 | 3.084 | 0.448 | -0.024 | 2.429 | 0.992 |
| CVD | 7.173 | 3.284 | 0.029 | 5.474 | 2.586 | 0.035 |
| Blood Circulation | 7.790 | 3.754 | 0.038 | 5.296 | 2.956 | 0.074 |
| Stroke | 8.554 | 5.718 | 0.135 | 6.703 | 4.500 | 0.137 |
|  |  |  |  |  |  |  |
| Wahlund: Frontal | 12.421 | 3.981 | 0.002 | 4.831 | 3.155 | 0.126 |
| Wahlund: Parieto-Occipital | -5.126 | 3.843 | 0.183 | -5.952 | 3.024 | 0.049 |
| Wahlund: Basal Ganglia | -11.894 | 8.293 | 0.152 | -2.938 | 6.540 | 0.653 |
| Wahlund: Temporal | 8.293 | 13.619 | 0.543 | -2.807 | 10.73 | 0.794 |
| Wahlund: Infratentorial | 16.061 | 10.225 | 0.117 | 6.298 | 8.060 | 0.435 |
|  |  |  |  |  |  |  |
| CRT Mean | - | - | - | 0.265 | 0.013 | <.001 |
| F | 3.609 | (13, 656) | <.001 | 34.32 | (14,655) | <.001 |
| R-square | 0.067 |  |  | 0.423 |  |  |
| Adjusted R-square | 0.048 |  |  | 0.411 |  |  |

*Notes:* CVD = cardiovascular disease; CRT = choice reaction time.

***Assumptions***

Max VIF = 1.63


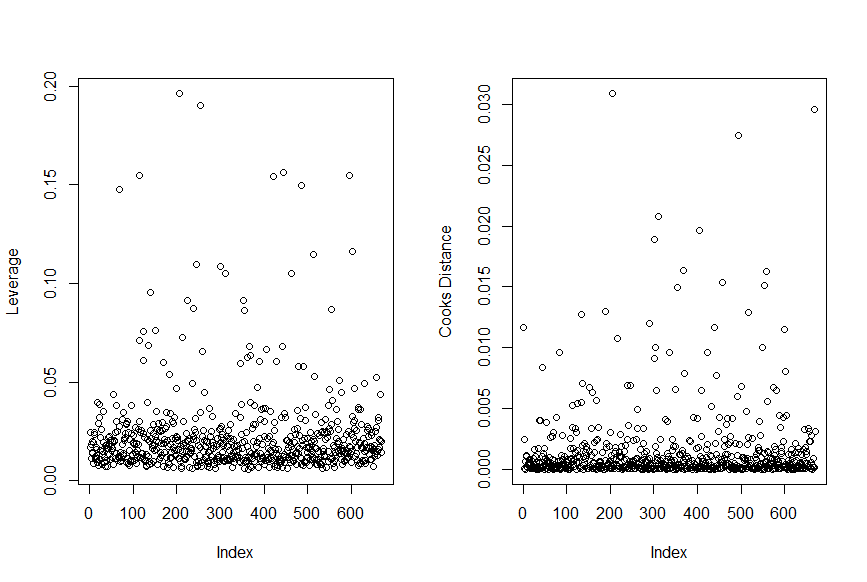


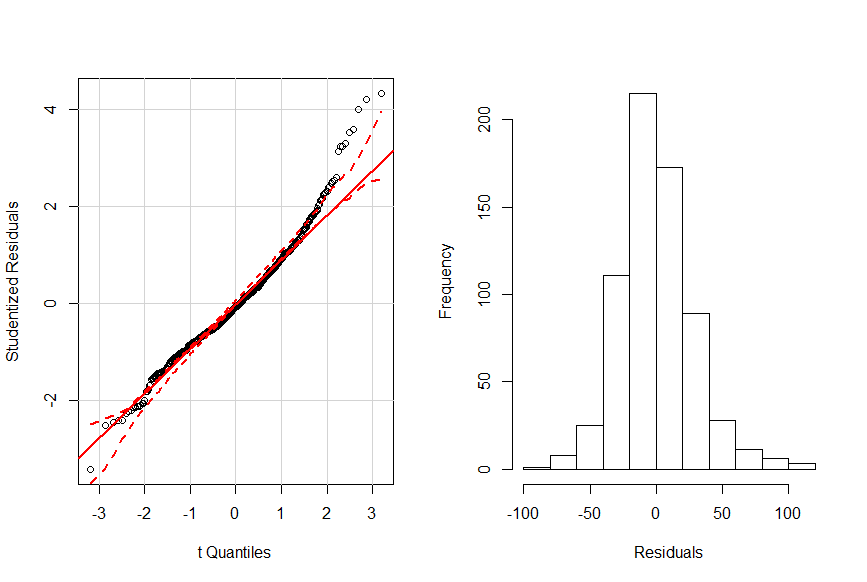


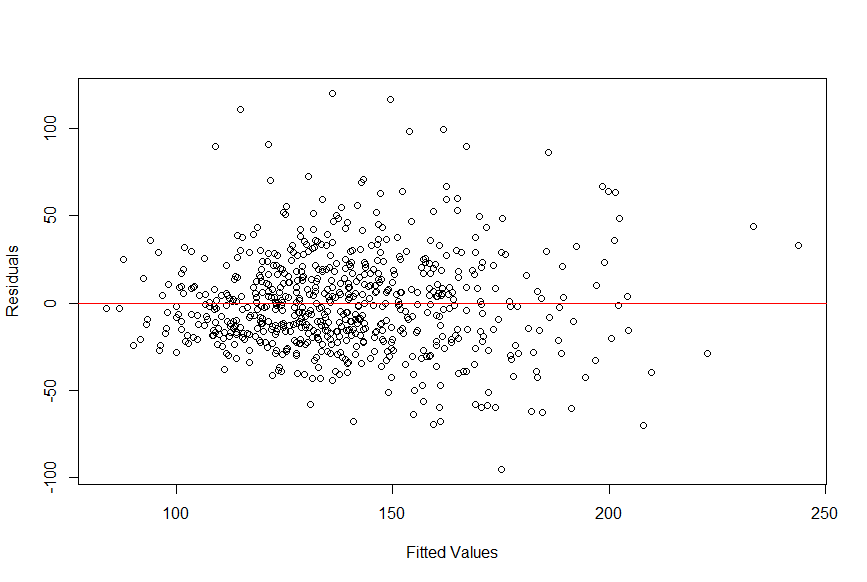


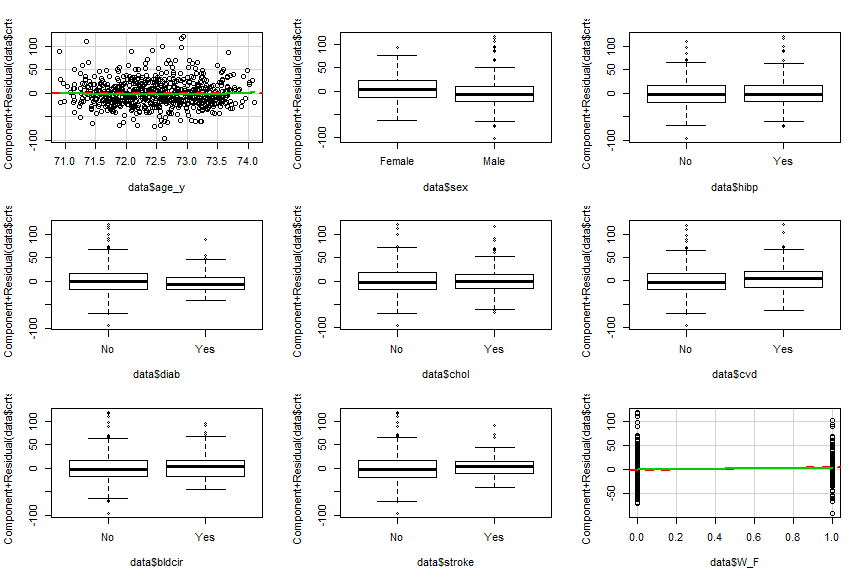


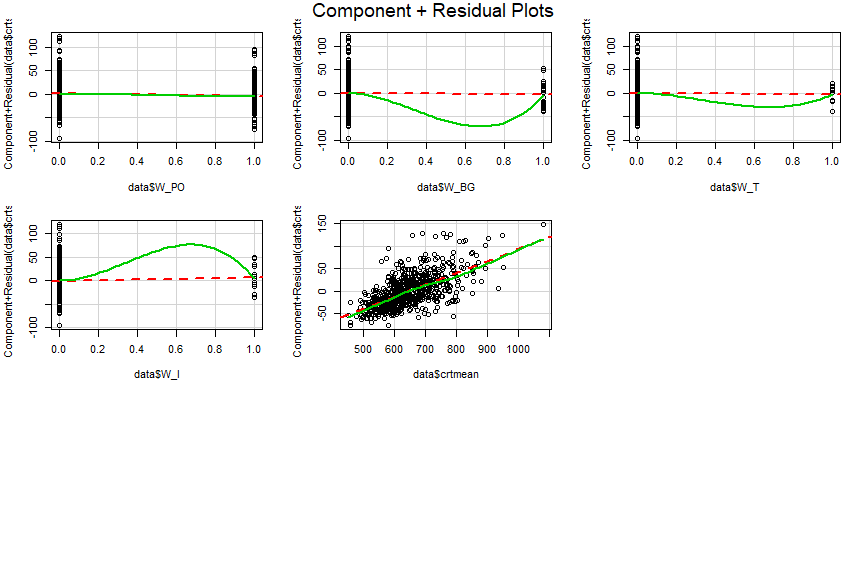


Table S4: Regression Model Results for Four-Choice Reaction Time SD Average White Matter Tract Fractional Anisotropy (n=647)

|  |  | **Model 1** |  |  | **Model 2** |  |
| --- | --- | --- | --- | --- | --- | --- |
|  | **b** | ***se*** | ***p*-value** | **b** | ***se*** | ***p*-value** |
| Age | 4.602 | 2.065 | 0.026 | 0.202 | 1.632 | 0.902 |
| Sex | -8.491 | 2.955 | 0.004 | -10.546 | 2.317 | <.001 |
|  |  |  |  |  |  |  |
| Hypertension | -0.872 | 3.054 | 0.775 | 0.196 | 2.392 | 0.935 |
| Diabetes | 4.010 | 4.920 | 0.415 | -1.932 | 3.865 | 0.617 |
| Cholesterol | 3.202 | 3.169 | 0.313 | -0.163 | 2.488 | 0.948 |
| CVD | 5.916 | 3.368 | 0.079 | 5.607 | 2.638 | 0.034 |
| Blood Circulation | 7.516 | 3.846 | 0.051 | 5.670 | 3.013 | 0.060 |
| Stroke | 8.742 | 5.723 | 0.127 | 7.359 | 4.483 | 0.101 |
|  |  |  |  |  |  |  |
| gFA | 2.294 | 1.592 | 0.150 | -1.576 | 1.261 | 0.212 |
|  |  |  |  |  |  |  |
| CRT Mean | - | - | - | 0.268 | 0.013 | <.001 |
| F | 3.678 | (9, 638) | <.001 | 45.700 | (10, 637) | <.001 |
| R-square | 0.049 |  |  | 0.418 |  |  |
| Adjusted R-square | 0.036 |  |  | 0.409 |  |  |

*Notes:* WMT gFA = white matter tract general fractional anisotropy factor; CVD = cardiovascular disease; CRT = choice reaction time.

***Assumptions***

Max VIF = 1.20


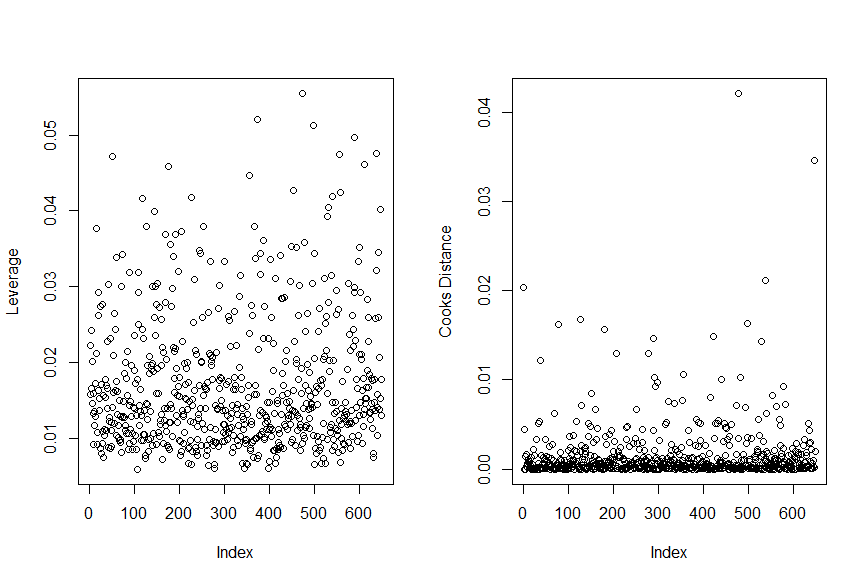


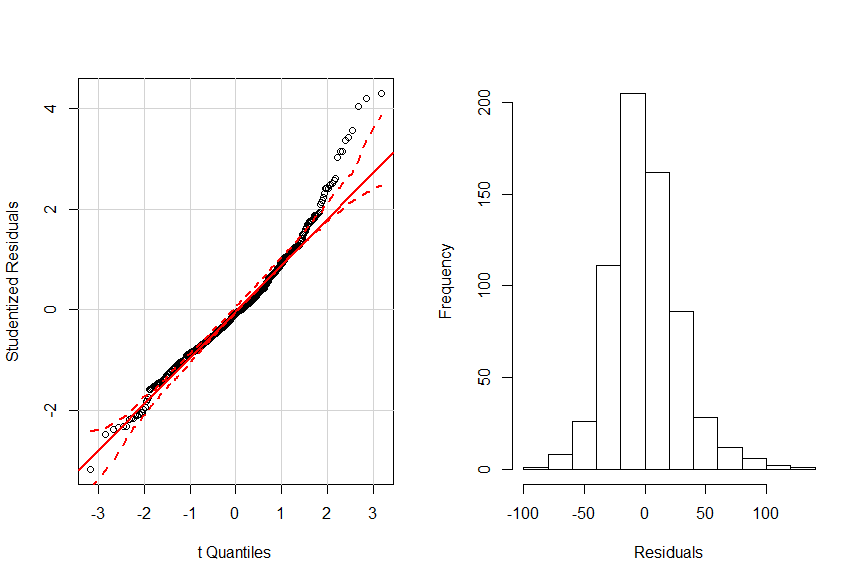


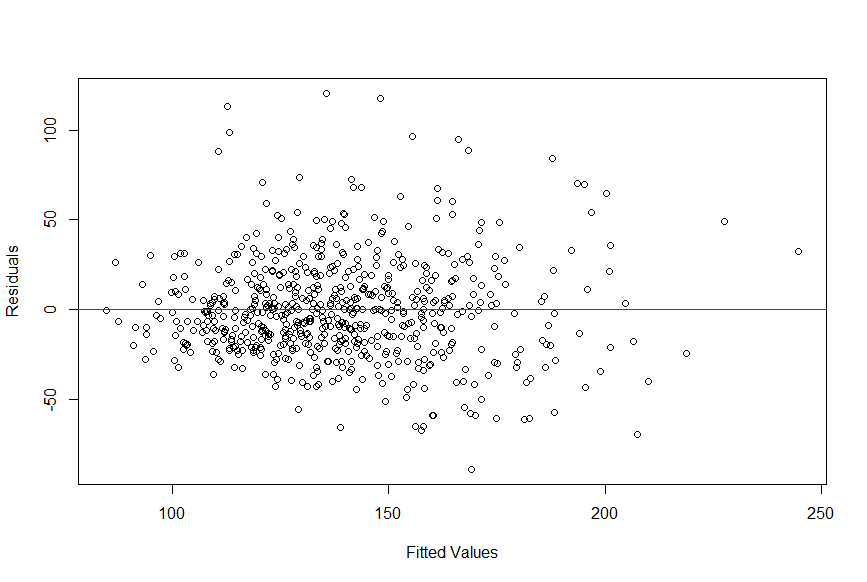


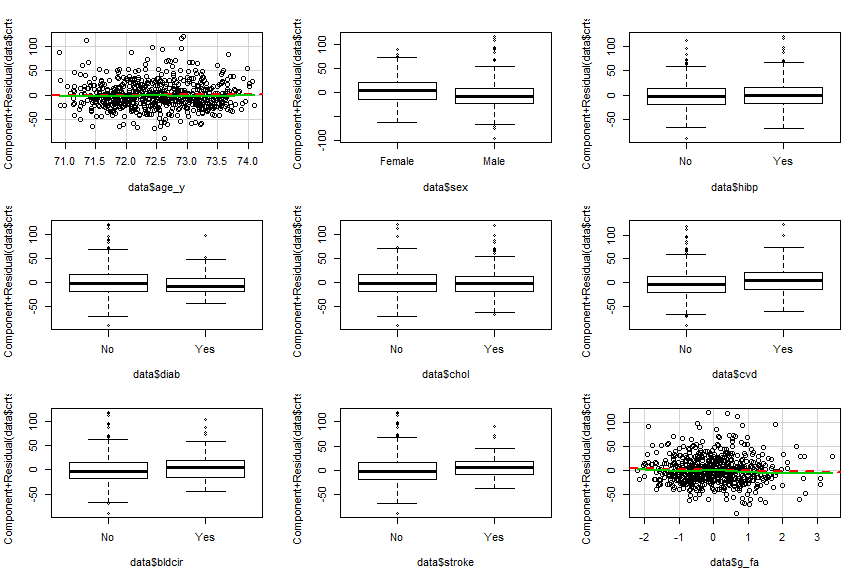


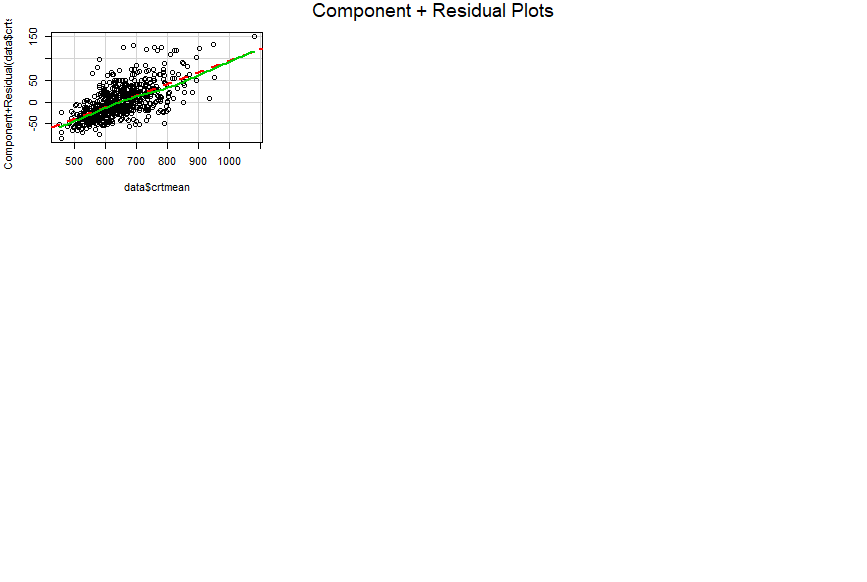


Table S5: Regression Model Results for Four-Choice Reaction Time SD on Average White Matter Tract Mean Diffusivity (n=647)

|  |  | **Model 1** |  |  | **Model 2** |  |
| --- | --- | --- | --- | --- | --- | --- |
|  | **b** | ***se*** | ***p*-value** | **b** | ***se*** | ***p*-value** |
| Age | 4.704 | 2.076 | 0.024 | 0.259 | 1.639 | 0.874 |
| Sex | -8.431 | 2.961 | 0.005 | -10.613 | 2.320 | <.001 |
|  |  |  |  |  |  |  |
| Hypertension | -0.421 | 3.040 | 0.890 | -0.087 | 2.379 | 0.971 |
| Diabetes | 3.863 | 4.930 | 0.434 | -1.724 | 3.868 | 0.656 |
| Cholesterol | 3.004 | 3.170 | 0.344 | -0.031 | 2.485 | 0.990 |
| CVD | 6.092 | 3.371 | 0.071 | 5.491 | 2.638 | 0.038 |
| Blood Circulation | 7.521 | 3.851 | 0.051 | 5.691 | 3.015 | 0.060 |
| Stroke | 9.106 | 5.724 | 0.112 | 7.165 | 4.480 | 0.110 |
|  |  |  |  |  |  |  |
| gMD | 0.880 | 1.576 | 0.577 | -1.239 | 1.238 | 0.317 |
|  |  |  |  |  |  |  |
| CRT Mean | - | - | - | 0.267 | 0.013 | <.001 |
| F | 3.472 | (9, 638) | <.001 | 45.600 | (10, 637) | <.001 |
| R-square | 0.047 |  |  | 0.417 |  |  |
| Adjusted R-square | 0.033 |  |  | 0.408 |  |  |

*Notes:* WMT gMD = white matter tract general mean diffusivity factor; CVD = cardiovascular disease; CRT = choice reaction time.

***Assumptions***

Max VIF = 1.20


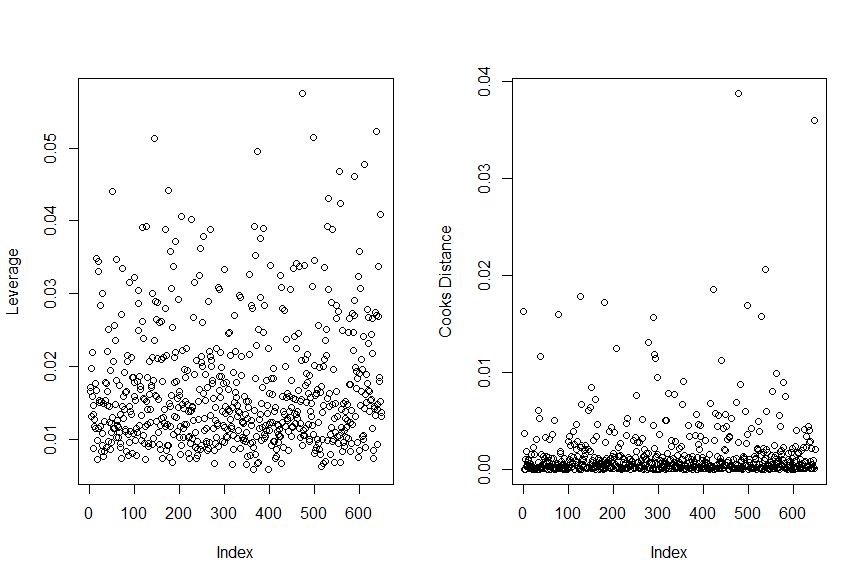


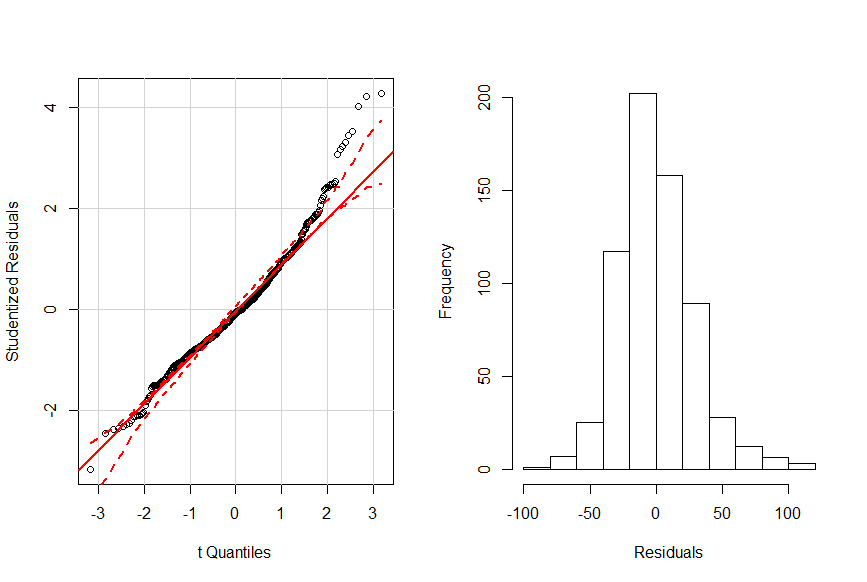


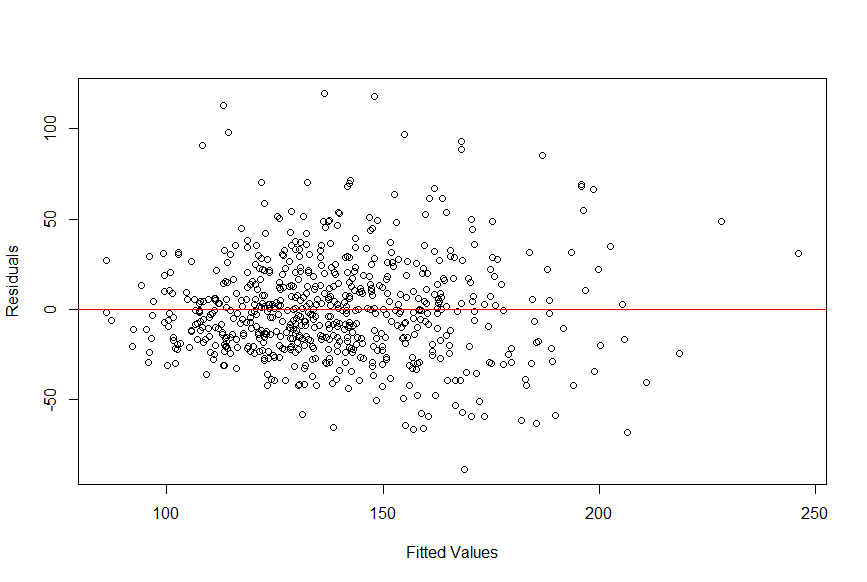


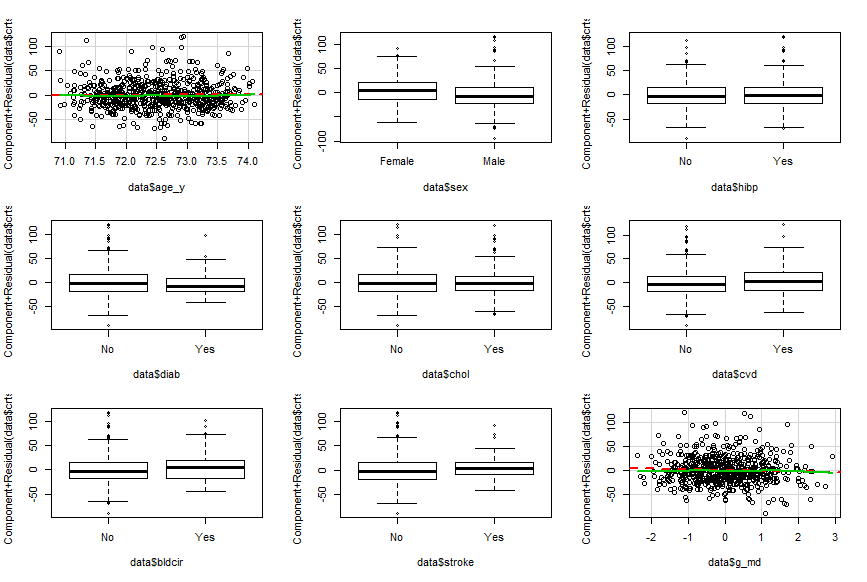


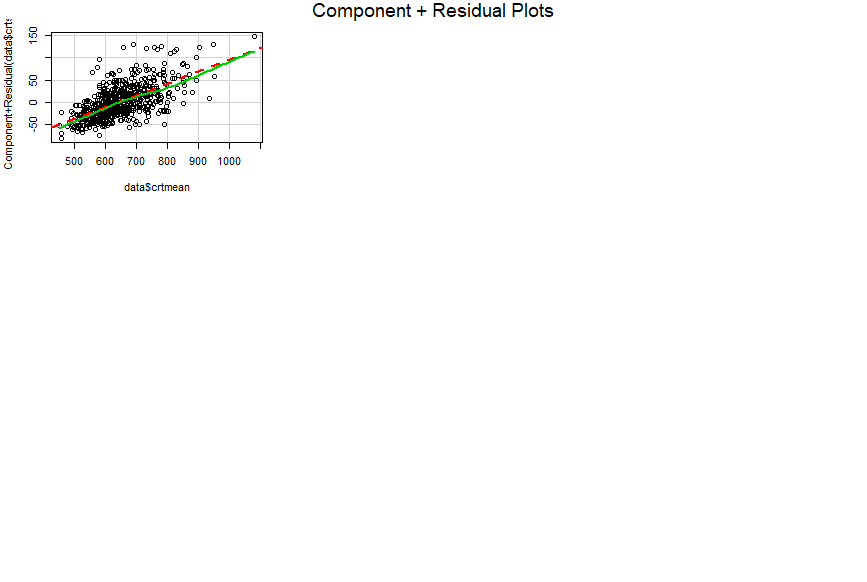


**CRT Mean**

Table S6: Regression Model Results for Four-Choice Reaction Time Mean and WMH Volume (n=670)

|  |  | **Model 1** |  |  | **Model 2** |  |
| --- | --- | --- | --- | --- | --- | --- |
|  | **b** | ***se*** | ***p*-value** | **b** | ***se*** | ***p*-value** |
| Age | 15.152 | 4.674 | 0.001 | 9.426 | 3.683 | 0.011 |
| Sex | 7.450 | 6.693 | 0.266 | 19.92 | 5.294 | <.001 |
|  |  |  |  |  |  |  |
| Hypertension | -3.061 | 6.959 | 0.660 | -1.914 | 5.468 | 0.726 |
| Diabetes | 21.257 | 11.092 | 0.056 | 15.733 | 8.718 | 0.072 |
| Cholesterol | 10.179 | 7.198 | 0.158 | 6.364 | 5.658 | 0.261 |
| CVD | 5.418 | 7.668 | 0.480 | -4.055 | 6.042 | 0.502 |
| Blood Circulation | 9.253 | 8.744 | 0.290 | -1.662 | 6.891 | 0.809 |
| Stroke | 4.681 | 13.216 | 0.723 | -6.829 | 10.398 | 0.512 |
|  |  |  |  |  |  |  |
| WMH Volume | 12.062 | 3.334 | <.001 | 6.419 | 2.634 | 0.015 |
|  |  |  |  |  |  |  |
| CRT SD | - | - | - | 1.449 | 0.072 | <.001 |
| F | 4.551 | (9, 660) | <.001 | 47.670 | (10, 659) | <.001 |
| R-square | 0.058 |  |  | 0.420 |  |  |
| Adjusted R-square | 0.046 |  |  | 0.411 |  |  |

*Notes:* WMH = white matter hyperintensity; CVD = cardiovascular disease; CRT = choice reaction time.

***Assumptions***

Max VIF = 1.19


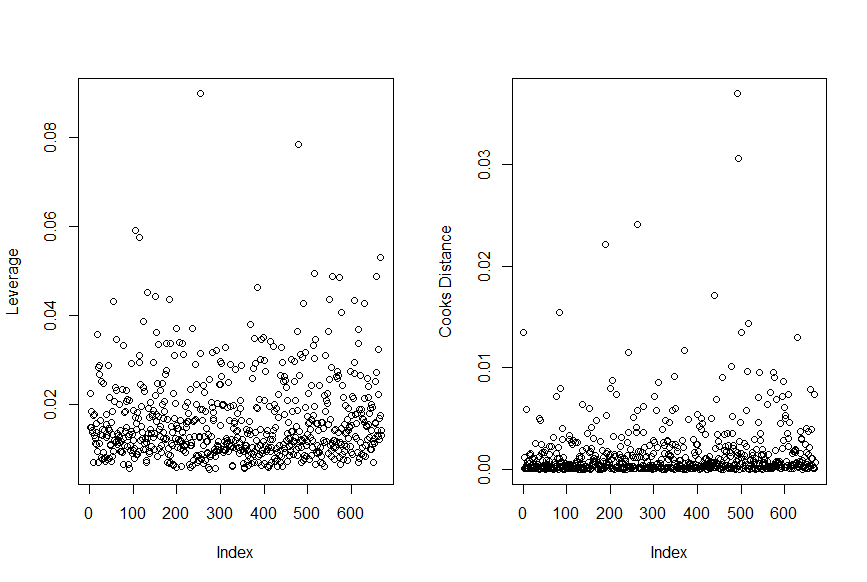


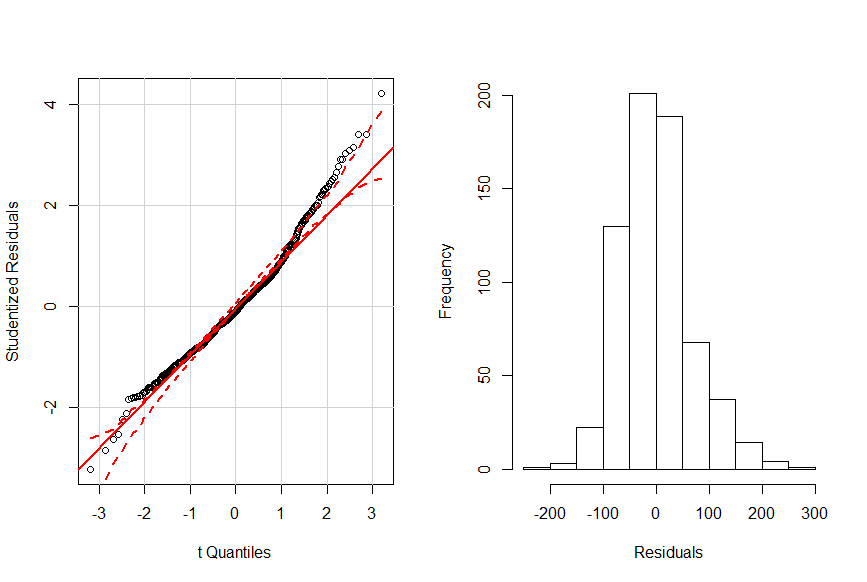


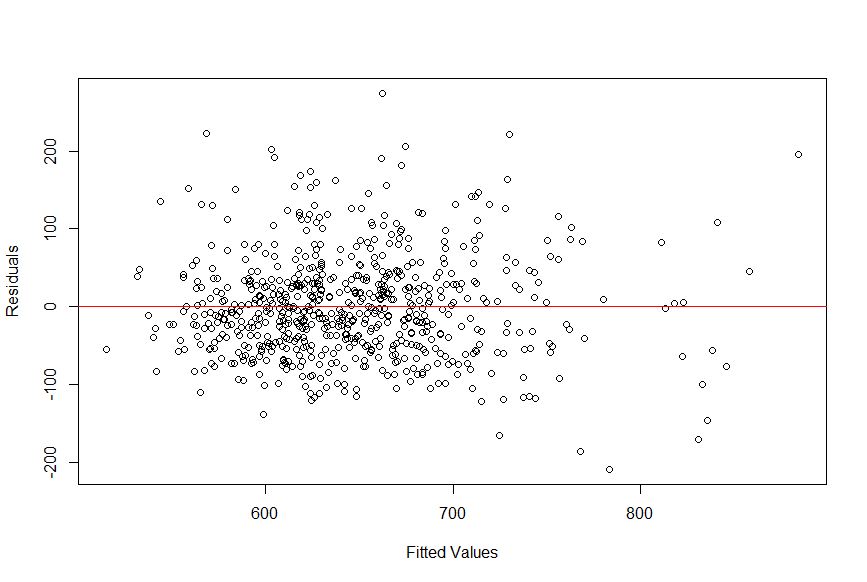


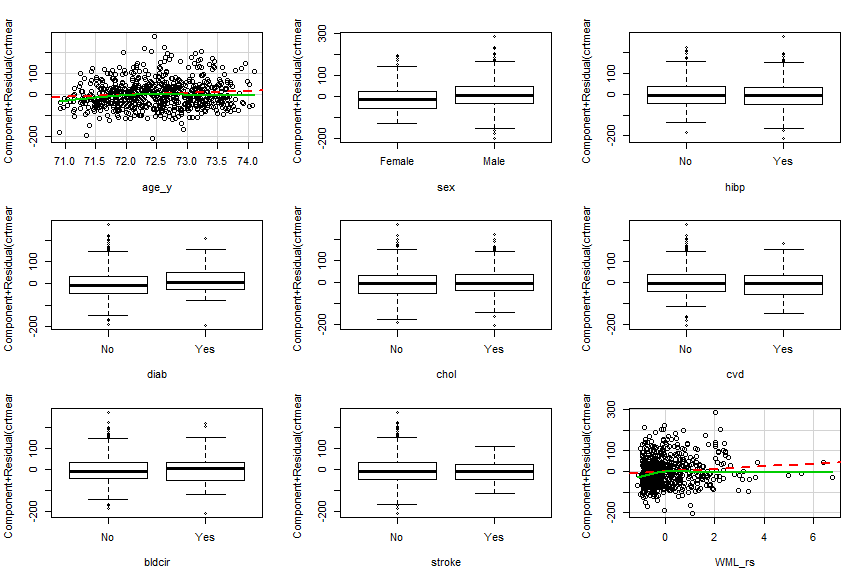


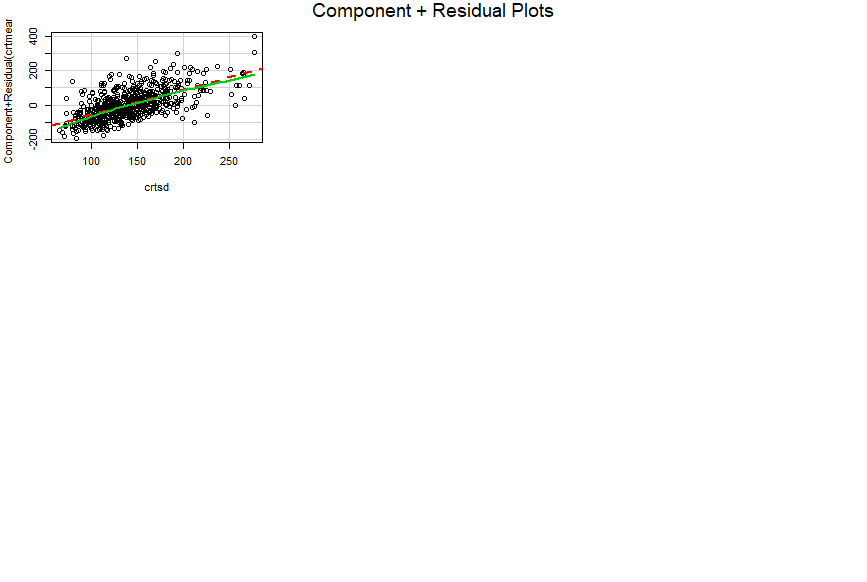


Table S7: Regression Model Results for Four-Choice Reaction Time Mean and WMH Severity in different brain regions (n=670)

|  |  | **Model 1** |  |  | **Model 2** |  |
| --- | --- | --- | --- | --- | --- | --- |
|  | **b** | ***se*** | ***p*-value** | **b** | ***se*** | ***p*-value** |
| Age | 16.407 | 4.625 | <.001 | 10.027 | 3.653 | 0.006 |
| Sex | 7.315 | 6.678 | 0.274 | 19.804 | 5.291 | <.001 |
|  |  |  |  |  |  |  |
| Hypertension | -2.570 | 6.953 | 0.712 | -2.244 | 5.47 | 0.682 |
| Diabetes | 20.276 | 11.100 | 0.068 | 15.911 | 8.736 | 0.069 |
| Cholesterol | 8.925 | 7.196 | 0.215 | 5.551 | 5.664 | 0.327 |
| CVD | 6.417 | 7.663 | 0.403 | -3.926 | 6.052 | 0.517 |
| Blood Circulation | 9.419 | 8.760 | 0.283 | -1.814 | 6.915 | 0.793 |
| Stroke | 6.988 | 13.342 | 0.601 | -5.346 | 10.516 | 0.611 |
|  |  |  |  |  |  |  |
| Wahlund: Frontal | 28.658 | 9.288 | 0.002 | 10.747 | 7.362 | 0.145 |
| Wahlund: Parieto-Occipital | 3.119 | 8.968 | 0.728 | 10.511 | 7.066 | 0.137 |
| Wahlund: Basal Ganglia | -33.818 | 19.350 | 0.081 | -16.668 | 15.248 | 0.275 |
| Wahlund: Temporal | 41.913 | 31.778 | 0.188 | 29.955 | 25.01 | 0.231 |
| Wahlund: Infratentorial | 36.862 | 23.858 | 0.123 | 13.703 | 18.807 | 0.467 |
|  |  |  |  |  |  |  |
| CRT SD | - | - | - | 1.442 | 0.072 | <.001 |
| F | 3.915 | (13, 656) | <.001 | 34.780 | (14, 654) | <.001 |
| R-square | 0.072 |  |  | 0.426 |  |  |
| Adjusted R-square | 0.054 |  |  | 0.414 |  |  |

*Notes:* CVD = cardiovascular disease; CRT = choice reaction time.

***Assumptions***

Max VIF = 1.63


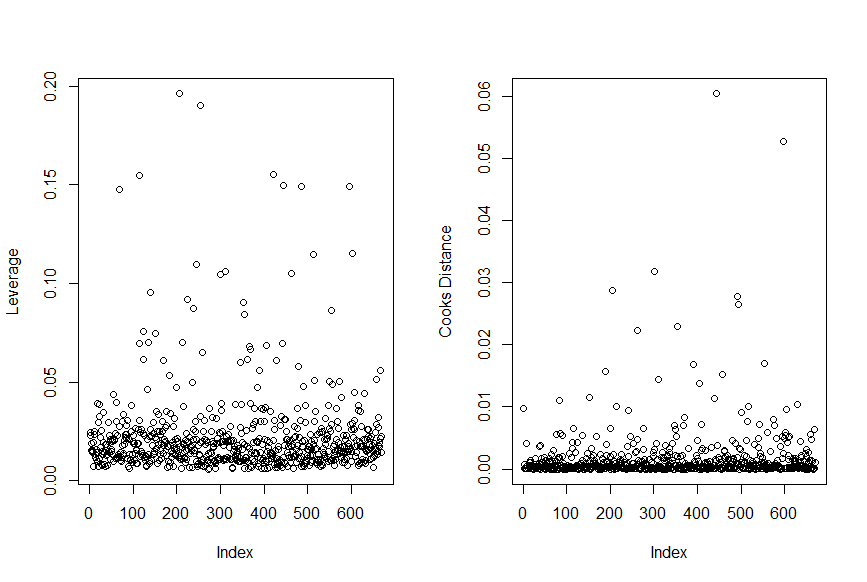


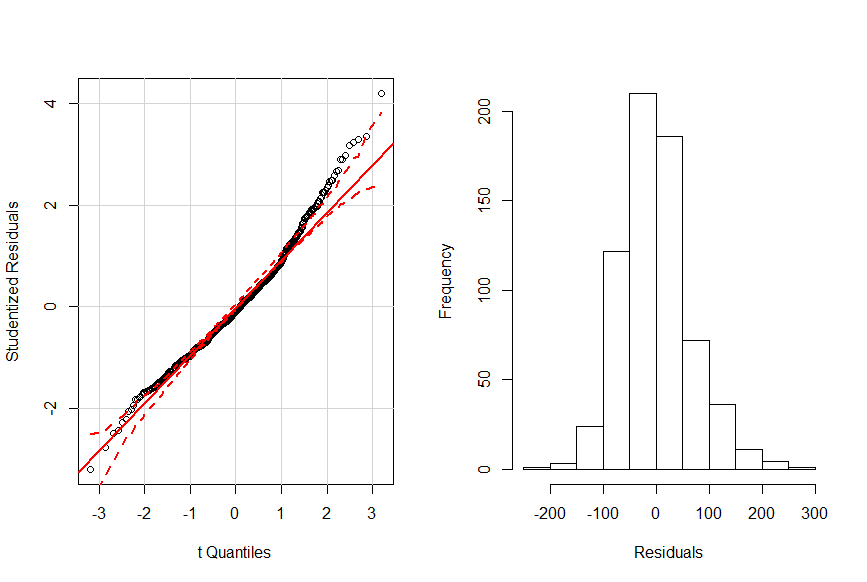


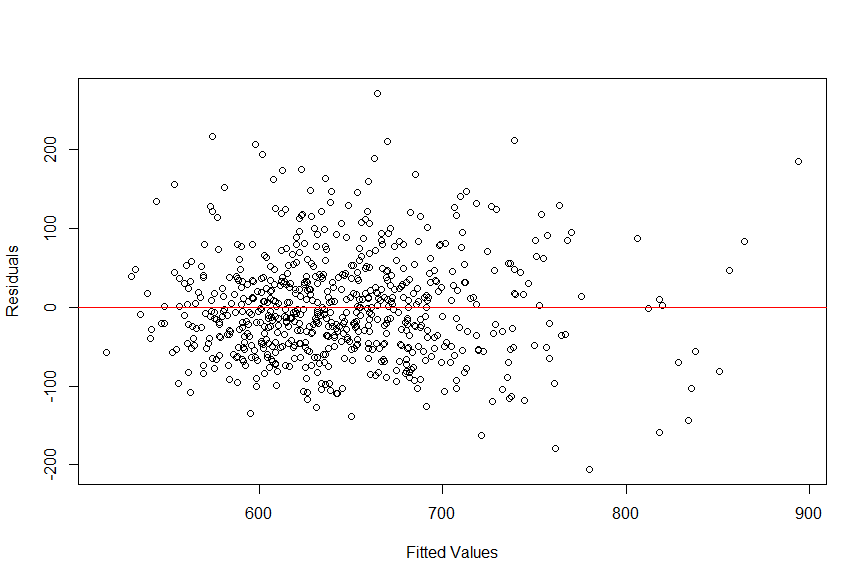


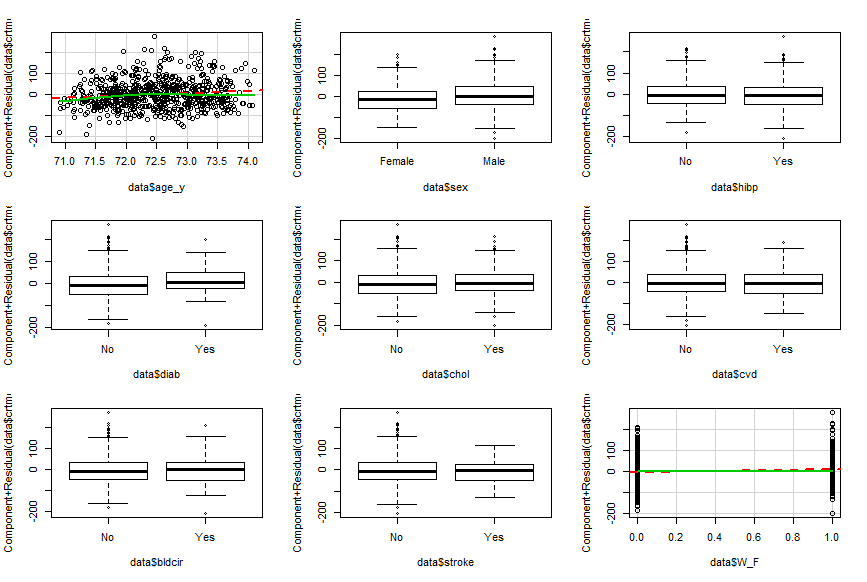


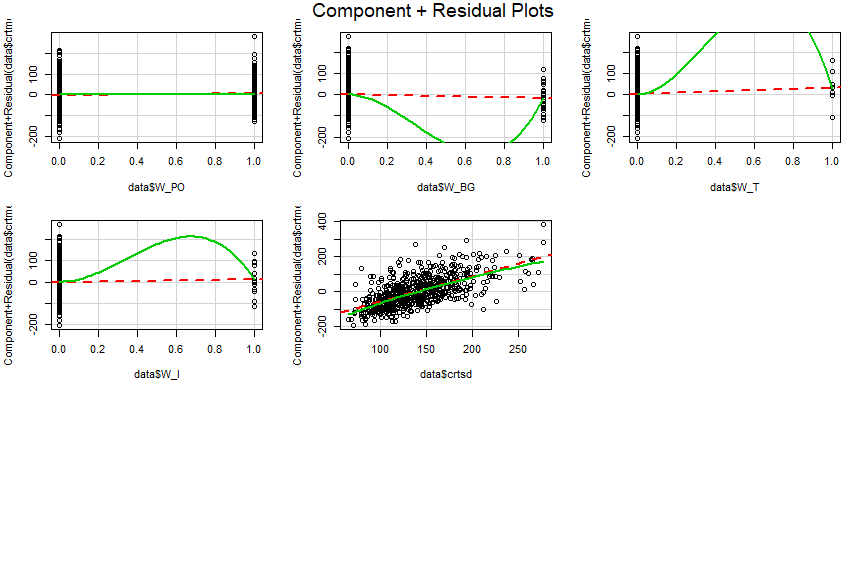


Table S8: Regression Model Results for Four-Choice Reaction Time Mean Average White Matter Tract Fractional Anisotropy (n=647)

|  |  | **Model 1** |  |  | **Model 2** |  |
| --- | --- | --- | --- | --- | --- | --- |
|  | **b** | ***se*** | ***p*-value** | **b** | ***se*** | ***p*-value** |
| Age | 16.399 | 4.790 | 0.001 | 9.753 | 3.766 | 0.010 |
| Sex | 7.658 | 6.857 | 0.264 | 19.921 | 5.405 | <.001 |
|  |  |  |  |  |  |  |
| Hypertension | -3.981 | 7.085 | 0.574 | -2.722 | 5.550 | 0.624 |
| Diabetes | 22.147 | 11.415 | 0.053 | 16.354 | 8.945 | 0.068 |
| Cholesterol | 12.543 | 7.353 | 0.089 | 7.918 | 5.764 | 0.170 |
| CVD | 1.153 | 7.815 | 0.883 | -7.392 | 6.135 | 0.229 |
| Blood Circulation | 6.882 | 8.922 | 0.441 | -3.973 | 7.009 | 0.571 |
| Stroke | 5.154 | 13.278 | 0.698 | -7.471 | 10.419 | 0.474 |
|  |  |  |  |  |  |  |
| gFA | 14.421 | 3.693 | <.001 | 11.109 | 2.897 | <.001 |
|  |  |  |  |  |  |  |
| CRT SD | - | - | - | 1.444 | 0.072 | <.001 |
| F | 4.684 | (9, 638) | <.001 | 47.170 | (10, 637) | <.001 |
| R-square | 0.062 |  |  | 0.426 |  |  |
| Adjusted R-square | 0.049 |  |  | 0.417 |  |  |

*Notes:* WMT gFA = white matter tract general fractional anisotropy factor; CVD = cardiovascular disease; CRT = choice reaction time.

***Assumptions***

Max VIF = 1.4


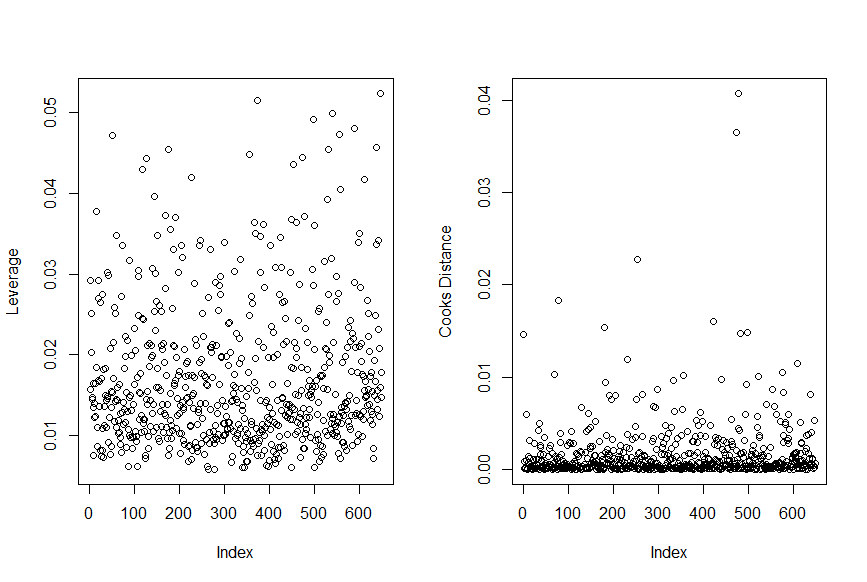


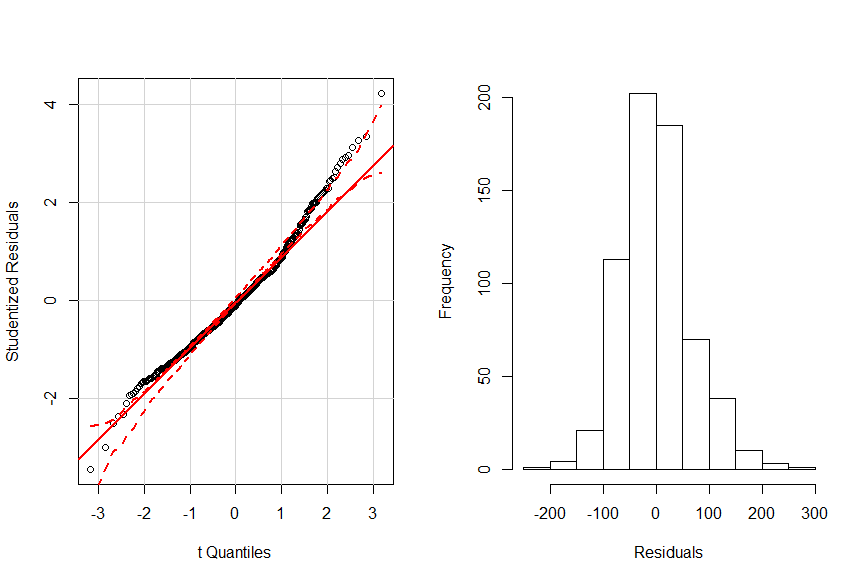


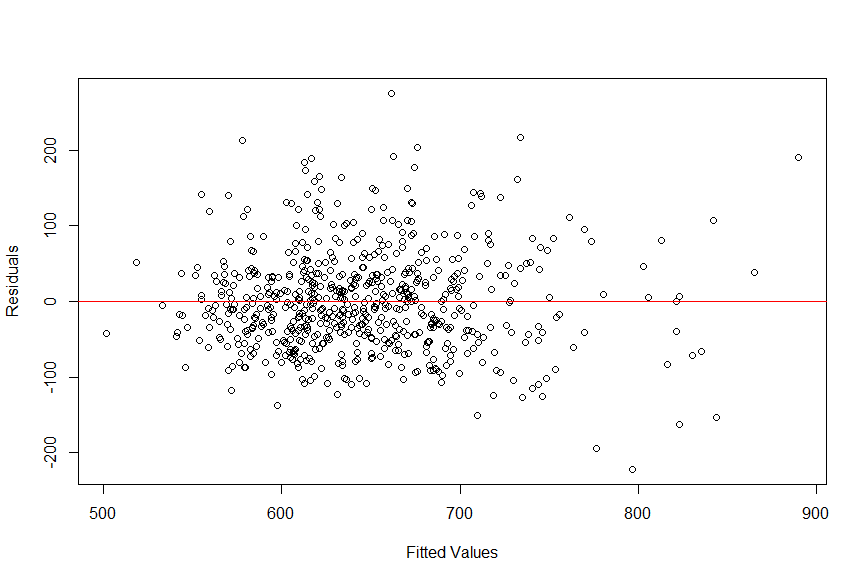


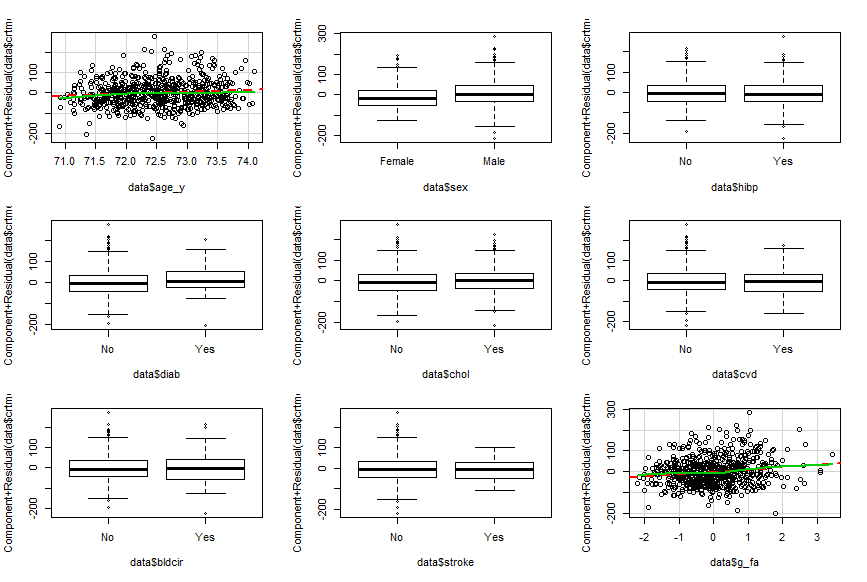


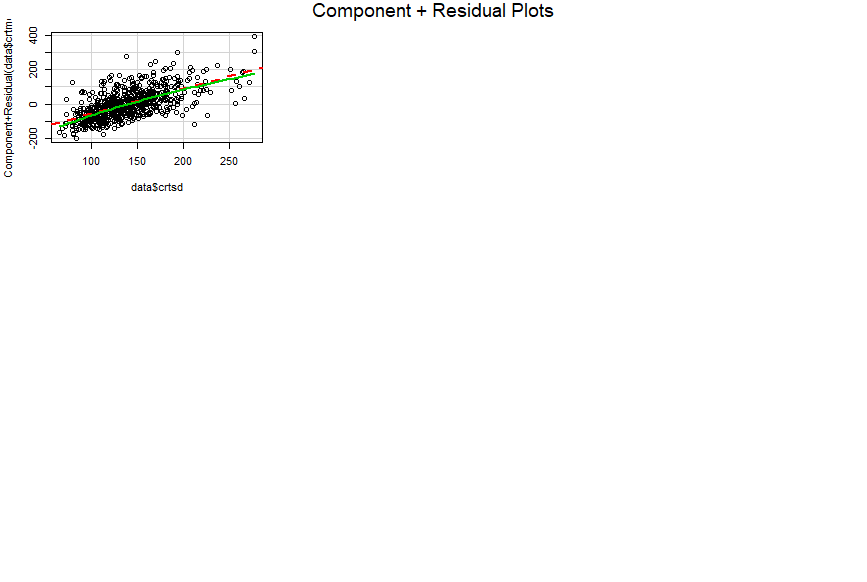


Table S9: Regression Model Results for Four-Choice Reaction Time Mean on Average White Matter Tract Mean Diffusivity (n=647)

|  |  | **Model 1** |  |  | **Model 2** |  |
| --- | --- | --- | --- | --- | --- | --- |
|  | **b** | ***se*** | ***p*-value** | **b** | ***se*** | ***p*-value** |
| Age | 16.653 | 4.850 | 0.001 | 9.803 | 3.810 | 0.010 |
| Sex | 8.175 | 6.917 | 0.238 | 20.453 | 5.447 | <.001 |
|  |  |  |  |  |  |  |
| Hypertension | -1.253 | 7.101 | 0.860 | -0.640 | 5.557 | 0.908 |
| Diabetes | 20.932 | 11.517 | 0.070 | 15.307 | 9.016 | 0.090 |
| Cholesterol | 11.372 | 7.405 | 0.125 | 6.998 | 5.799 | 0.228 |
| CVD | 2.254 | 7.873 | 0.775 | -6.619 | 6.177 | 0.284 |
| Blood Circulation | 6.859 | 8.996 | 0.446 | -4.095 | 7.06 | 0.562 |
| Stroke | 7.272 | 13.371 | 0.587 | -5.989 | 10.484 | 0.568 |
|  |  |  |  |  |  |  |
| gMD | 7.942 | 3.681 | 0.031 | 6.660 | 2.881 | 0.021 |
|  |  |  |  |  |  |  |
| CRT SD | - | - | - | 1.456 | 0.072 | <.001 |
| F | 3.459 | (9, 638) | <.001 | 45.580 | (10, 637) | <.001 |
| R-square | 0.047 |  |  | 0.417 |  |  |
| Adjusted R-square | 0.033 |  |  | 0.408 |  |  |

*Notes:* WMT gMD = white matter tract general mean diffusivity factor; CVD = cardiovascular disease; CRT = choice reaction time.

***Assumptions***

Max VIF = 1.19


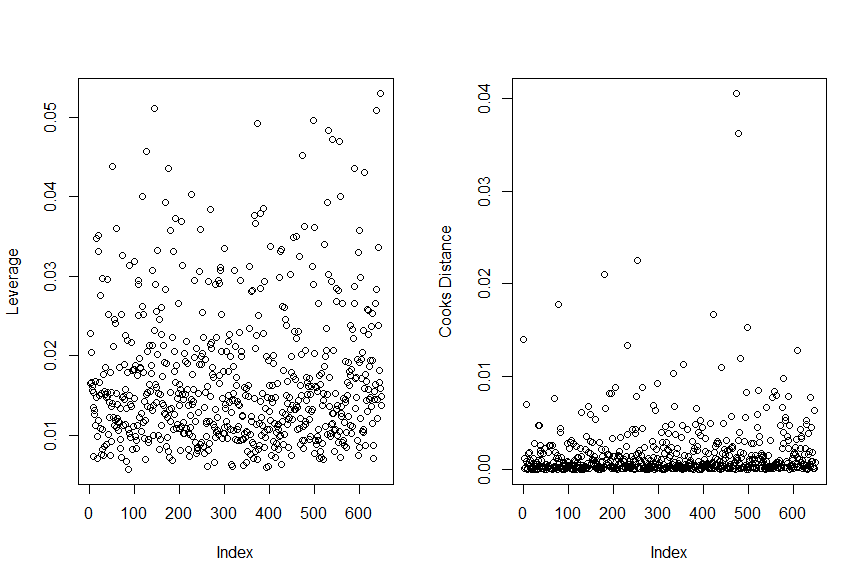


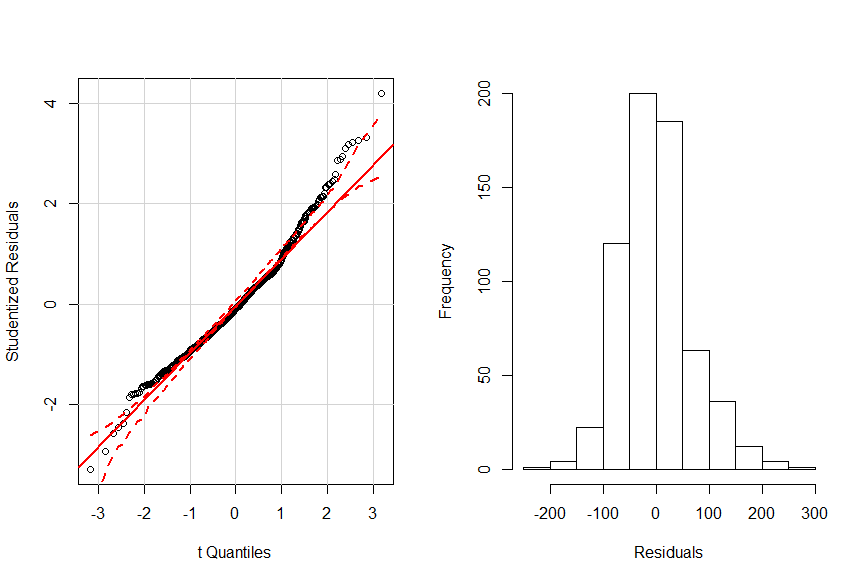


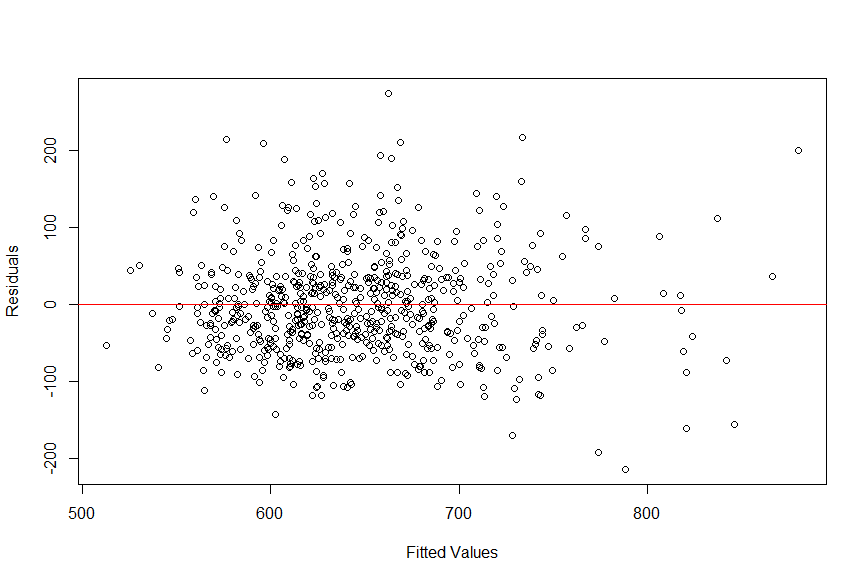


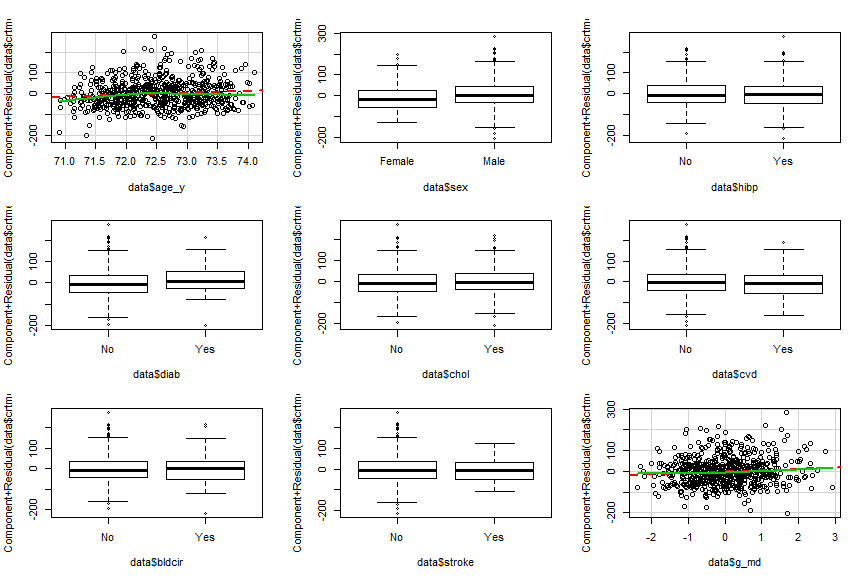


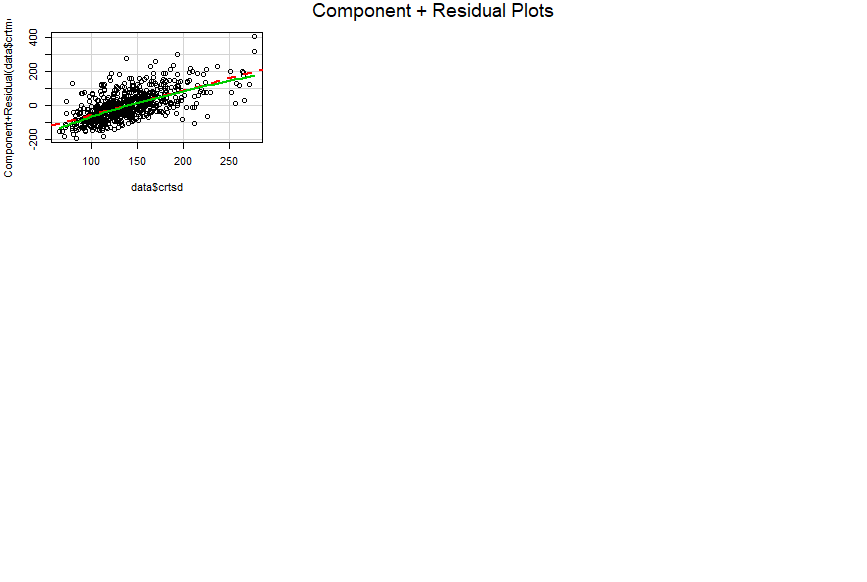


**CRT CV**

Table S10: Regression Model Results for Four-Choice Reaction Time CV and WMH Volume (n=670)

|  |  | **Model 1** |  |
| --- | --- | --- | --- |
|  | **b** | ***se*** | ***p*-value** |
| Age | 0.001 | 0.002 | 0.798 |
| Sex | -0.016 | 0.003 | <.001 |
|  |  |  |  |
| Hypertension | 0.000 | 0.004 | 0.901 |
| Diabetes | -0.001 | 0.006 | 0.797 |
| Cholesterol | 0.000 | 0.004 | 0.973 |
| CVD | 0.009 | 0.004 | 0.031 |
| Blood Circulation | 0.008 | 0.004 | 0.063 |
| Stroke | 0.010 | 0.007 | 0.134 |
|  |  |  |  |
| WMH Volume | 0.002 | 0.002 | 0.224 |
| F | 3.857 | (9, 660) | <.001 |
| R-square | 0.050 |  |  |
| Adjusted R-square | 0.037 |  |  |

*Notes:* WMH = white matter hyperintensity; CVD = cardiovascular disease.

***Assumptions***

Max VIF = 1.19


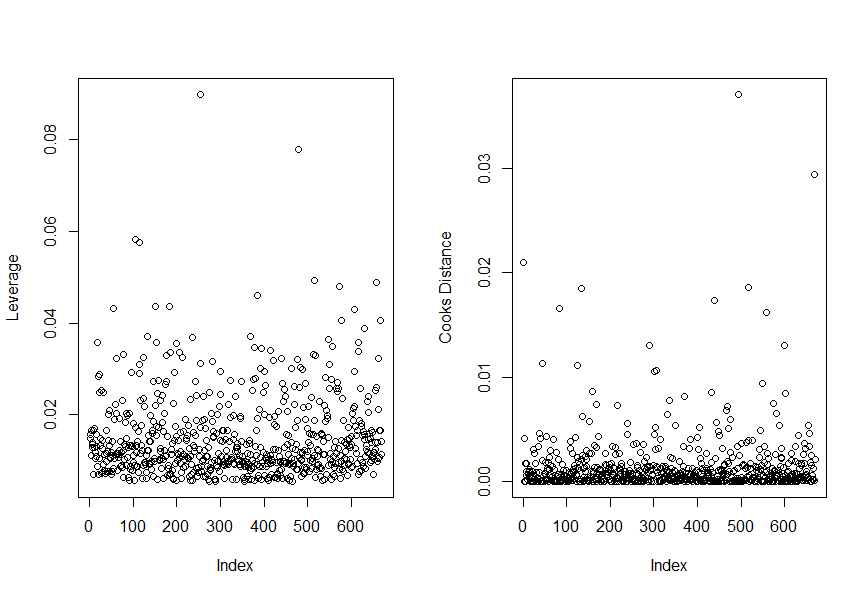


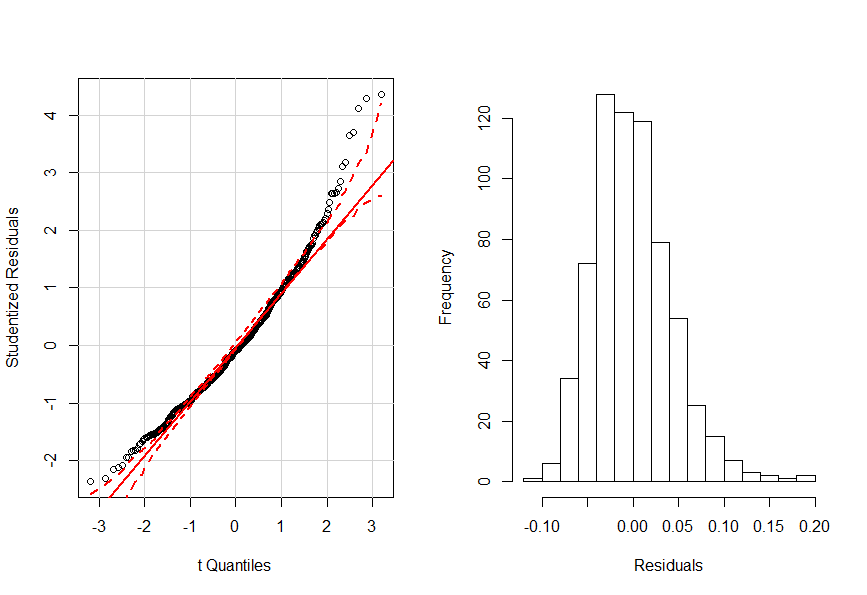


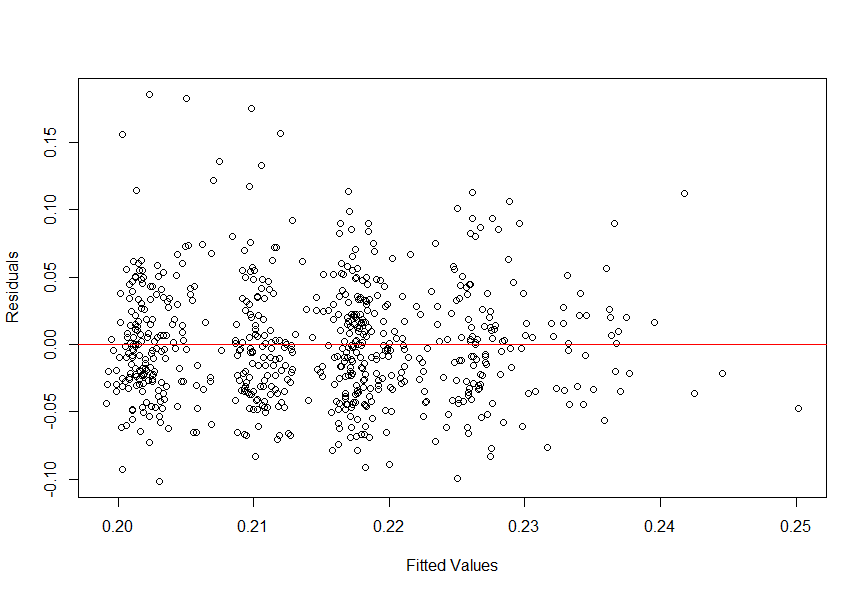


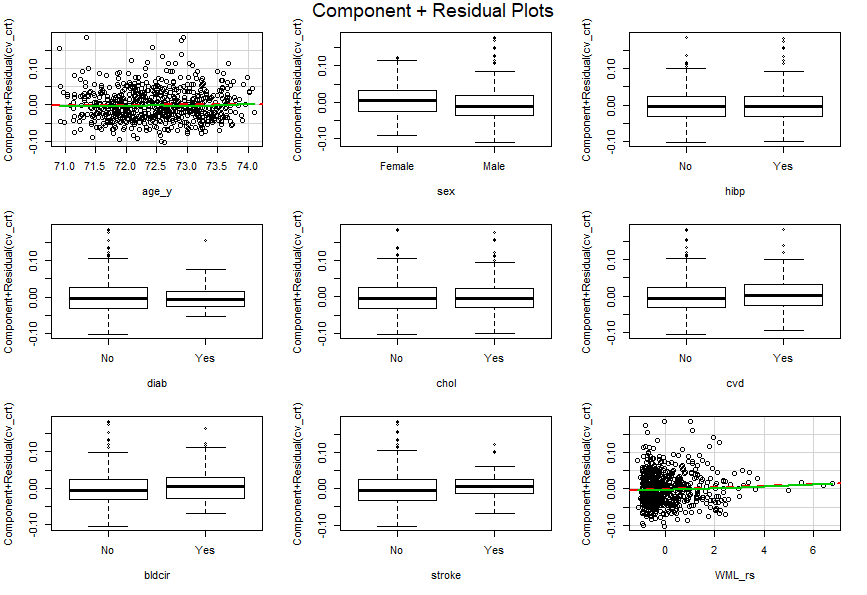


Table S11: Regression Model Results for Four-Choice Reaction Time CV and WMH Severity in different brain regions (n=670)

|  |  | **Model 1** |  |
| --- | --- | --- | --- |
|  | **b** | ***se*** | ***p*-value** |
| Age | 0.001 | 0.002 | 0.689 |
| Sex | -0.016 | 0.003 | <.001 |
|  |  |  |  |
| Hypertension | 0.000 | 0.004 | 0.932 |
| Diabetes | -0.002 | 0.006 | 0.669 |
| Cholesterol | 0.000 | 0.004 | 0.977 |
| CVD | 0.009 | 0.004 | 0.021 |
| Blood Circulation | 0.009 | 0.005 | 0.053 |
| Stroke | 0.010 | 0.007 | 0.129 |
|  |  |  |  |
| Wahlund: Frontal | 0.010 | 0.005 | 0.040 |
| Wahlund: Parieto-Occipital | -0.010 | 0.005 | 0.039 |
| Wahlund: Basal Ganglia | -0.007 | 0.010 | 0.463 |
| Wahlund: Temporal | 0.001 | 0.016 | 0.964 |
| Wahlund: Infratentorial | 0.013 | 0.012 | 0.309 |
| F | 3.068 | (13, 656) | <.001 |
| R-square | 0.573 |  |  |
| Adjusted R-square | 0.039 |  |  |

*Notes:* CVD = cardiovascular disease.

***Assumptions***

Max VIF = 1.61


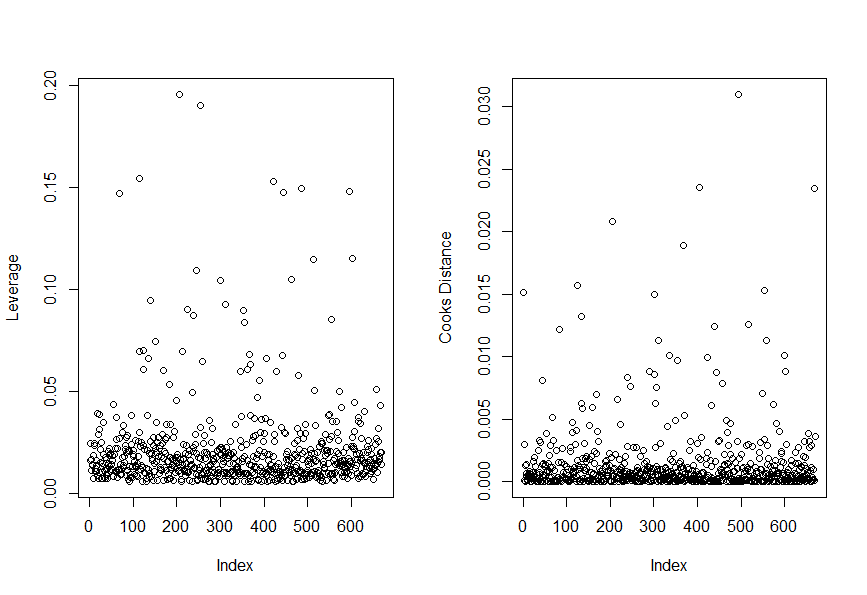


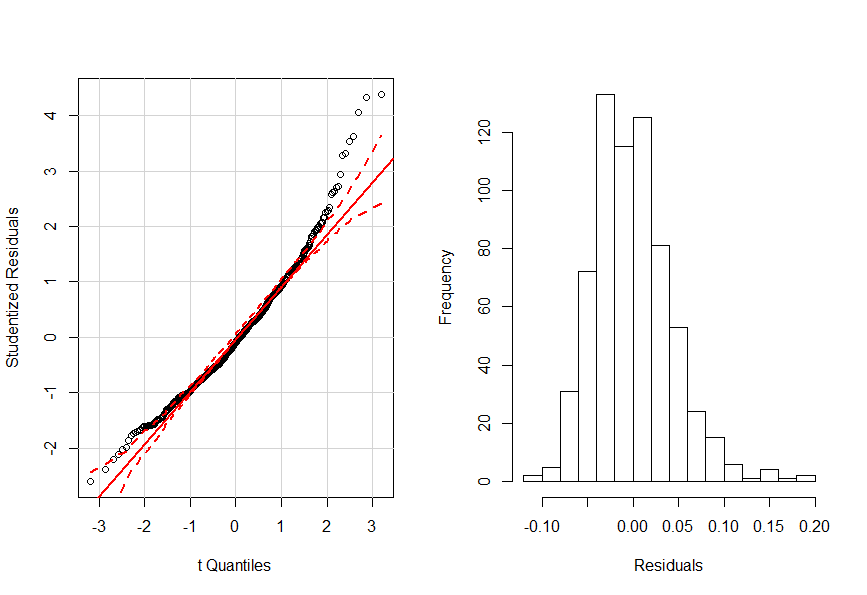


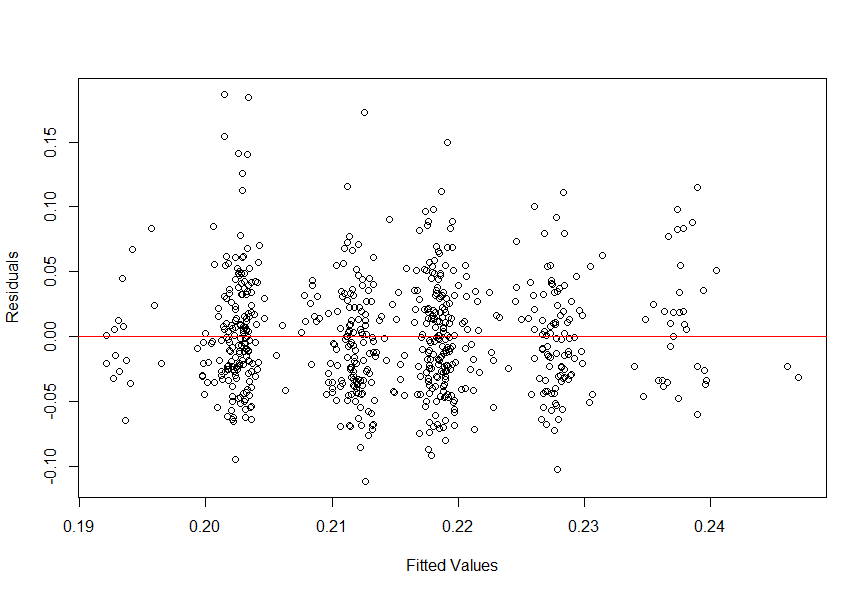


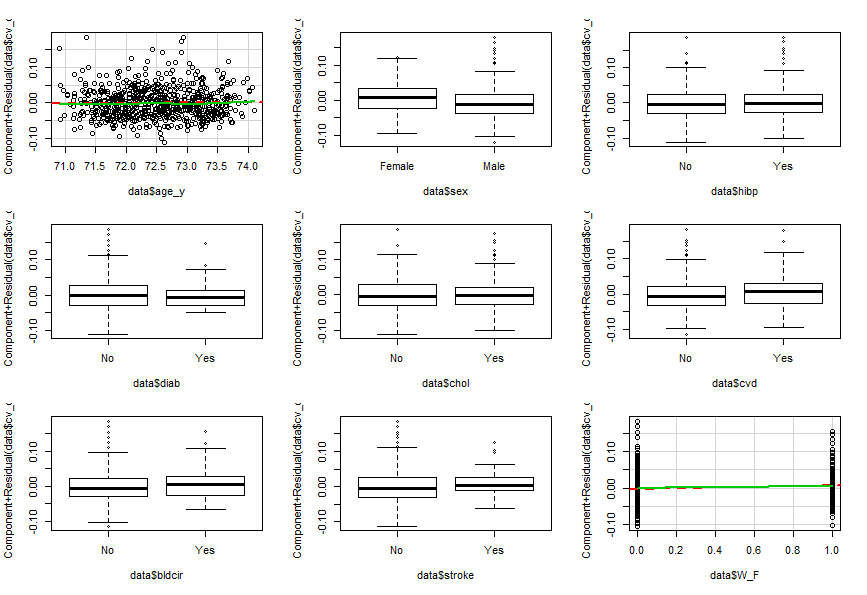


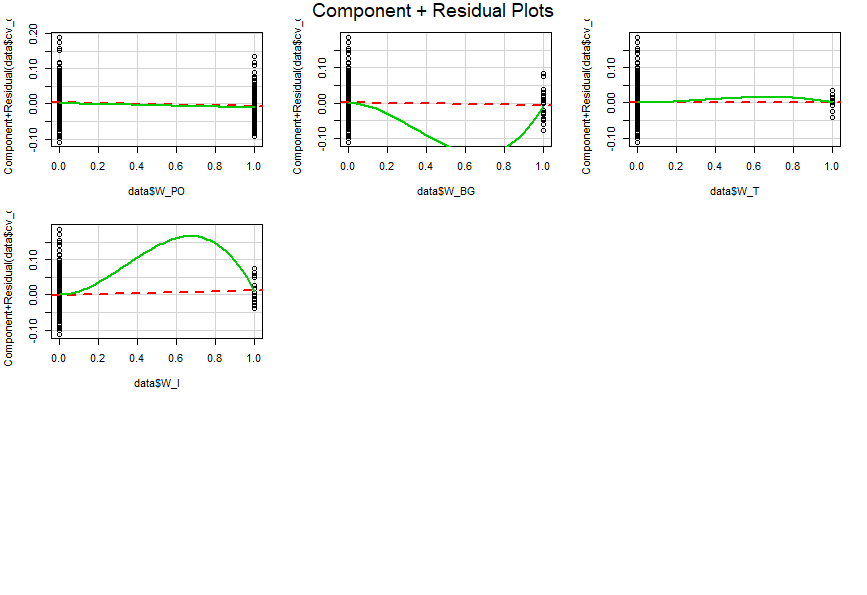


Table S12: Regression Model Results for Four-Choice Reaction Time CV Average White Matter Tract Fractional Anisotropy (n=647)

|  |  | **Model 1** |  |
| --- | --- | --- | --- |
|  | **b** | ***se*** | ***p*-value** |
| Age | 0.001 | 0.002 | 0.626 |
| Sex | -0.015 | 0.004 | <.001 |
|  |  |  |  |
| Hypertension | 0.000 | 0.004 | 0.930 |
| Diabetes | -0.002 | 0.006 | 0.793 |
| Cholesterol | 0.000 | 0.004 | 0.922 |
| CVD | 0.009 | 0.004 | 0.027 |
| Blood Circulation | 0.009 | 0.005 | 0.046 |
| Stroke | 0.011 | 0.007 | 0.104 |
|  |  |  |  |
| gFA | -0.001 | 0.002 | 0.658 |
| F | 3.611 | (9, 638) | <.001 |
| R-square | 0.049 |  |  |
| Adjusted R-square | 0.035 |  |  |

*Notes:* WMT gFA = white matter tract general fractional anisotropy factor; CVD = cardiovascular disease.

***Assumptions***

Max VIF = 1.20


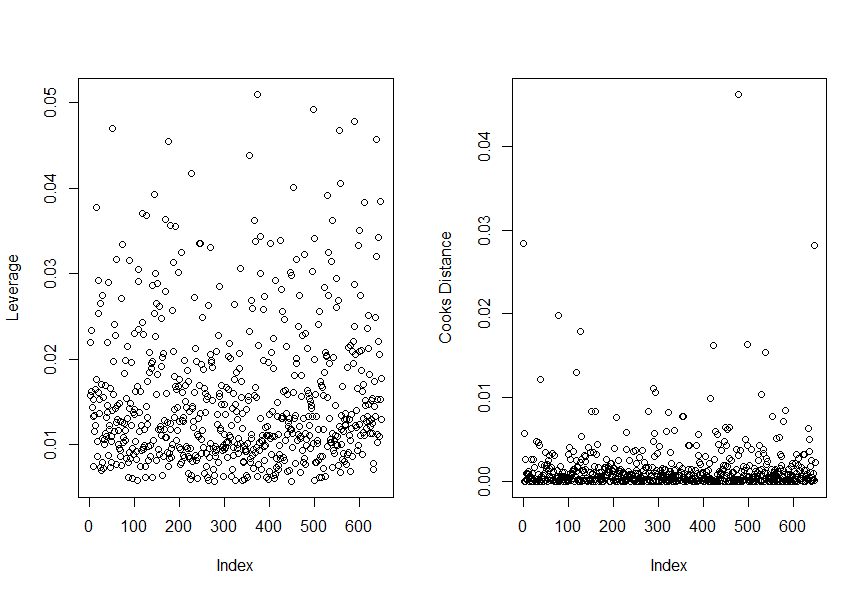


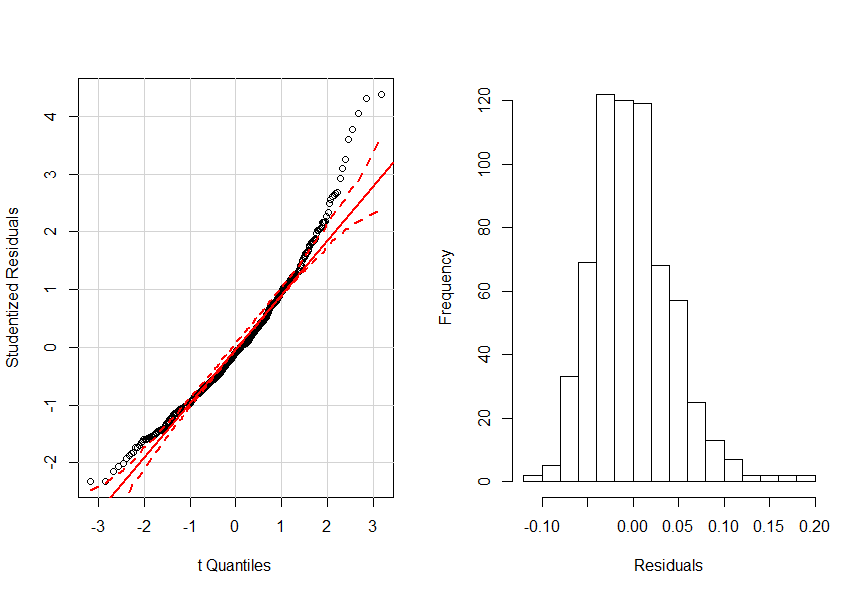


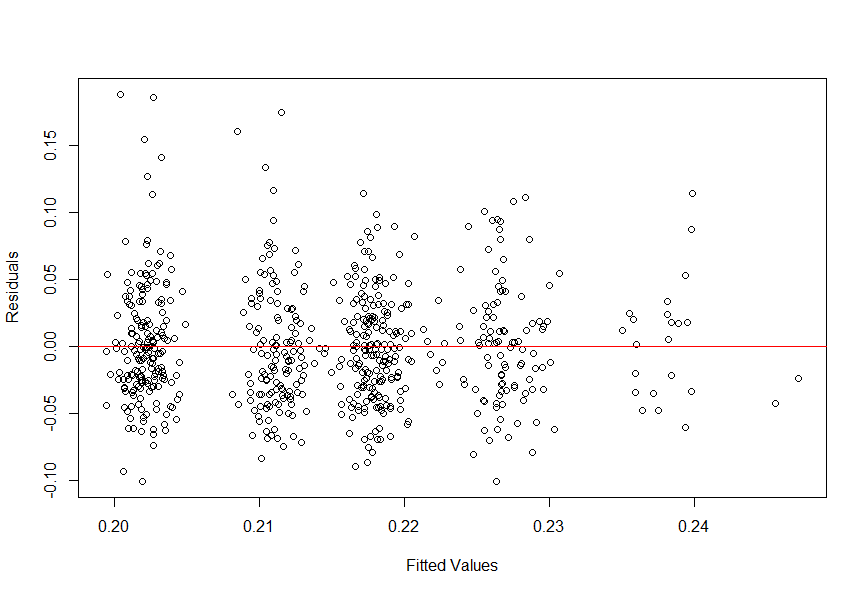


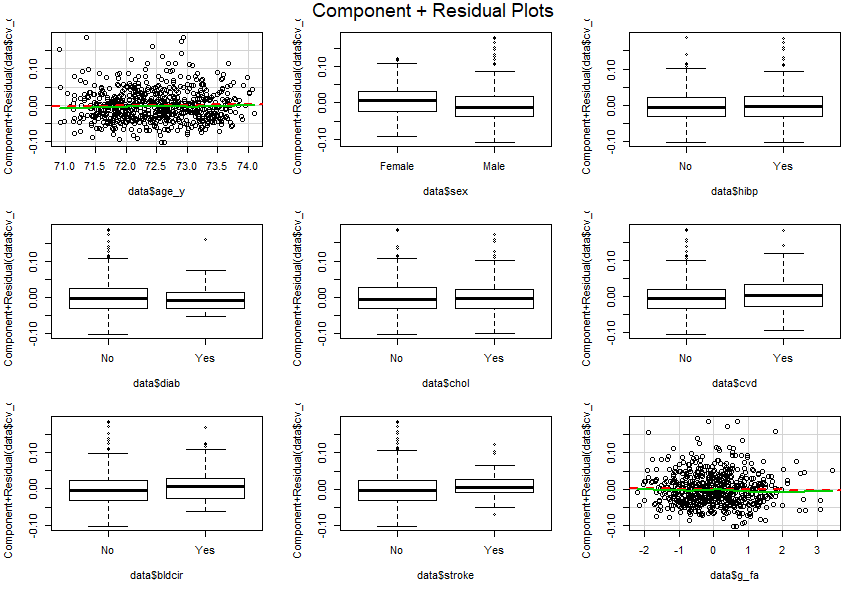


Table S13: Regression Model Results for Four-Choice Reaction Time CV on Average White Matter Tract Mean Diffusivity (n=647)

|  |  | **Model 1** |  |
| --- | --- | --- | --- |
|  | **b** | ***se*** | ***p*-value** |
| Age | 0.001 | 0.002 | 0.624 |
| Sex | -0.015 | 0.004 | <.001 |
|  |  |  |  |
| Hypertension | 0.000 | 0.004 | 0.896 |
| Diabetes | -0.001 | 0.006 | 0.805 |
| Cholesterol | 0.000 | 0.004 | 0.909 |
| CVD | 0.009 | 0.004 | 0.028 |
| Blood Circulation | 0.009 | 0.005 | 0.046 |
| Stroke | 0.011 | 0.007 | 0.107 |
|  |  |  |  |
| gMD | -0.001 | 0.002 | 0.736 |
| F | 3.601 | (9, 638) | <.001 |
| R-square | 0.048 |  |  |
| Adjusted R-square | 0.035 |  |  |

*Notes:* WMT gMD = white matter tract general mean diffusivity factor; CVD = cardiovascular disease; CRT = choice reaction time.

***Assumptions***

Max VIF = 1.19


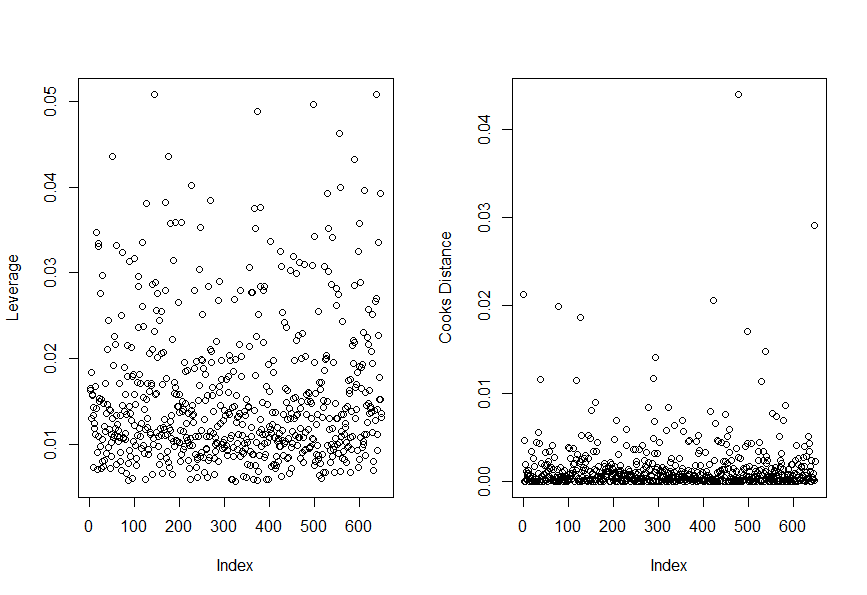


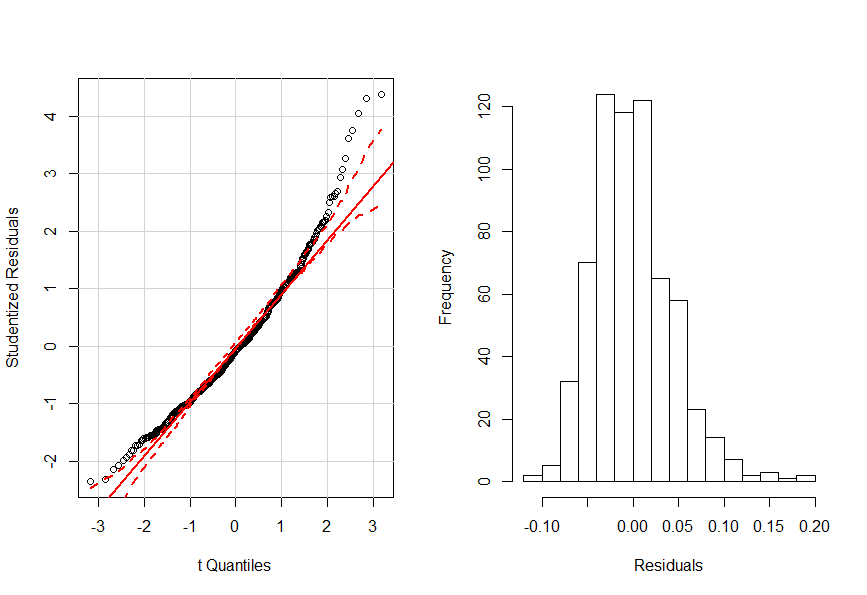


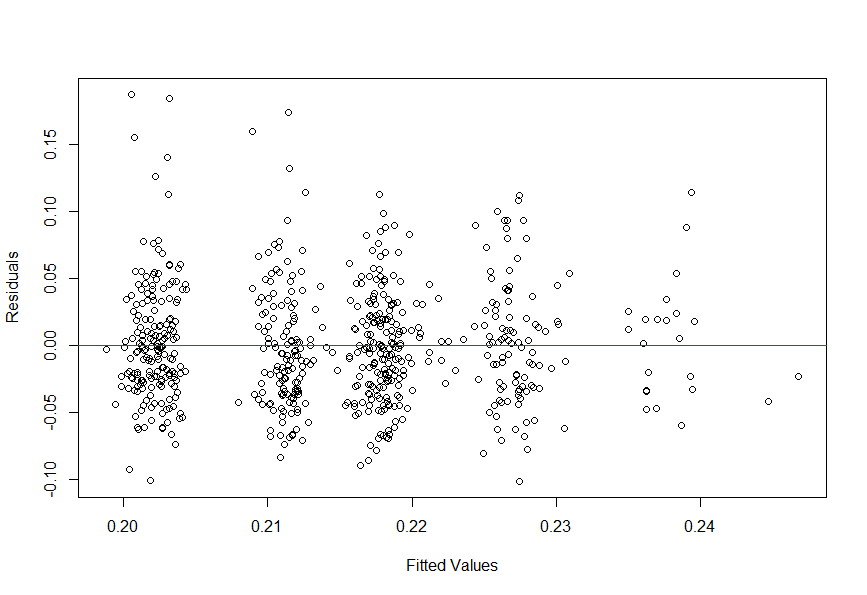


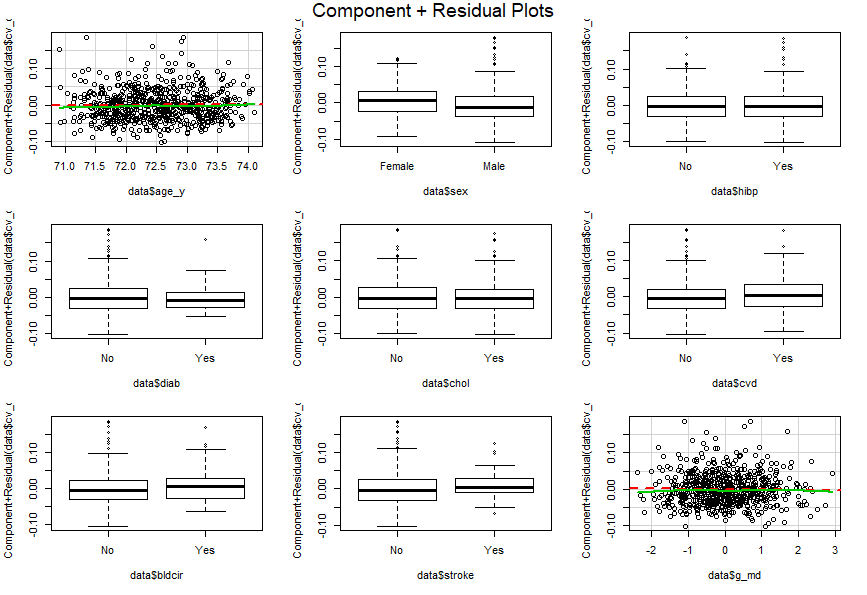


**Individual White Matter Tract Models**

Table S14: Regression Model Results for Four-Choice Reaction Time Standard Deviation and Individual White Matter Tract Fractional Anisotropy (n=358)

|  |  | **Model 1** |  |  | **Model 2** |  |
| --- | --- | --- | --- | --- | --- | --- |
|  | **b** | ***se*** | ***p*-value** | **b** | ***se*** | ***p*-value** |
| Age | 3.617 | 2.872 | 0.209 | 0.197 | 2.336 | 0.933 |
| Sex | -5.732 | 4.346 | 0.188 | -7.282 | 3.516 | 0.039 |
|  |  |  |  |  |  |  |
| Hypertension | -0.966 | 4.327 | 0.824 | 1.195 | 3.503 | 0.733 |
| Diabetes | 0.003 | 6.979 | 1.000 | -1.828 | 5.645 | 0.746 |
| Cholesterol | 2.697 | 4.304 | 0.531 | -0.074 | 3.486 | 0.983 |
| CVD | 3.960 | 4.738 | 0.404 | 2.520 | 3.832 | 0.511 |
| Blood Circulation | 10.346 | 5.191 | 0.047 | 8.793 | 4.199 | 0.037 |
| Stroke | 10.326 | 8.850 | 0.244 | 6.336 | 7.162 | 0.377 |
|  |  |  |  |  |  |  |
| Genu Corpus Callosum | 126.023 | 56.589 | 0.027 | 37.200 | 46.227 | 0.422 |
| Splenium Corpus Callosum | 15.287 | 32.386 | 0.637 | 37.398 | 26.238 | 0.155 |
| Arcuate Fasciculus | 18.203 | 71.520 | 0.799 | 29.730 | 57.836 | 0.608 |
| Anterior Thalamic Radiation | -147.639 | 88.673 | 0.097 | -40.415 | 72.137 | 0.576 |
| Rostral Cingulum | -94.898 | 65.514 | 0.148 | -52.891 | 53.064 | 0.32 |
| Uncinate Fasciculus | 20.952 | 99.951 | 0.834 | 49.154 | 80.845 | 0.544 |
| Inferior Longitudinal Thalamic Radiation | 15.641 | 67.489 | 0.817 | 26.225 | 54.576 | 0.631 |
|  |  |  |  |  |  |  |
| CRT SD | - | - | - | 0.270 | 0.020 | <.001 |
| F | 1.724 | (15, 342) | 0.030 | 13.85 | (16, 341) | <.001 |
| R-square | 0.070 |  |  | 0.394 |  |  |
| Adjusted R-square | 0.030 |  |  | 0.366 |  |  |

***Assumptions***

Max VIF = 1.85


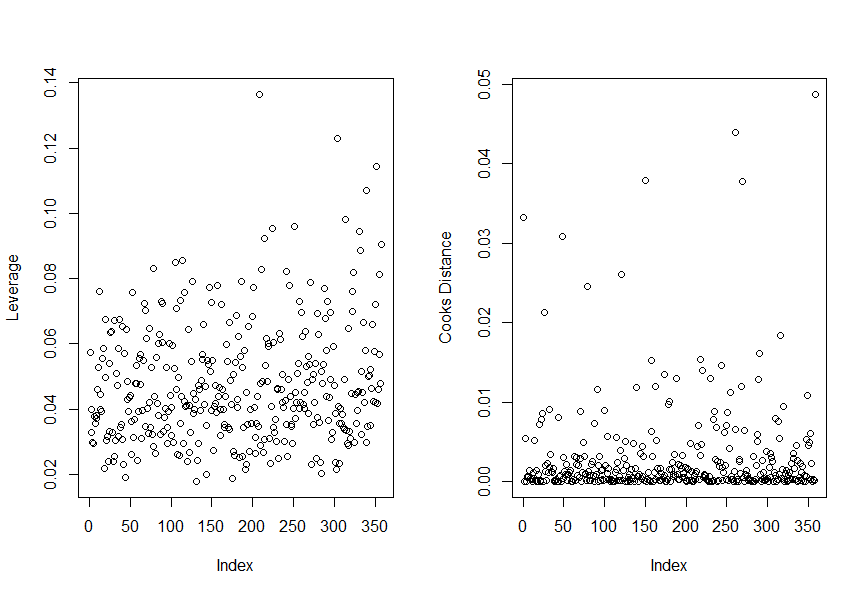


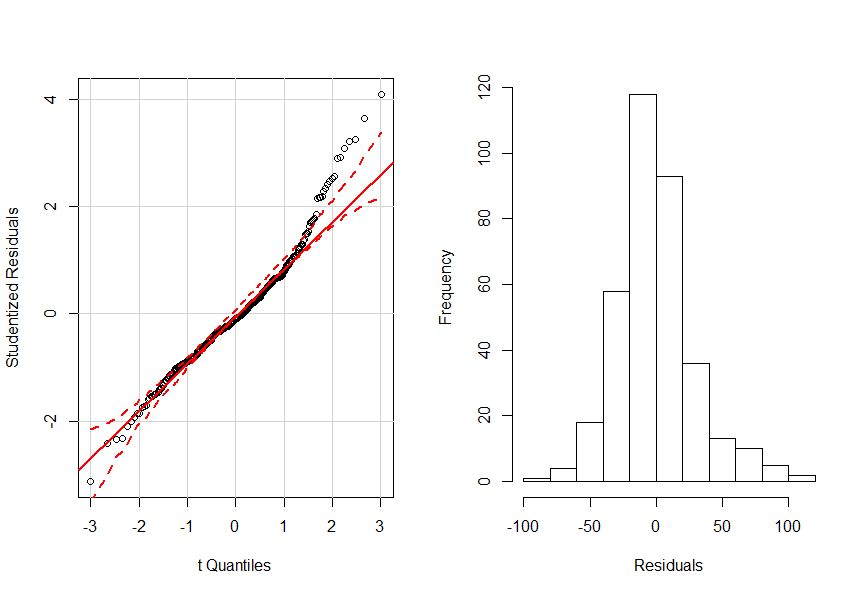


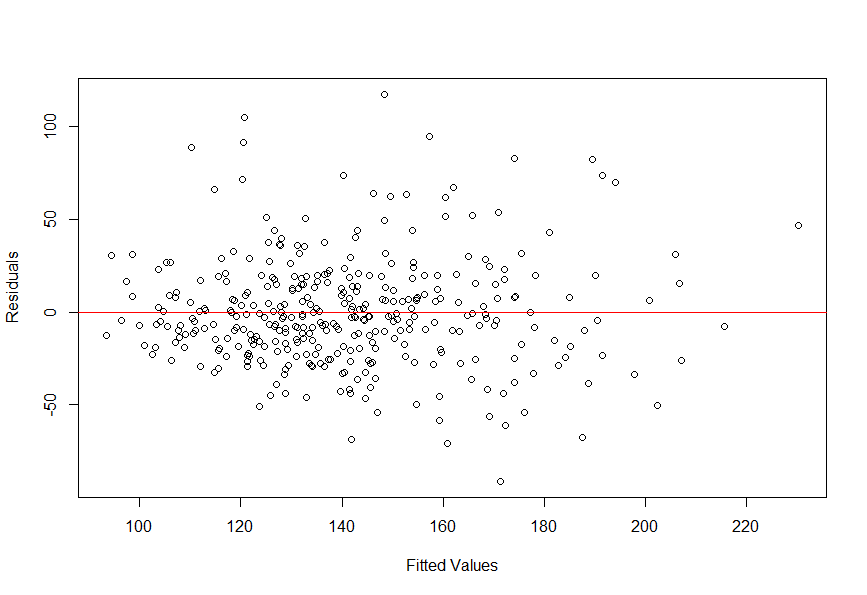


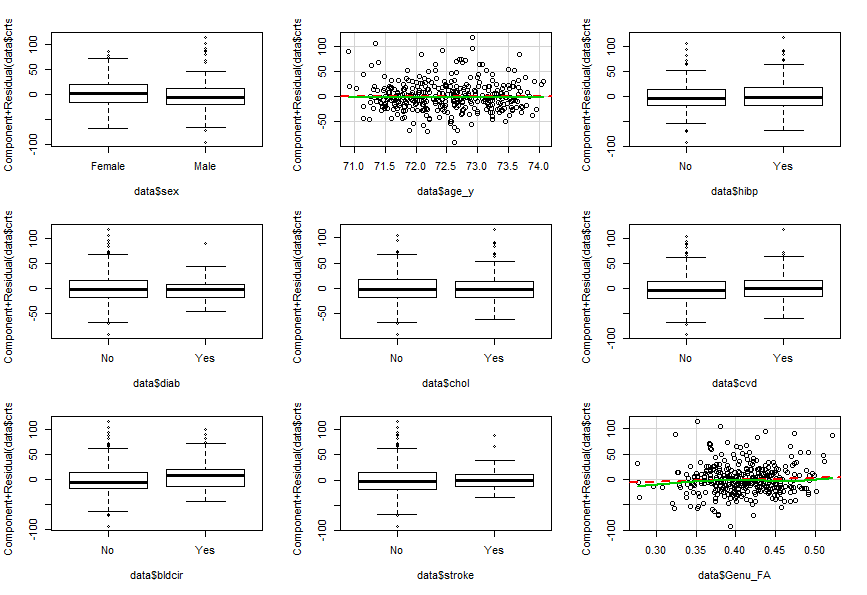


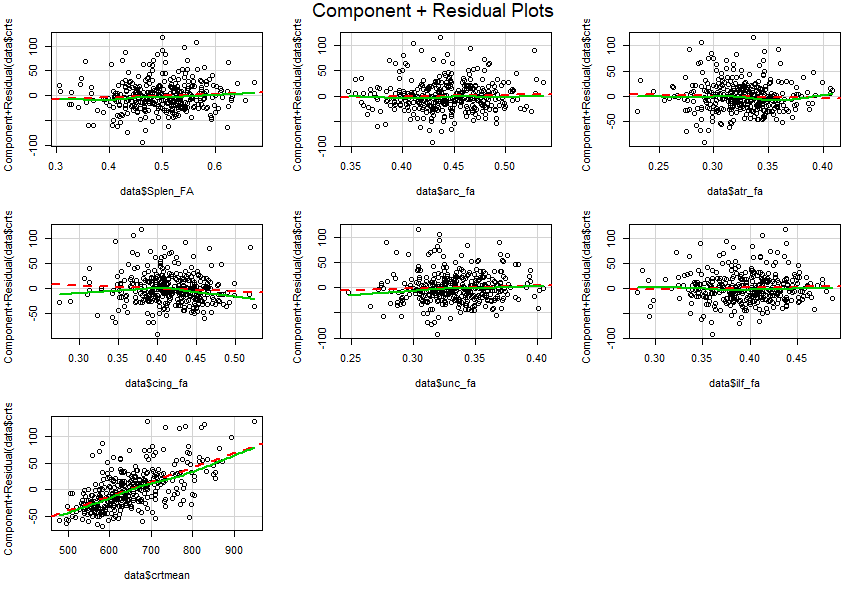


Table S15: Regression Model Results for Four-Choice Reaction Time Standard Deviation and Individual White Matter Tract Mean Diffusivity (n=358)

|  |  | **Model 1** |  |  | **Model 2** |  |
| --- | --- | --- | --- | --- | --- | --- |
|  | **b** | ***se*** | ***p*-value** | **b** | ***se*** | ***p*-value** |
| Age | 4.232 | 2.916 | 0.148 | 0.417 | 2.381 | 0.861 |
| Sex | -7.763 | 4.461 | 0.083 | -9.410 | 3.618 | 0.010 |
|  |  |  |  |  |  |  |
| Hypertension | -1.081 | 4.328 | 0.803 | 0.798 | 3.511 | 0.820 |
| Diabetes | 0.957 | 6.932 | 0.890 | -1.566 | 5.622 | 0.781 |
| Cholesterol | 2.428 | 4.300 | 0.573 | -0.103 | 3.491 | 0.976 |
| CVD | 4.168 | 4.749 | 0.381 | 2.028 | 3.853 | 0.599 |
| Blood Circulation | 9.711 | 5.181 | 0.062 | 8.503 | 4.201 | 0.044 |
| Stroke | 11.829 | 8.805 | 0.180 | 6.167 | 7.15 | 0.389 |
|  |  |  |  |  |  |  |
| Genu Corpus Callosum | -0.048 | 0.041 | 0.236 | 0.028 | 0.034 | 0.404 |
| Splenium Corpus Callosum | -0.006 | 0.013 | 0.653 | -0.009 | 0.011 | 0.404 |
| Arcuate Fasciculus | 0.034 | 0.067 | 0.608 | 0.004 | 0.054 | 0.935 |
| Anterior Thalamic Radiation | 0.118 | 0.050 | 0.018 | 0.021 | 0.041 | 0.604 |
| Rostral Cingulum | -0.070 | 0.067 | 0.296 | -0.045 | 0.055 | 0.408 |
| Uncinate Fasciculus | 0.007 | 0.061 | 0.914 | -0.023 | 0.05 | 0.638 |
| Inferior Longitudinal Thalamic Radiation | -0.028 | 0.034 | 0.413 | -0.020 | 0.028 | 0.466 |
|  |  |  |  |  |  |  |
| CRT SD | - | - | - | 0.271 | 0.02 | <.001 |
| F | 1.631 | (15, 342) | 0.064 | 13.54 | (16, 341) | <.001 |
| R-square | 0.067 |  |  | 0.389 |  |  |
| Adjusted R-square | 0.026 |  |  | 0.360 |  |  |

***Assumptions***

Max VIF = 2.03


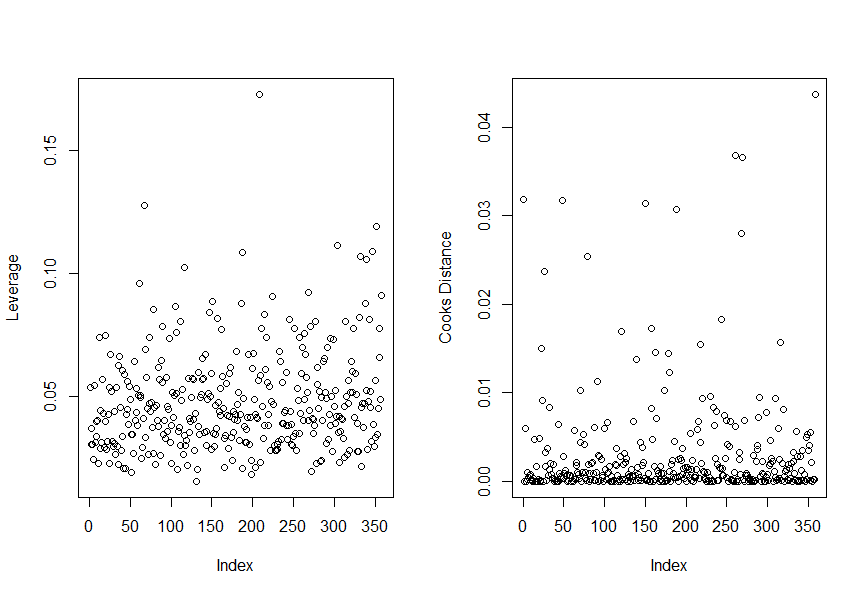


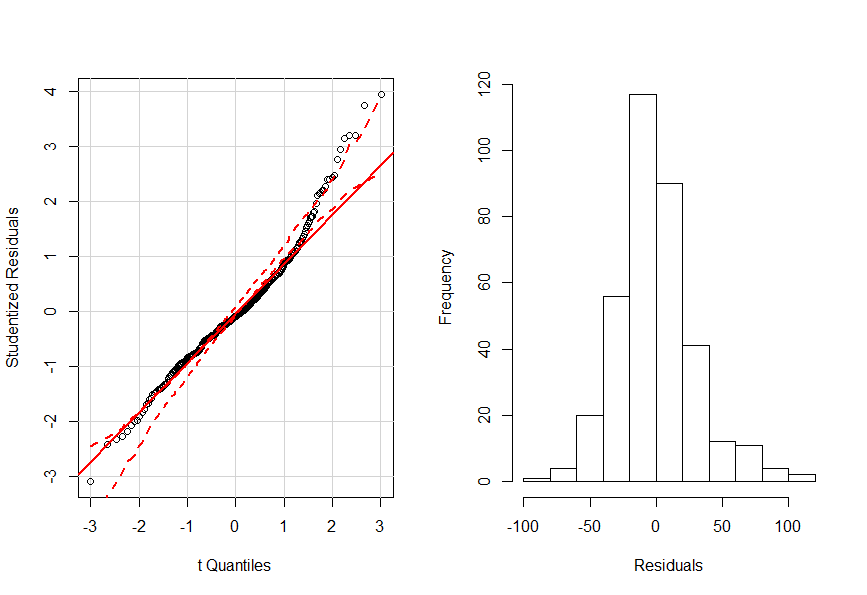


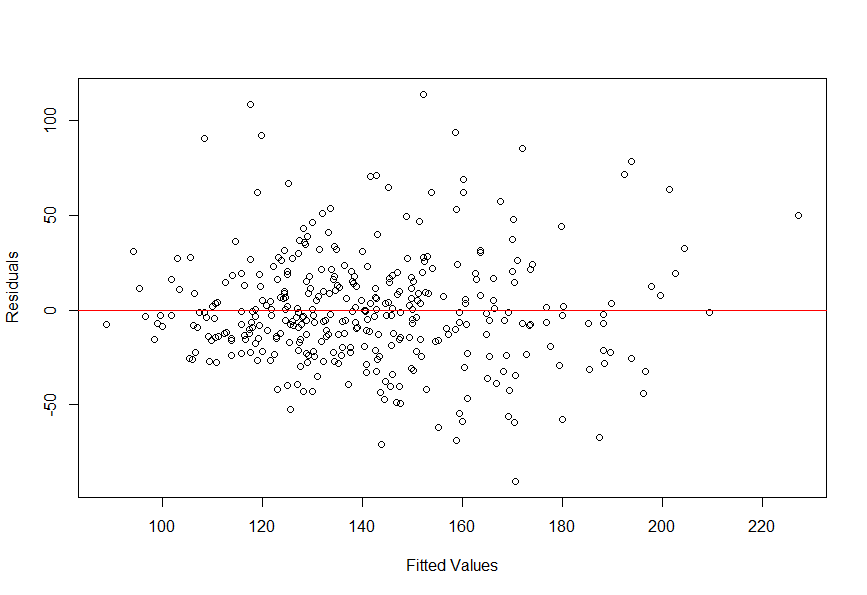


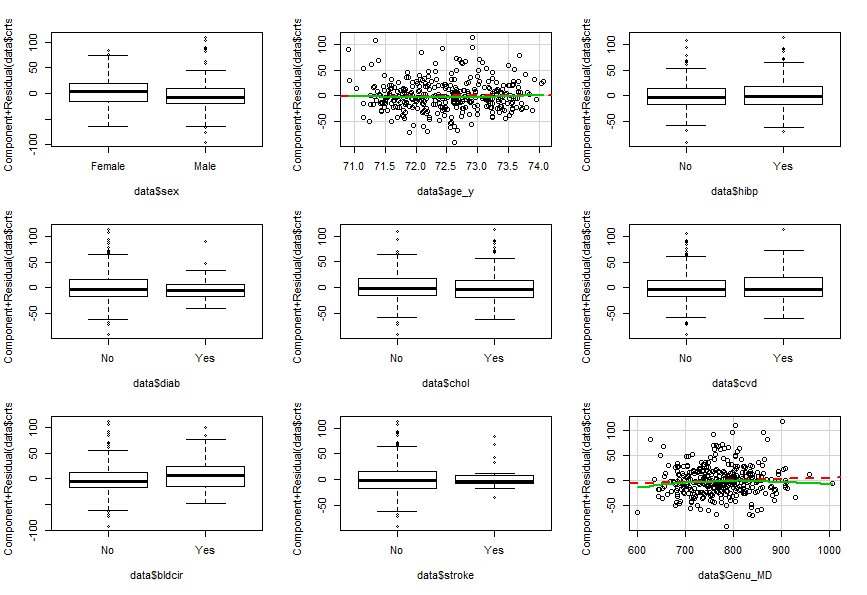


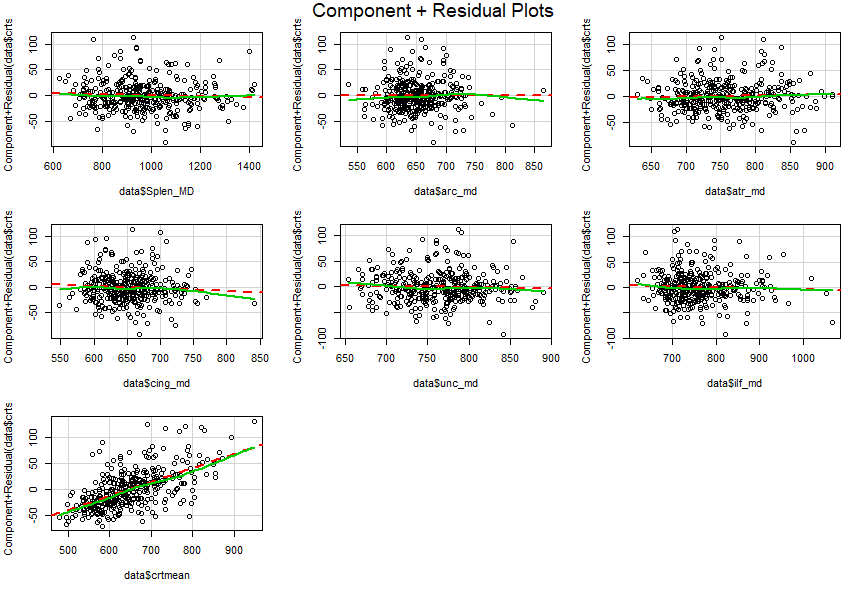


Table S16: Regression Model Results for Four-Choice Reaction Time Mean and Individual White Matter Tract Fractional Anisotropy (n=358)

|  |  | **Model 1** |  |  | **Model 2** |  |
| --- | --- | --- | --- | --- | --- | --- |
|  | **b** | ***se*** | ***p*-value** | **b** | ***se*** | ***p*-value** |
| Age | 12.686 | 6.284 | 0.044 | 8.016 | 5.093 | 0.116 |
| Sex | 5.750 | 9.511 | 0.546 | 13.151 | 7.710 | 0.089 |
|  |  |  |  |  |  |  |
| Hypertension | -8.014 | 9.470 | 0.398 | -6.767 | 7.658 | 0.377 |
| Diabetes | 6.792 | 15.273 | 0.657 | 6.789 | 12.349 | 0.583 |
| Cholesterol | 10.278 | 9.420 | 0.276 | 6.795 | 7.621 | 0.373 |
| CVD | 5.341 | 10.368 | 0.607 | 0.227 | 8.392 | 0.978 |
| Blood Circulation | 5.760 | 11.361 | 0.612 | -7.599 | 9.240 | 0.411 |
| Stroke | 14.799 | 19.367 | 0.445 | 1.466 | 15.691 | 0.926 |
|  |  |  |  |  |  |  |
| Genu Corpus Callosum | 329.459 | 123.842 | 0.008 | 166.735 | 100.859 | 0.099 |
| Splenium Corpus Callosum | -82.013 | 70.876 | 0.248 | -101.752 | 57.327 | 0.077 |
| Arcuate Fasciculus | -42.756 | 156.520 | 0.785 | -66.260 | 126.570 | 0.601 |
| Anterior Thalamic Radiation | -397.711 | 194.057 | 0.041 | -207.076 | 157.544 | 0.190 |
| Rostral Cingulum | -155.810 | 143.375 | 0.278 | -33.276 | 116.284 | 0.775 |
| Uncinate Fasciculus | -104.604 | 218.738 | 0.633 | -131.658 | 176.878 | 0.457 |
| Inferior Longitudinal Thalamic Radiation | -39.258 | 147.698 | 0.791 | -59.455 | 119.434 | 0.619 |
|  |  |  |  |  |  |  |
| CRT SD | - | - | - | 1.291 | 0.096 | <.001 |
| F | 1.83 | (15, 342) | 0.034 | 14.01 | (16, 341) | <.001 |
| R-square | 0.074 |  |  | 0.397 |  |  |
| Adjusted R-square | 0.034 |  |  | 0.368 |  |  |

***Assumptions***

Max VIF = 1.85


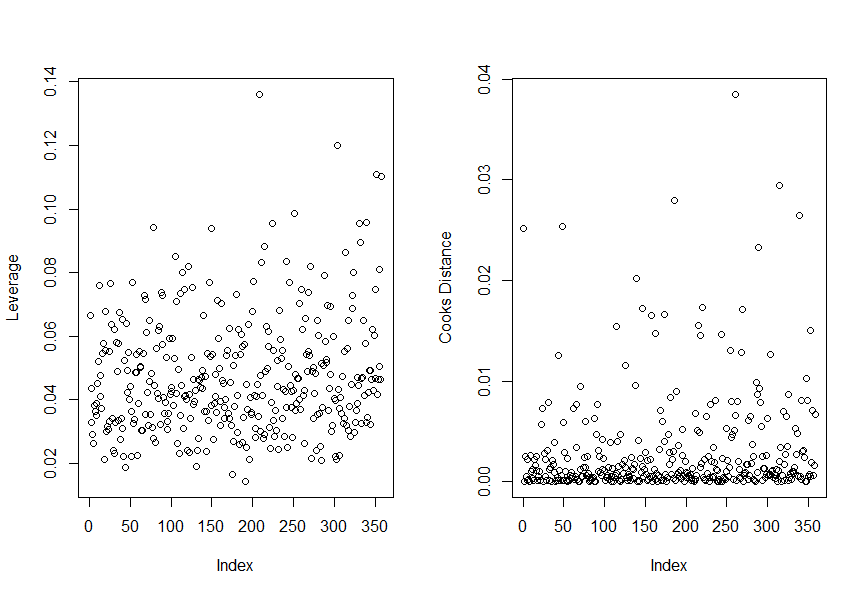


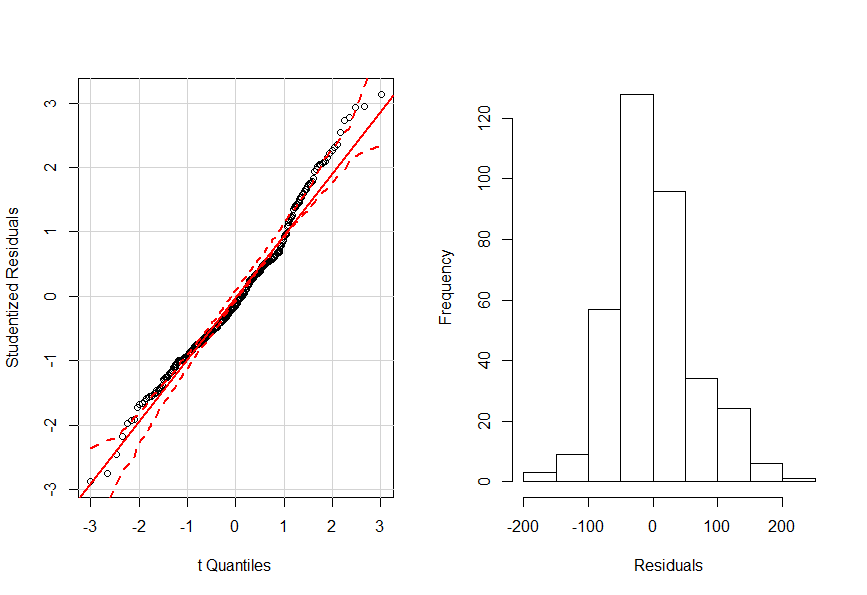


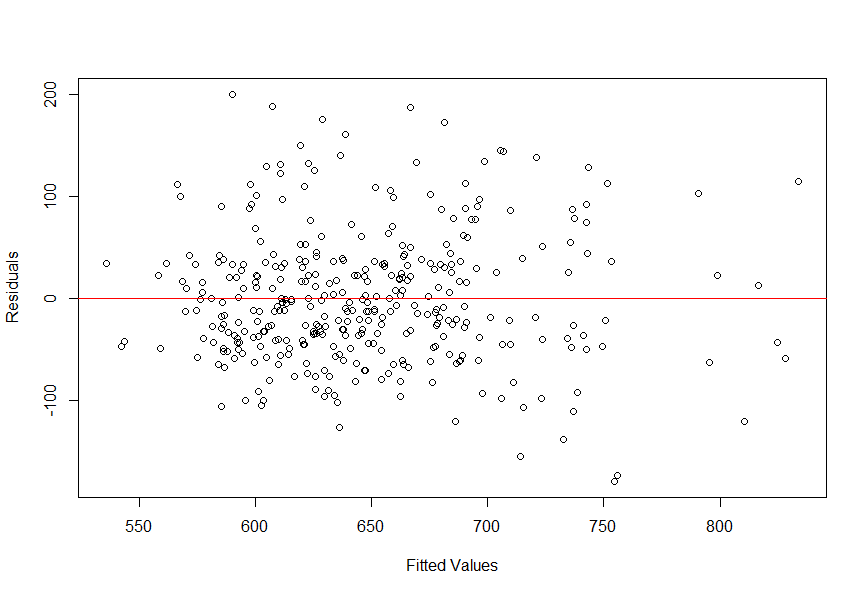


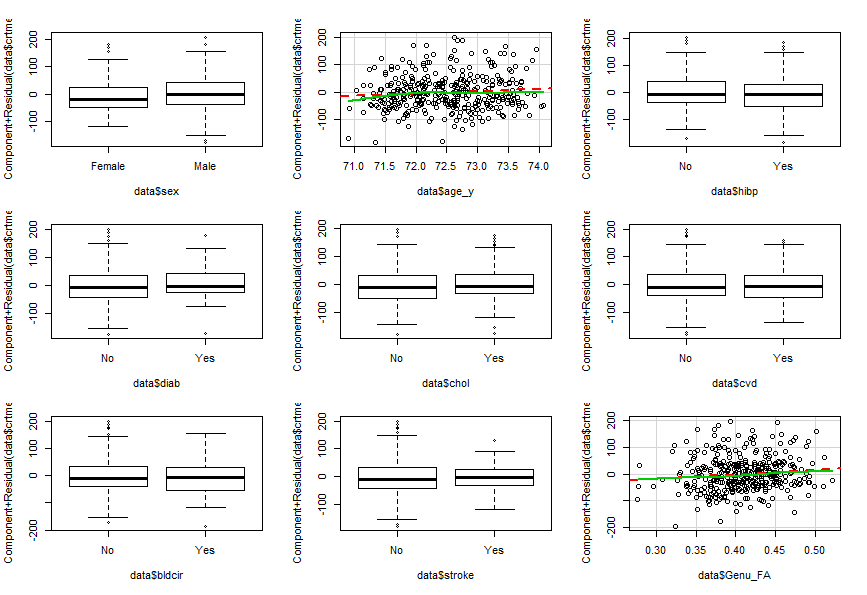


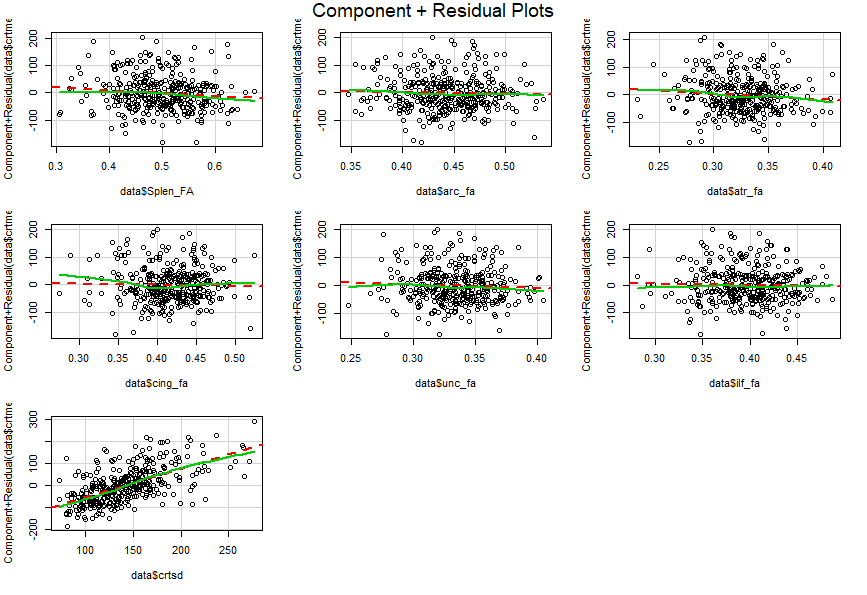


Table S17: Regression Model Results for Four-Choice Reaction Time Mean and Individual White Matter Tract Mean Diffusivity (n=358)

|  |  | **Model 1** |  |  | **Model 2** |  |
| --- | --- | --- | --- | --- | --- | --- |
|  | **b** | ***se*** | ***p*-value** | **b** | ***se*** | ***p*-value** |
| Age | 6.083 | 9.673 | 0.530 | 15.967 | 7.876 | 0.043 |
| Sex | 14.087 | 6.323 | 0.027 | 8.700 | 5.142 | 0.092 |
|  |  |  |  |  |  |  |
| Hypertension | -6.938 | 9.385 | 0.460 | -5.562 | 7.609 | 0.465 |
| Diabetes | 9.315 | 15.031 | 0.536 | 8.097 | 12.185 | 0.507 |
| Cholesterol | 9.348 | 9.325 | 0.317 | 6.256 | 7.563 | 0.409 |
| CVD | 7.905 | 10.299 | 0.443 | 2.598 | 8.358 | 0.756 |
| Blood Circulation | 4.460 | 11.234 | 0.692 | -7.904 | 9.154 | 0.388 |
| Stroke | 20.912 | 19.092 | 0.274 | 5.852 | 15.519 | 0.706 |
|  |  |  |  |  |  |  |
| Genu Corpus Callosum | -0.282 | 0.088 | 0.002 | -0.221 | 0.072 | 0.002 |
| Splenium Corpus Callosum | 0.011 | 0.028 | 0.698 | 0.018 | 0.023 | 0.422 |
| Arcuate Fasciculus | 0.110 | 0.144 | 0.447 | 0.066 | 0.117 | 0.571 |
| Anterior Thalamic Radiation | 0.356 | 0.107 | 0.001 | 0.206 | 0.088 | 0.019 |
| Rostral Cingulum | -0.093 | 0.146 | 0.524 | -0.003 | 0.118 | 0.977 |
| Uncinate Fasciculus | 0.111 | 0.133 | 0.405 | 0.102 | 0.108 | 0.342 |
| Inferior Longitudinal Thalamic Radiation | -0.029 | 0.075 | 0.697 | 0.007 | 0.061 | 0.910 |
|  |  |  |  |  |  |  |
| CRT SD | - | - | - | 1.273 | 0.095 | <.001 |
| F | 2.192 | (15, 342) | 0.007 | 14.34 | (16, 341) | <.001 |
| R-square | 0.088 |  |  | 0.402 |  |  |
| Adjusted R-square | 0.048 |  |  | 0.374 |  |  |

***Assumptions***

Max VIF = 2.02


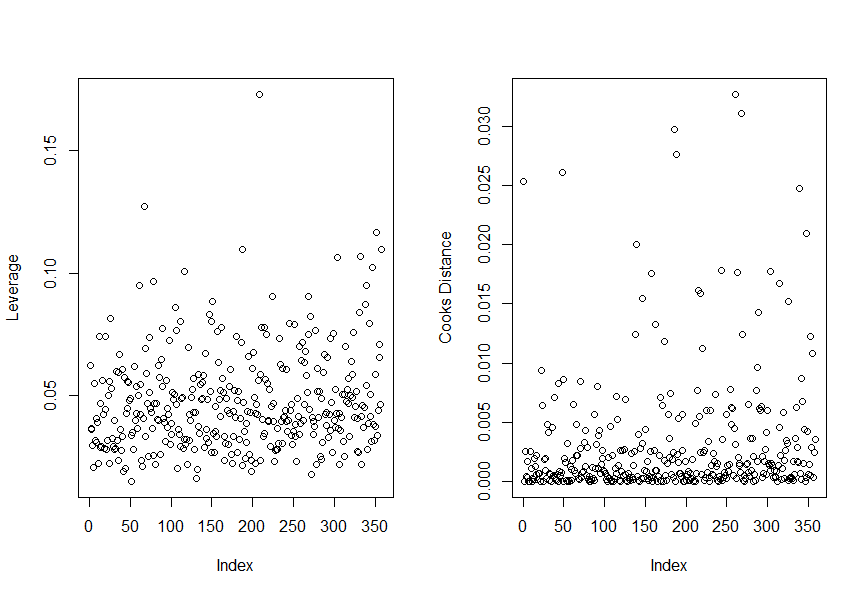


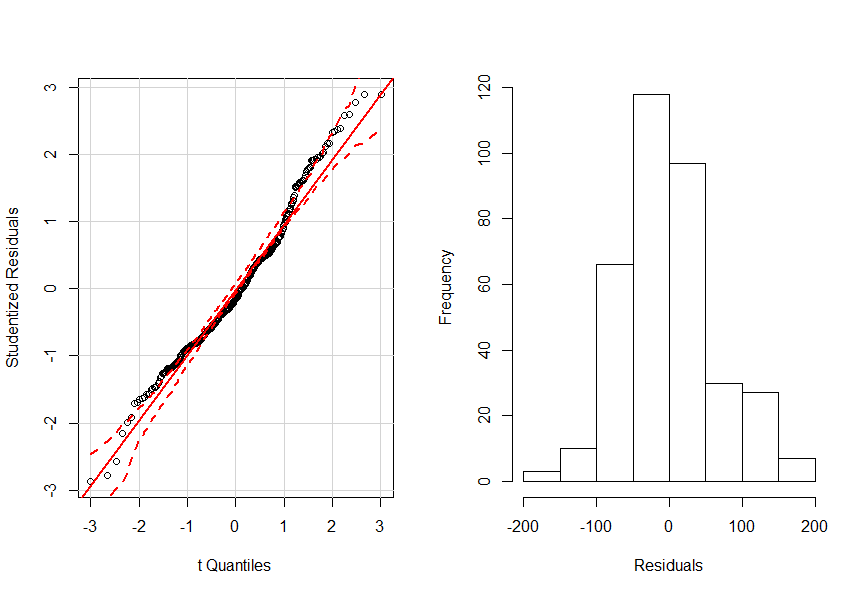


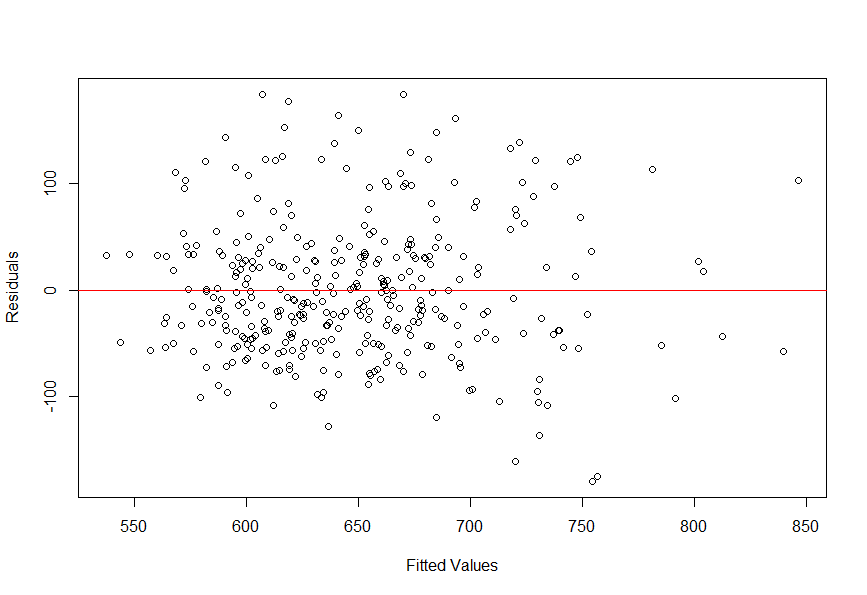


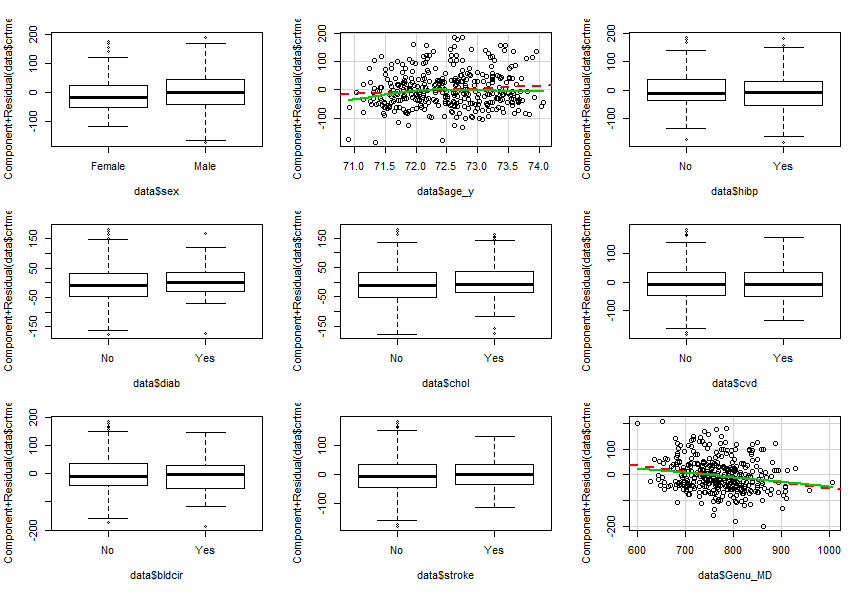


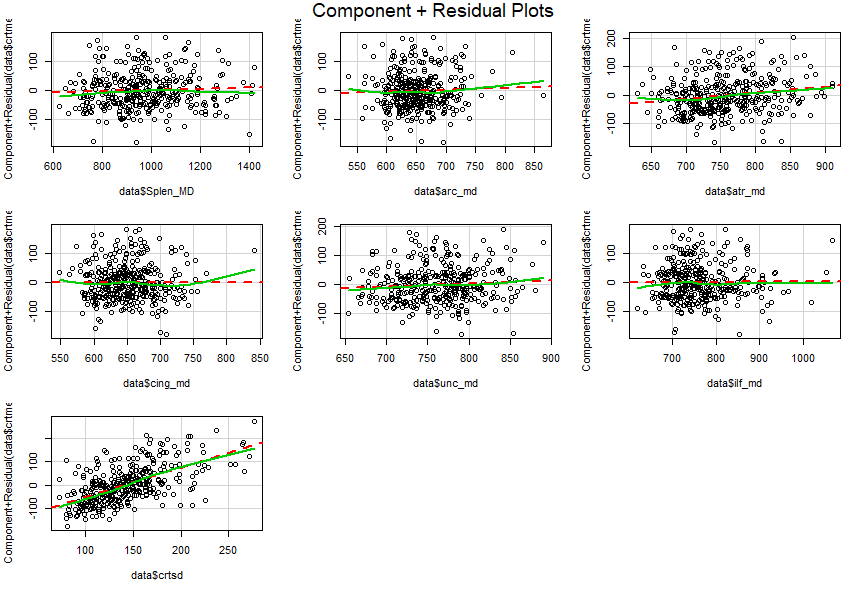


Table S18: Regression Model Results for Four-Choice Reaction Time CV and Individual White Matter Tract Fractional Anisotropy and Mean Diffusivity (n=358)

|  |  | **FA** |  |  | **MD** |  |
| --- | --- | --- | --- | --- | --- | --- |
|  | **b** | ***se*** | ***p*-value** | **b** | ***se*** | ***p*-value** |
| Age | 0.001 | 0.004 | 0.821 | 0.001 | 0.004 | 0.708 |
| Sex | -0.011 | 0.005 | 0.046 | -0.014 | 0.006 | 0.012 |
|  |  |  |  |  |  |  |
| Hypertension | 0.002 | 0.005 | 0.767 | 0.001 | 0.005 | 0.842 |
| Diabetes | -0.001 | 0.009 | 0.862 | -0.001 | 0.009 | 0.918 |
| Cholesterol | 0.000 | 0.005 | 0.998 | 0.000 | 0.005 | 0.981 |
| CVD | 0.003 | 0.006 | 0.553 | 0.003 | 0.006 | 0.588 |
| Blood Circulation | 0.013 | 0.006 | 0.044 | 0.012 | 0.006 | 0.054 |
| Stroke | 0.010 | 0.011 | 0.340 | 0.011 | 0.011 | 0.330 |
|  |  |  |  |  |  |  |
| Genu Corpus Callosum | 0.066 | 0.070 | 0.349 | 0.000 | 0.000 | 0.655 |
| Splenium Corpus Callosum | 0.051 | 0.040 | 0.198 | 0.000 | 0.000 | 0.472 |
| Arcuate Fasciculus | 0.063 | 0.088 | 0.476 | 0.000 | 0.000 | 0.849 |
| Anterior Thalamic Radiation | -0.083 | 0.109 | 0.447 | 0.000 | 0.000 | 0.316 |
| Rostral Cingulum | -0.100 | 0.081 | 0.217 | 0.000 | 0.000 | 0.385 |
| Uncinate Fasciculus | 0.070 | 0.123 | 0.573 | 0.000 | 0.000 | 0.777 |
| Inferior Longitudinal Thalamic Radiation | 0.020 | 0.083 | 0.812 | 0.000 | 0.000 | 0.441 |
| F | 1.436 | (15, 342) | 0.128 | 1.221 | (15, 342) | 0.253 |
| R-square | 0.059 |  |  | 0.051 |  |  |
| Adjusted R-square | 0.018 |  |  | 0.009 |  |  |

***Assumptions – FA model***

Max VIF = 1.85


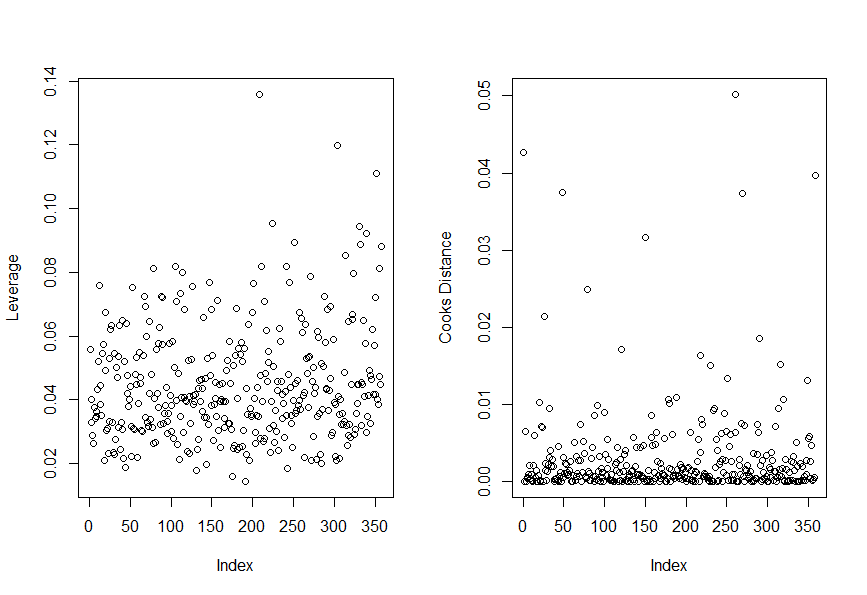


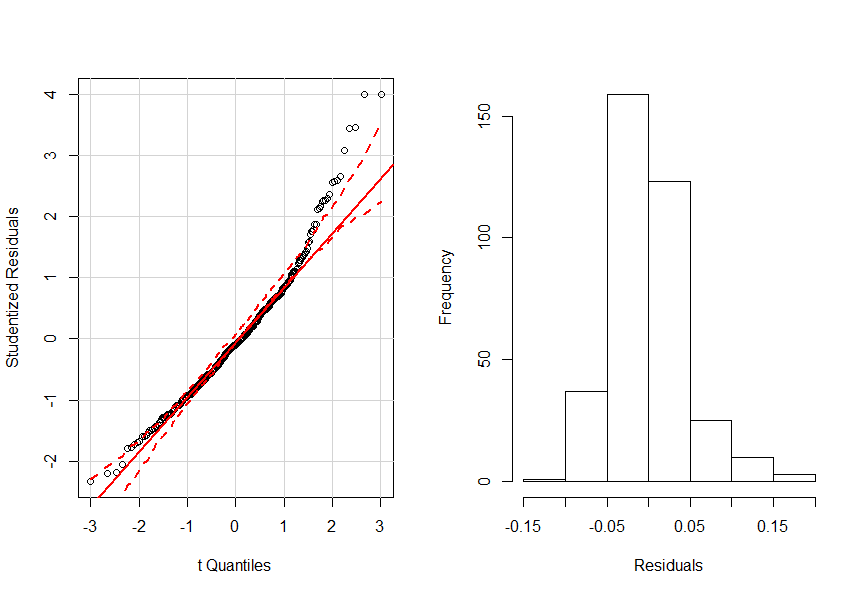


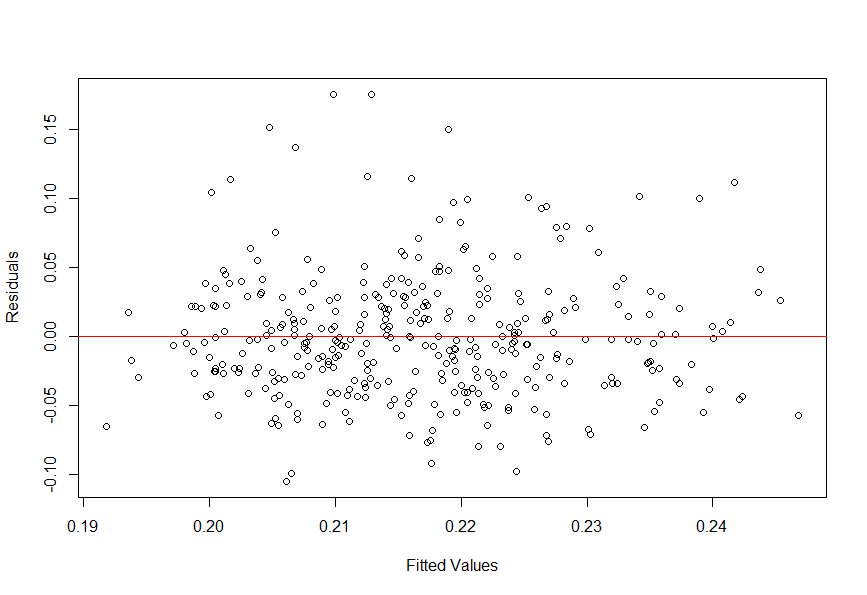


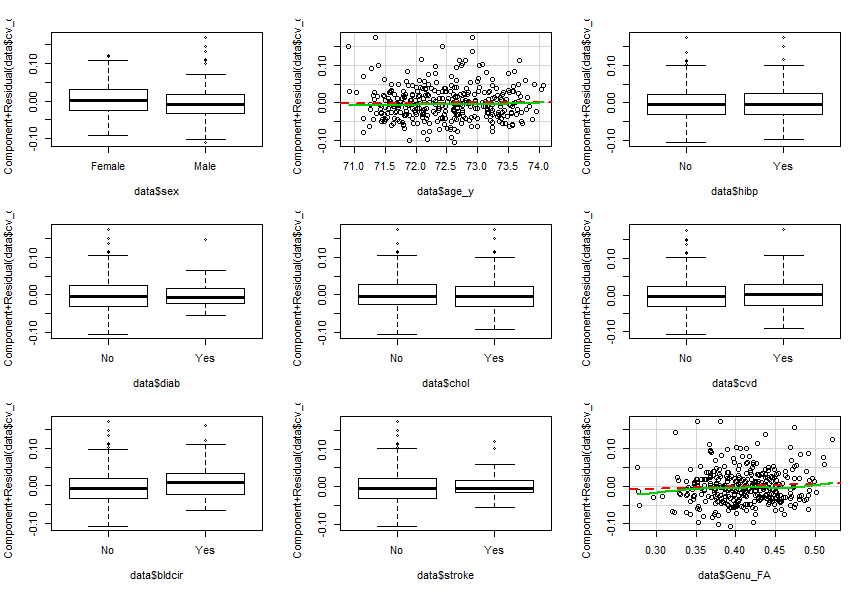


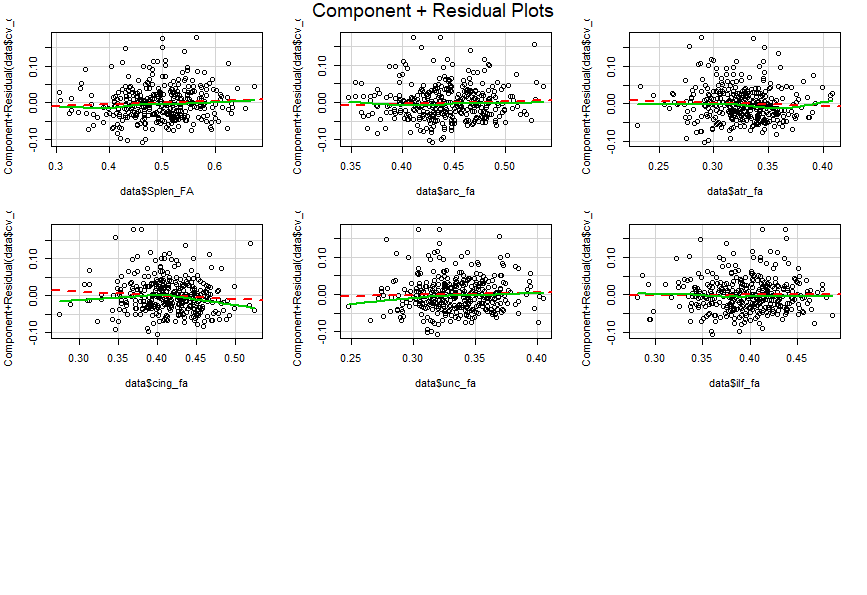
***Assumptions – MD Model***

Max VIF = 2.02


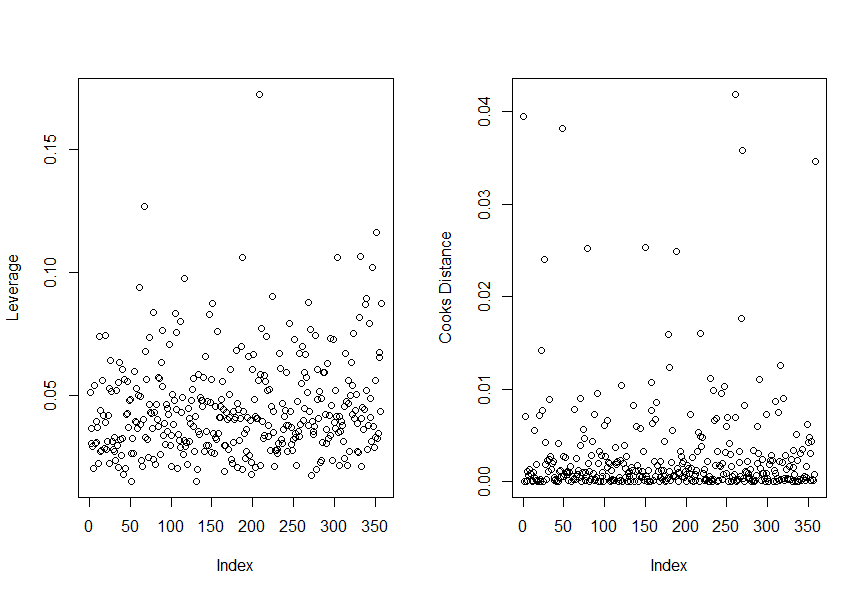


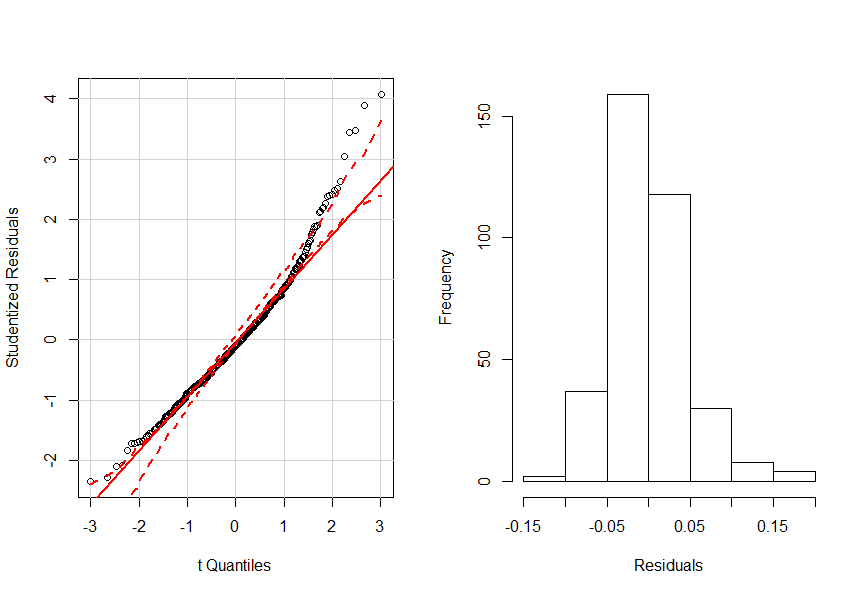


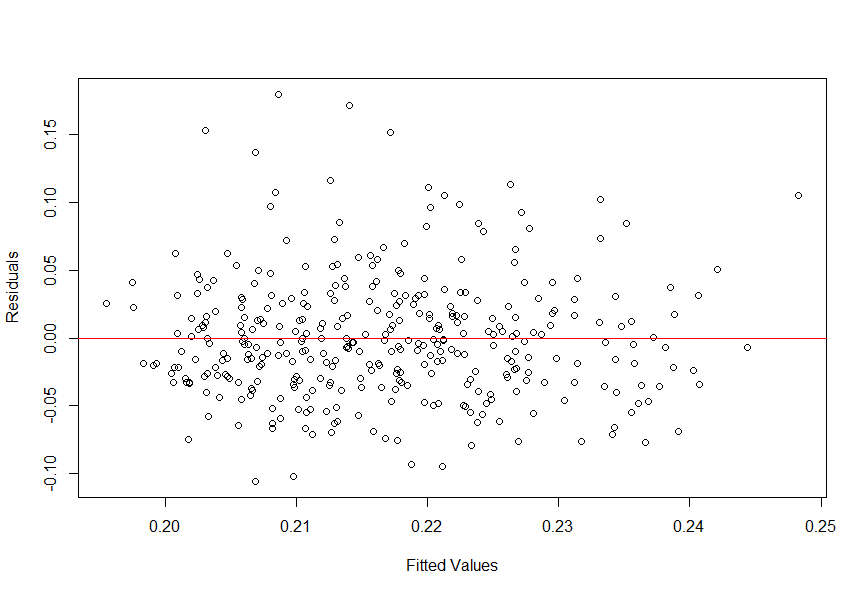


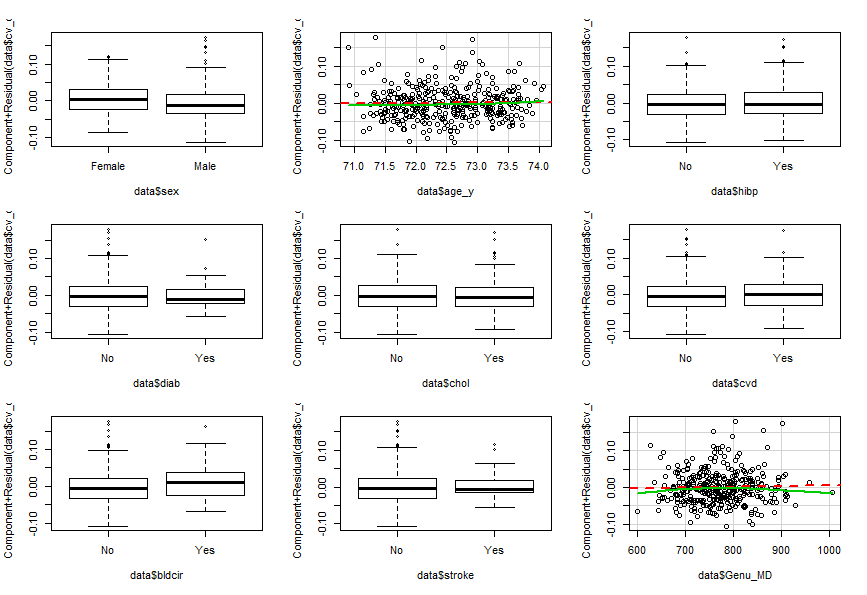


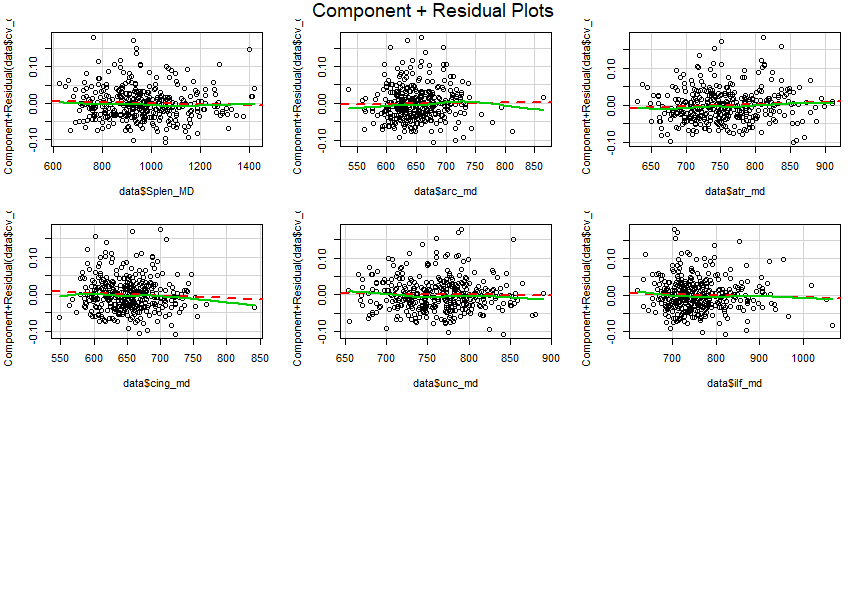


**Robust regression**

Table S19: Robust regression results for Choice Reaction Time Standard Deviation for the primary imaging predictors

|  | Value | SE | t-value | Value | SE | t-value | Value | SE | t-value | Value | SE | t-value |
| --- | --- | --- | --- | --- | --- | --- | --- | --- | --- | --- | --- | --- |
| *Predictors* |  |  |  |  |  |  |  |  |  |  |  |  |
| Sex | -10.797 | 2.085 | -5.178 | -0.189 | 1.438 | -0.132 | -10.807 | 2.136 | -5.060 | -10.84 | 2.130 | -5.090 |
| Age (Years) | -0.268 | 1.466 | -0.183 | -10.968 | 2.059 | -5.328 | 0.007 | 1.504 | 0.005 | -0.019 | 1.505 | -0.013 |
| Hypertension | 0.060 | 2.167 | 0.027 | 0.474 | 2.142 | 0.221 | 0.102 | 2.205 | 0.046 | -0.0800 | 2.184 | -0.037 |
| Diabetes | -2.284 | 3.462 | -0.660 | -3.022 | 3.428 | -0.882 | -2.435 | 3.562 | -0.684 | -2.35 | 3.551 | -0.662 |
| Cholesterol | -0.126 | 2.244 | -0.056 | -0.024 | 2.219 | -0.011 | -0.105 | 2.293 | -0.046 | -0.026 | 2.282 | -0.011 |
| CVD | 5.256 | 2.388 | 2.201 | 5.675 | 2.362 | 2.403 | 5.801 | 2.432 | 2.386 | 5.717 | 2.422 | 2.361 |
| Blood Circulation | 3.453 | 2.724 | 1.267 | 3.661 | 2.700 | 1.356 | 4.038 | 2.778 | 1.454 | 4.056 | 2.768 | 1.465 |
| Stroke | 5.601 | 4.114 | 1.361 | 5.396 | 4.110 | 1.313 | 6.135 | 4.132 | 1.485 | 5.979 | 4.113 | 1.454 |
| CRT Mean | 0.269 | 0.012 | 22.195 | 0.270 | 0.012 | 22.431 | 0.271 | 0.012 | 21.985 | 0.270 | 0.012 | 22.165 |
|  |  |  |  |  |  |  |  |  |  |  |  |  |
| WMH Volume | 0.567 | 1.048 | 0.541 |  |  |  |  |  |  |  |  |  |
| Wahlund: Frontal |  |  |  | 4.777 | 2.881 | 1.658 |  |  |  |  |  |  |
| Wahlund: Parieto-Occipital |  |  |  | -5.577 | 2.762 | -2.019 |  |  |  |  |  |  |
| Wahlund: Basal Ganglia |  |  |  | -3.750 | 5.974 | -0.628 |  |  |  |  |  |  |
| Wahlund: Temporal |  |  |  | -1.880 | 9.801 | -0.192 |  |  |  |  |  |  |
| Wahlund: Infratentorial |  |  |  | 6.712 | 7.362 | 0.912 |  |  |  |  |  |  |
| gFA |  |  |  |  |  |  | -1.204 | 1.163 | -1.035 |  |  |  |
| gMD |  |  |  |  |  |  |  |  |  | -0.761 | 1.136 | -0.670 |

Table S20: Robust regression results for Choice Reaction Time Mean for the primary imaging predictors

|  | Value | SE | t-value | Value | SE | t-value | Value | SE | t-value | Value | SE | t-value |
| --- | --- | --- | --- | --- | --- | --- | --- | --- | --- | --- | --- | --- |
| *Predictors* |  |  |  |  |  |  |  |  |  |  |  |  |
| Sex | 20.287 | 5.027 | 4.035 | 20.205 | 5.108 | 3.956 | 20.796 | 5.194 | 4.004 | 20.989 | 5.208 | 4.031 |
| Age (Years) | 6.716 | 3.497 | 1.920 | 7.511 | 3.527 | 2.130 | 6.787 | 3.619 | 1.875 | 6.945 | 3.643 | 1.906 |
| Hypertension | -1.182 | 5.192 | -0.228 | -1.201 | 5.281 | -0.227 | -2.066 | 5.333 | -0.388 | -0.25 | 5.313 | -0.047 |
| Diabetes | 17.985 | 8.279 | 2.172 | 18.346 | 8.435 | 2.175 | 18.490 | 8.595 | 2.151 | 17.37 | 8.620 | 2.015 |
| Cholesterol | 5.299 | 5.373 | 0.986 | 5.141 | 5.468 | 0.940 | 6.843 | 5.538 | 1.235 | 6.168 | 5.544 | 1.112 |
| CVD | -3.541 | 5.738 | -0.617 | -3.579 | 5.843 | -0.613 | -6.476 | 5.895 | -1.098 | -5.917 | 5.906 | -1.002 |
| Blood Circulation | -0.627 | 6.544 | -0.096 | -0.551 | 6.676 | -0.082 | -3.398 | 6.735 | -0.504 | -3.415 | 6.750 | -0.506 |
| Stroke | -3.917 | 9.874 | -0.397 | -3.298 | 10.153 | -0.325 | -4.134 | 10.011 | -0.413 | -3.195 | 10.024 | -0.319 |
| CRT SD | 1.499 | 0.068 | 22.068 | 1.495 | 0.069 | 21.599 | 1.501 | 0.069 | 21.711 | 1.506 | 0.069 | 21.768 |
|  |  |  |  |  |  |  |  |  |  |  |  |  |
| WMH Volume | 5.345 | 2.501 | 2.137 |  |  |  |  |  |  |  |  |  |
| Wahlund: Frontal |  |  |  | 6.173 | 7.108 | 0.869 |  |  |  |  |  |  |
| Wahlund: Parieto-Occipital |  |  |  | 9.929 | 6.822 | 1.456 |  |  |  |  |  |  |
| Wahlund: Basal Ganglia |  |  |  | -13.698 | 14.722 | -0.93 |  |  |  |  |  |  |
| Wahlund: Temporal |  |  |  | 33.373 | 24.146 | 1.382 |  |  |  |  |  |  |
| Wahlund: Infratentorial |  |  |  | 19.424 | 18.158 | 1.07 |  |  |  |  |  |  |
| gFA |  |  |  |  |  |  | 9.847 | 2.784 | 3.537 |  |  |  |
| gMD |  |  |  |  |  |  |  |  |  | 5.474 | 2.755 | 1.987 |

Table S21: Robust regression results for Choice Reaction Time Coefficient of Variation for the primary imaging predictors

|  | Value | SE | t-value | Value | SE | t-value | Value | SE | t-value | Value | SE | t-value |
| --- | --- | --- | --- | --- | --- | --- | --- | --- | --- | --- | --- | --- |
| *Predictors* |  |  |  |  |  |  |  |  |  |  |  |  |
| Sex | -0.017 | 0.003 | -5.060 | -0.017 | 0.003 | -5.133 | -0.017 | 0.003 | -4.897 | -0.017 | 0.003 | -4.893 |
| Age (Years) | 0.001 | 0.002 | 0.308 | 0.001 | 0.002 | 0.425 | 0.001 | 0.002 | 0.536 | 0.001 | 0.002 | 0.519 |
| Hypertension | -0.001 | 0.003 | -0.205 | 0.000 | 0.003 | -0.002 | -0.001 | 0.004 | -0.175 | -0.001 | 0.004 | -0.221 |
| Diabetes | -0.001 | 0.005 | -0.245 | -0.002 | 0.006 | -0.431 | -0.001 | 0.006 | -0.263 | -0.001 | 0.006 | -0.245 |
| Cholesterol | 0.000 | 0.004 | 0.037 | 0.000 | 0.004 | 0.034 | 0.000 | 0.004 | 0.103 | 0.000 | 0.004 | 0.112 |
| CVD | 0.009 | 0.004 | 2.332 | 0.010 | 0.004 | 2.548 | 0.009 | 0.004 | 2.436 | 0.009 | 0.004 | 2.423 |
| Blood Circulation | 0.007 | 0.004 | 1.564 | 0.007 | 0.004 | 1.661 | 0.008 | 0.004 | 1.699 | 0.008 | 0.004 | 1.705 |
| Stroke | 0.010 | 0.007 | 1.597 | 0.010 | 0.007 | 1.539 | 0.011 | 0.007 | 1.731 | 0.011 | 0.007 | 1.703 |
|  |  |  |  |  |  |  |  |  |  |  |  |  |
| WMH Volume | 0.002 | 0.002 | 1.049 |  |  |  |  |  |  |  |  |  |
| Wahlund: Frontal |  |  |  | 0.009 | 0.005 | 2.019 |  |  |  |  |  |  |
| Wahlund: Parieto-Occipital |  |  |  | -0.009 | 0.004 | -1.948 |  |  |  |  |  |  |
| Wahlund: Basal Ganglia |  |  |  | -0.008 | 0.010 | -0.825 |  |  |  |  |  |  |
| Wahlund: Temporal |  |  |  | 0.002 | 0.016 | 0.154 |  |  |  |  |  |  |
| Wahlund: Infratentorial |  |  |  | 0.014 | 0.012 | 1.160 |  |  |  |  |  |  |
| gFA |  |  |  |  |  |  | -0.001 | 0.002 | -0.451 |  |  |  |
| gMD |  |  |  |  |  |  |  |  |  | 0.000 | 0.002 | -0.219 |

**Cardiovascular disease models**

Table S22: Standardized beta estimates for the final models predicting CRT SD in subsamples based on CVD status

|  |  | CVD |  | No CVD | | |
| --- | --- | --- | --- | --- | --- | --- |
|  | *b* | *se* | *p*-value | *b* | *se* | *p*-value |
| Sex | -14.367 | 4.221 | 0.001 | -9.056 | 2.666 | 0.001 |
| Age | -1.452 | 3.033 | 0.633 | 0.549 | 1.880 | 0.770 |
|  |  |  |  |  |  |  |
| Hypertension | 0.921 | 4.343 | 0.832 | -0.396 | 2.793 | 0.887 |
| Diabetes | -3.730 | 5.764 | 0.518 | 0.001 | 5.030 | 1.000 |
| Cholesterol | -2.422 | 4.257 | 0.570 | 1.369 | 2.953 | 0.643 |
| Blood Circulation | 8.483 | 5.500 | 0.125 | 3.892 | 3.519 | 0.269 |
| CRT Mean | 0.278 | 0.023 | <.001 | 0.259 | 0.016 | <.001 |
|  |  |  |  |  |  |  |
| WMH Volume | -0.300 | 2.150 | 0.889 | 1.149 | 1.331 | 0.389 |
|  |  |  |  |  |  |  |
| Wahlund: Frontal | 5.923 | 5.949 | 0.321 | 4.670 | 3.781 | 0.217 |
| Wahlund: Parieto-Occipital | -7.701 | 5.315 | 0.149 | -5.342 | 3.743 | 0.154 |
| Wahlund: Basal Ganglia | -7.066 | 10.142 | 0.487 | 0.889 | 8.943 | 0.921 |
| Wahlund: Temporal | -2.842 | 18.393 | 0.877 | -2.620 | 13.371 | 0.845 |
| Wahlund: Infratentorial | 10.994 | 12.845 | 0.393 | 2.928 | 10.655 | 0.784 |
|  |  |  |  |  |  |  |
| gFA | -4.211 | 2.177 | 0.054 | -0.351 | 1.584 | 0.825 |
| gMD | -1.584 | 2.272 | 0.486 | -0.785 | 1.494 | 0.600 |

*Note:* Group 1 (CVD) = those with history of CVD or stroke (N=214); Group 2 (No CVD) = excluding all participants with CVD or stroke history (N=456). Covariate effects taken from the WMH model.

Table S23: Standardized beta estimates for the final models predicting CRT Mean in subsamples based on CVD status

|  |  | CVD |  | No CVD | | |
| --- | --- | --- | --- | --- | --- | --- |
|  | *b* | *se* | *p*-value | *b* | *se* | *p*-value |
| Age | 23.536 | 9.823 | 0.017 | 18.582 | 6.311 | 0.003 |
| Sex | 15.698 | 6.879 | 0.024 | 6.847 | 4.424 | 0.122 |
|  |  |  |  |  |  |  |
| Hypertension | -5.576 | 9.962 | 0.576 | -0.019 | 6.589 | 0.998 |
| Diabetes | 13.608 | 13.211 | 0.304 | 17.745 | 11.838 | 0.135 |
| Cholesterol | 12.892 | 9.739 | 0.187 | 1.887 | 6.969 | 0.787 |
| Blood Circulation | -10.159 | 12.680 | 0.424 | 1.081 | 8.313 | 0.897 |
| CRT SD | 1.464 | 0.124 | <.001 | 1.442 | 0.088 | <.001 |
|  |  |  |  |  |  |  |
| WMH Volume | 9.480 | 4.891 | 0.054 | 5.017 | 3.135 | 0.110 |
|  |  |  |  |  |  |  |
| Wahlund: Frontal | 9.086 | 13.723 | 0.509 | 12.241 | 8.871 | 0.168 |
| Wahlund: Parieto-Occipital | 16.952 | 12.252 | 0.168 | 7.870 | 8.799 | 0.372 |
| Wahlund: Basal Ganglia | -19.882 | 23.352 | 0.396 | -12.315 | 20.984 | 0.558 |
| Wahlund: Temporal | 13.422 | 42.367 | 0.752 | 38.080 | 31.336 | 0.225 |
| Wahlund: Infratentorial | 20.617 | 29.612 | 0.487 | 6.544 | 25.011 | 0.794 |
|  |  |  |  |  |  |  |
| gFA | 14.275 | 4.860 | 0.004 | 10.707 | 3.685 | 0.004 |
| gMD | 3.559 | 5.194 | 0.494 | 7.607 | 3.506 | 0.031 |

*Note:* Group 1 (CVD) = those with history of CVD or stroke (N=214); Group 2 (No CVD) = excluding all participants with CVD or stroke history (N=456). Covariate effects taken from the WMH model.

Table S24: Standardized beta estimates for the final models predicting CRT CV in subsamples based on CVD status

|  |  | CVD |  | No CVD | | |
| --- | --- | --- | --- | --- | --- | --- |
|  | *b* | *se* | *p*-value | *b* | *se* | *p*-value |
| Age | -0.020 | 0.006 | 0.002 | -0.014 | 0.004 | 0.001 |
| Sex | -0.001 | 0.004 | 0.827 | 0.001 | 0.003 | 0.635 |
|  |  |  |  |  |  |  |
| Hypertension | 0.000 | 0.006 | 0.962 | -0.001 | 0.004 | 0.851 |
| Diabetes | -0.005 | 0.009 | 0.600 | 0.001 | 0.008 | 0.875 |
| Cholesterol | -0.003 | 0.006 | 0.622 | 0.002 | 0.005 | 0.652 |
| Blood Circulation | 0.014 | 0.008 | 0.079 | 0.006 | 0.005 | 0.264 |
|  |  |  |  |  |  |  |
| WMH Volume | 0.002 | 0.003 | 0.541 | 0.002 | 0.002 | 0.274 |
|  |  |  |  |  |  |  |
| Wahlund: Frontal | 0.012 | 0.009 | 0.164 | 0.009 | 0.006 | 0.104 |
| Wahlund: Parieto-Occipital | -0.011 | 0.008 | 0.167 | -0.009 | 0.006 | 0.113 |
| Wahlund: Basal Ganglia | -0.016 | 0.015 | 0.291 | -0.001 | 0.014 | 0.949 |
| Wahlund: Temporal | 0.001 | 0.027 | 0.965 | 0.001 | 0.021 | 0.960 |
| Wahlund: Infratentorial | 0.019 | 0.019 | 0.308 | 0.008 | 0.016 | 0.631 |
|  |  |  |  |  |  |  |
| gFA | -0.004 | 0.003 | 0.195 | 0.001 | 0.002 | 0.754 |
| gMD | -0.001 | 0.003 | 0.847 | 0.000 | 0.002 | 0.902 |

*Note:* Group 1 (CVD) = those with history of CVD or stroke (N=214); Group 2 (No CVD) = excluding all participants with CVD or stroke history (N=456). Covariate effects taken from the WMH model.
